# Supplementary material for: Probabilistic tsunami forecasting for early warning
Source: Nat Commun. 2021 Sep 28;12:5677. doi: 10.1038/s41467-021-25815-w (PMC8479076; doi:10.1038/s41467-021-25815-w)
Supplement: Supplementary file 1 — Supplementary Information [file 41467_2021_25815_MOESM1_ESM.pdf]

Supplementary Information for

Probabilistic Tsunami Forecasting for Early Warning

J. Selva [1], S. Lorito [1], M. Volpe [1], F. Romano [1], R. Tonini [1], P. Perfetti [1], F. Bernardi [1], M. Taroni [1], A. Scala [2], A. Babeyko [3], F. Løvholt [4], S.J. Gibbons [4], J. Macías [5], M.J. Castro [5], J.M González-Vida [5], C. Sánchez-Linares [5], H.B. Bayraktar [2,1], R. Basili [1], F.E. Maesano [1], M.M Tiberti [1], F. Mele [1], A. Piatanesi [1], A. Amato [1]

1 Istituto Nazionale di Geofisica e Vulcanologia, Italy
2 Department of Physics “Ettore Pancini”, University of Naples, Naples, Italy.
3 German Research Centre for Geosciences (GFZ), Potsdam, Germany
4 Norwegian Geotechnical Institute (NGI), Oslo, Norway
5 Grupo EDANYA, Universidad de Málaga, Málaga, Spain

Table of Contents

Table of Contents 2
Supplementary Notes 3
Supplementary Note 1. Earthquake real-time magnitude and location evaluation at CAT-INGV Tsunami Service Provider (TSP) ... 3
Supplementary Note 2. Mapping PTF into alert levels ... 4
Supplementary Note 3. Decision Matrix and Alert levels in NEAMTWS CAT-INGV Tsunami Service Provider (TSP) ... 5
Supplementary Note 4. The 2003 Zemmouri-Boumerdes earthquake: source models, tsunami simulations and tsunami intensity data ... 6
Supplementary Note 5. NEAMWAVE 2017 case study ... 6
Supplementary Note 6. Retrieval of tsunami Intensity data for testing ... 7
Supplementary Note 7. Results of statistical testing ... 8
Supplementary Figures 10
Supplementary Figure 1. Map of Forecast Points in the Mediterranean Sea. ... 10
Supplementary Figure 2. Tide gauge data (continues on the next 2 pages). ... 11
Supplementary Figure 3. Run-up data (continues on the next 2 pages). ... 14
Supplementary Figure 5. Testing tsunami wave amplitude forecasts. ... 17
Supplementary Figure 6. Testing PTF for NEAMWave17 M8.5 scenario. ... 18
Supplementary Figure 7. DM and PTF alert levels for small perturbations to the magnitude. ... 19
Supplementary Figure 8. Modelled wave amplitude for the 2003 Zemmouri-Boumerdes tsunami. ... 20
Supplementary Figure 9. Modelled maximum wave amplitude for the 2003 Zemmouri-Boumerdes tsunami. ... 21
Supplementary Tables 22
Supplementary Table 1. Acronyms and abbreviations. ... 22
Supplementary Table 2. Early-Est (EE) location and magnitude parameters. ... 23
Supplementary Table 3. 2003 Zemmouri-Boumerdes fault geometry and mechanisms. ... 24
Supplementary Table 4. Ensemble members and computational times. ... 25
Supplementary Table 5. Fault geometry and mechanism for all the events in the testing dataset. ... 26
Supplementary Table 6. Tide-gauge stations. ... 27
Supplementary Table 7. P-Value of tests on strike, dip, and rake angles. ... 28
Supplementary Table 8. CAT-INGV NEAMTWS Decision Matrix (DM). ... 29
Supplementary Table 9. Correct/missed/false alarms for DM, ENV, BMS and PDF.. 30
Supplementary References 31

Supplementary Notes

Supplementary Note 1. Earthquake real-time magnitude and location evaluation at CAT-INGV Tsunami Service Provider (TSP)

To produce a uniform treatment of all case studies, we approximately reproduce the standard operating conditions of CAT-INGV for all the hind-casting experiments described in the main text. Early-Est (henceforth EE) is the full automatic locator software of CAT-INGV for M ≥ 5.5. The hypocentral location parameters and their covariance matrix are computed through the EE in offline mode, i.e.: using a fixed waveform package instead of a real-time stream of data.

In order to reproduce the automatic procedure at CAT-INGV described in 8 and the EE algorithm described in [1–3] we operate as follow: i) we retrieved from IRIS and EIDA/ORFEUS (Supplementary Data 2) seismic waveforms 1800s long and starting ~10' before the event origin time; ii) we select only stations that are available in real-time and that are acquired, if available, by EE; iii) we first select the first 60s of waveform and we run NonLinLoc; iv) we iteratively add 60s of waveform and run EE; v) The first run that results in an event location will produce the first association; vi) we continue till we add 8 minutes of data after the first association.

Based on the performance analysis presented by Bernardi et al. [3], and on further unpublished analyses, CAT-INGV uses for alerting purposes the estimates for location and magnitude obtained 5' after the first association, which offer a satisfying trade-off between earliness and accuracy. The preferred magnitude type depends on the best guess value for M_{wp}. In particular: if M_{wp} <5.8, m_b is chosen; if M_{wp} ≥ 7.2, M_{wpd} is chosen; for intermediate values, M_{wp} itself is preferred. A minimum number of 6 observations is also required. We here adopt all the CAT-INGV selection criteria, without deepening into how these choices may influence PTF uncertainty, which is left for future studies.

Since archived datasets are always more complete than those available in real-time and do not suffer from the same delays, it can be assumed that the solutions obtained from these data are slightly more accurate and precise than those obtained in real-time for the same events. Hence, as a term of comparison to evaluate the potential extent of this issue, in Supplementary Table 2 we also report the solutions obtained 2' and 8' after the first association, using shorter and longer time-series, respectively, than that obtained at 5'. These ranges possibly provide a rough overview of the order of magnitude of the real-time uncertainties related to data streaming.

Uncertainties related to the magnitude and epicentral location and the all algorithm are extensively described in EE user guide available at http://alomax.free.fr/projects/early-est/early-est_users_guide.pdf

Supplementary Note 2. Mapping PTF into alert levels

The distribution $h_E(x,p,t)$ can be translated into alert levels at any time t, connecting scientific forecasts to risk reduction measures. The uncertainty on the forecast, as evaluated to the best of the scientific knowledge at time t, is properly communicated through $h_E(x,p,t)$. The rules for converting the forecast $h_E(x,p,t)$ into alert levels should be based on political decisions. As a consequence, here, rather than suggesting any specific approach or expressing our preference, we only aim to make things more tangible by briefly discussing possible alternatives, highlighting some practical consequences of specific choices.

The NEAMTWS uses three alert levels: Information/Green-No Alert, Advisory/Orange Alert, and Watch/Red Alert. Advisory/Orange and Watch/Red alert levels roughly correspond to marine/near-coast tsunami threat and significant inundation, respectively, and are associated with tsunami run-ups smaller/greater than 1 m and wave amplitudes smaller/greater than 0.5 m.

To convert $h_E(x,p,t)$ into alert levels, we first assign a correspondence between intervals of tsunami intensity (in terms of run-up or wave amplitude) and alert levels. We adopt the following reference tsunami intensity intervals:

- Watch/Red: run-up > 1 m or wave amplitude > 0.5 m
- Advisory/Orange: 0.2 m < run-up ≤ 1 m or 0.1 m < wave amplitude ≤ 0.5 m
- Information/Green: run-up ≤ 0.2 m or wave amplitude ≤ 0.1 m

which corresponds to the definition of alert levels in NEAMTWS (see Supplementary Note 3).

The threshold for discriminating a negligible from a non-negligible tsunami has been arbitrarily set to 0.2 m run-up (or 0.1 m wave amplitude). It should be probably more accurately defined in the future, possibly based on considerations on the associated risk. The information level in the NEAMTWS is activated for earthquakes with $M_w \geq 5.5$ and with the hypocenter not too deep and not too far inland (as in the DM adopted by CAT-INGV, see Supplementary Table 8). The same rules can be adopted also for whatever else method, including PTF.

Based on these intervals, without attempting to be exhaustive, we can define at least two classes of conversion methods.

The first class of methods is based on comparing an intensity extracted from the probability density function $dh_E(x,p,t)$ and the defined intervals, assigning the corresponding alert level. This can be done either using a central tendency value (e.g., the mean) or using a different statistic (e.g., a percentile). This approach, which recalls the current practice at TEWSs, is adopted in the main text.

The use of the central tendency recalls the common practice of selecting the best-matching scenario (BMS). However, the PTF central tendency simultaneously applied to all locations does not necessarily correspond to the best-guess source in each location.

The use of a percentile y corresponds to the definition of an exceedance probability threshold $P_{th} = 1 - y$ (e.g. $P_{th} = 0.05 \equiv 5\%$) applied to the hazard curve $h_E(x,p,t)$. In other words, a "design" intensity X_{th} is selected so that $P(x > X_{th}|E;p) = P_{th}$, and it is used to assign the alert level. This procedure resembles those adopted for earthquake and tsunami building codes starting from probabilistic hazard assessments [4]. Noteworthy, defining X_{th} corresponds to select a "precautionary" level in alert level assignment, since P_{th} represents the maximum probability of observing a tsunami intensity larger than the

maximum intensity of each alert level. In other words, it represents the maximum probability of underestimating the alert level. The smaller the P_{th} value, the more precautionary the selected alert level. In the limit of $P_{th} = 0$, the highest alert level is always selected. Of course, P_{th} is related also to the expected rate of false alarms. If X_{th} is close to the threshold between different alert levels (e.g. 0.5 m), $P(X > X_{th} \approx 0.5\, m|E, p) = P_{th}$ corresponds to an expected rate of false alarms of $1 - P_{th}$ (for $P_{th} = 0.05$, a rate of 95% of false alarms). Thus, the more precautionary the choice, the higher the expected number of false alarms.

The second class of methods is based on the probability of observing tsunami intensities belonging to different intervals. It can be evaluated by integrating $dh_E(x, p, t)$ over each interval. In analogy with the procedure described above, in each location, the alert level can be assigned based on the most likely tsunami intensity range, or other criteria. For example, the selection of the most likely intensity interval minimizes the number of times that intensity values outside this range are observed. In other words, the number of wrong assignments of the alert level is minimized.

These classes of methods do not take into account vulnerability and exposure at a site. A new set of methods could be defined considering the risk consequent to a given tsunami intensity. For example, P_{th} might be related to the consequent risk, representing the maximum probability of observing a loss larger than a predefined level. Thus, the definition of acceptable risk can in principle defined in the ground of consequent damages, potentially differentiating acceptable hazards in the different location p due to, for example, local exposure, or for different target actions.

Supplementary Note 3. Decision Matrix and Alert levels in NEAMTWS CAT-INGV Tsunami Service Provider (TSP)

NEAMTWS TSPs base tsunami forecasting on a decision matrix (DM), to assess tsunami alert levels for potentially threatened coastal locations in case of a coastal or submarine strong earthquake (of magnitude 5.5 or greater for the NEAMTWS).

A DM is a look-up table used for converting the real-time earthquake parameter estimates (typically hypocentral location and magnitude) into tsunami alert levels. DMs are generally distance-based, so the potential threat decreases with the distance from the earthquake source. Depending on the local/regional rules, the alert levels apply to pre-defined coastal stretches or sets of discrete forecast points (oddly distributed along the Mediterranean coasts and not coincident with the ones used in this paper). In the NEAMTWS, the forecast points generally correspond to the locations of sea-level instruments or to highly exposed/vulnerable locations. One of the NEAMTWS DMs, namely the one currently in use at CAT-INGV, is presented in Supplementary Table 8.

Tsunami ranges (Local, Regional, Basin-wide) define the distances between the earthquake epicentre and the forecast points, as circles of fixed radius (see Supplementary Table 8). In NEAMTWS, hence in this DM, three different alert levels are used:

- Information/Green: meaning that no tsunami is expected to impact the coast because the occurred seismic event has been evaluated either as a non-

tsunamigenic event or as not able to produce such a tsunami wave representing a relevant threat to the exposed coasts;

- Advisory/Orange: meaning that the detected seismic event could produce a tsunami wave amplitude up to 0.5 m in front of the coast and inundation of the coastline (run-up) up to 1 meter above the sea level; in essence, this alert level indicates a marine and near-coast tsunami threat;
- Watch/Red: meaning that the tsunami wave amplitude is expected to be larger than 0.5m in front of the coast and the run-up higher than 1 m above the sea level; thus, this alert level is to be issued when potentially significant inundation is expected.

This DM is based on analysis of historical events, tsunami modelling, and expert judgment and is worst-case oriented. In this sense, it is quite conservative, as such, for example, it tends to generate false basin-wide alerts in case of larger earthquake magnitudes (M>7). Additionally, it takes into account neither the source mechanism and directivity of tsunami generation, nor the azimuthal anisotropy of the tsunami propagation on a complex bathymetry. So, as already noted in the main text, despite the conservative approach, this DM may for example result in some cases in alert underestimation when eventual tsunami energy focusing occurs.

Supplementary Note 4. The 2003 Zemmouri-Boumerdes earthquake: source models, tsunami simulations and tsunami intensity data

PTF accuracy has been quantitatively tested by considering a total of 12 fault plane solutions for the 2003 Zemmouri-Boumerdes earthquake. Among the 12 solutions (Supplementary Table 3), 5 are based on seismic moment tensor estimation and 7 are obtained by separately and jointly inverting seismic and geodetic data [5–11].

For the 7 finite fault inversions, forward tsunami simulations have been performed first calculating the initial (instantaneous) sea-level displacement [12], then using the nonlinear shallow-water Tsunami-HySEA GPU code [13]. The tsunami propagation simulations were carried out on a topo-bathymetric grid covering the entire Mediterranean basin and having resolution equal to 30 arc-sec (SRTM30+, https://topex.ucsd.edu/WWW_html/srtm30_plus.html); the duration of each simulation was fixed to 4 hours. The values at the forecast points are obtained using the Green's law to extrapolate the maximum elevations in front of the coast (i.e. nominally at 1 m) from the values recorded on the 50 m isobath. So, except for the fact that here simulations start from a complete initial condition, and that no uncertainty distribution is applied in the end to the tsunami maxima, these simulations use the same scheme as for the scenarios composing the ensembles used for the PTF. The results are reported in Supplementary Figures 8 and 9.

Supplementary Note 5. NEAMWAVE 2017 case study

To test larger magnitudes in the Mediterranean Sea, we implemented the NEAMWave17 ICG/NEAMTWS Mw 8.5 exercise scenario. This tsunami scenario was one of the scenarios proposed for the NEAMWave17 exercise that is periodically organized by

ICG/NEAMTWS to test the alert system for the member states that subscribed to receive tsunami alert messages produced by TSPs in NEAMTWS [14].

This scenario was prepared jointly by CAT-INGV and NOA–HLNTWC and refers to a strong tsunami event affecting the eastern Mediterranean Sea. The tsunami is supposed to be generated by a great subduction earthquake of magnitude 8.5, occurring south of Zakynthos Island, in the western segment of the Hellenic Arc. Several large earthquakes have been reported to affect this area in the past, such as 1953, 1867 1767 and 1638 events with magnitudes around 7.5; in the present scenario, while the tectonic framework resembles in terms of earthquake magnitude the very large event of 21 July 365 AD, the location of the earthquake is shifted north-westward along the Hellenic Arc.

Earthquake rupture area (~40.000 km^2) extends in NW-SE direction for almost 400 km from offshore Cephalonia Island to the west of Peloponnese (Supplementary Figure 6a); the fault geometry is modelled with a mesh of triangular elements around the epicentre (with variable strike and dip following a 3D model of the subduction zone). The rupture area is chosen following Strasser et al. [15]; the slip value is defined homogenous all over the fault surface (equal to 6.5 meters considering a rigidity value of 26 GPa), whereas the slip direction is compliant with a purely thrust mechanism (rake=90°). The ensuing tsunami is modelled exactly as for the Zemmouri-Boumerdes finite faults, except for a longer simulated time of 8 hours.

As in the Maule case study (see Main Text), for this magnitude level the PTF ensemble is formed only by large magnitude subduction interface sources (Supplementary Table 4), which include scenarios with shallow slip amplification (mimicking tsunami earthquakes; see Main Text). The comparison between PTF and the simulated scenario shows a good agreement (Supplementary Figure 6b,c,d). However, we note that these results, from the statistical point of view, are less relevant than the other case studies presented, being PTF scenarios and synthetic data based on the same fault plane and similar simulation schemes.

Supplementary Note 6. Retrieval of tsunami Intensity data for testing

The testing data for the 2003 Zemmouri-Boumerdes are the published peak-to-trough maximum tsunami wave heights from tide-gauges [16]. Taking into account that the tsunami amplitudes are relatively small, the process can essentially be considered linear. Because the PTF makes use of positive simulated surface elevation maxima, a transformation of the measured wave data was necessary. Consequently, to make them roughly comparable with the positive elevations maxima obtained from the simulations, which are normalized with Green's law to 1 m depth, we halve the measured peak-to-through maxima (the tide-gauges are placed at shallow depths close to the shore). While this is quite a rough comparison between quantities of different nature, we note that Power et al. [17], similarly to others, see references therein, utilize this property for run-up data (see also below), and a similar expression is expected to approximately hold close to the shoreline for long waves.

The testing data for all the other events considered (same as for the focal mechanism, for a total of 12 events, see Fig. 4A and Supplementary Table 2) are retrieved from tide gauge sensors within 400 km from the epicentre (Supplementary Figure 3, Supplementary Dataset 3, Supplementary Table 6). Half of the maximum peak-

to-trough values are extracted from time-series de-tided with a robust LOWESS algorithm (locally weighted scatter plot smoothing) technique. As mentioned above for the Zemmouri-Boumerdes tsunami, halving the double-amplitude is a way to extract more robust estimates than we would obtain by just using amplitudes for these very small tsunami signals as compared to the tidal range, and obtained from records with a poor signal-to-noise ratio.

For the 2010 Maule, 2018 Kos-Bodrum, 2020 Samos-Izmir event, we could also utilize run-up data [18–20] (Fig. 5 and Supplementary Figure 4). Before using it for comparison, also the run-up is halved, consistently with the NEAMTWS definition that establishes a correspondence between twice the elevation in front of the coast and the run-up (see Supplementary Notes 2 and 3). This is a quite common practice, often used the other way around for converting amplitudes into run-ups, as discussed for example by Power and co-authors [17]. It is clear that more extensive, even local, calibration, would always be necessary for obtaining a more accurate correspondence between offshore and onshore tsunami waves. A similar procedure is adopted for the observations of the Maule tsunami, including run-up and tide-gauge. Specifically for the 2010 Maule case, were available also deep-sea sensors tsunami observations (DART buoys); in this case, the test has been performed by using the maximum wave amplitudes recorded by DARTs.

Supplementary Note 7. Results of statistical testing

Both PTF source model and tsunami forecasts are tested against observations. For these tests, all the relevant information about the PTF application can be found in Supplementary Table 2 (EE estimation), Supplementary Table 4 (ensemble size), and Supplementary Dataset 5 (PTF results).

For the PTF source model, the testing is concentrated on the focal mechanism estimation, as magnitude and hypocentre are estimated through standard procedures discussed in literature [3,20]. The details of the testing procedures for focal mechanisms are discussed in Methods. Focal mechanisms for the 13 events in the testing dataset (Fig. 5A) are tested both individually and collectively, both selecting a preferred fault plane and considering both conjugate mechanisms. Observations for all the earthquakes are the revised double-couple solutions from the Global CMT catalogue (G-CMT, https://www.globalcmt.org/; [21,22]) and the rapid solutions from Quick Regional CMT (QRCMT, http://autorcmt.bo.ingv.it/quicks.html; [23]). All these data are reported in Supplementary Table 5. For the Zemmouri-Boumerdes, as several moment tensors and finite-fault model estimates are available in the literature (Supplementary Table 3), we additionally tested the PTF source model against all these estimates. All the results are reported in Supplementary Table 7, and the PTF source model is never rejected at standard confidence levels ($\alpha = 0.05$).

For the tsunami forecasts, observations include data from the available tide-gauges within 400 km from each epicentre (Supplementary Fig. 2, Supplementary Table 6; details in the extraction procedure in Supplementary Note 5) and from run-up surveys (Supplementary Figure 3, available for the 2017 Mw 6.6 Kos-Bodrum and the 2020 Mw 7.0 Samos-Izmir

earthquakes [18–20]). For Maule, they include also deep-sea DART data [24,25] (Fig. 5, see Supplementary Note 5).

PTF results are accurate in all the cases where a measurable tsunami was observed: the Lefkada, Kos-Bodrum, Zakynthos, Ierapetra, and Samos-Izmir earthquakes, as well as Zemmouri-Boumerdes (Supplementary Figure 4). We point out that, for the Mw 6.5 strike-slip Lefkada earthquake, there is a systematic underestimation at the Crotone tide-gauge (revealed, for example, by the minor peak at negative intensities). Nevertheless, the Fourier power spectrum of the Crotone recording features a peak at around 6-7 minutes [26], consistent with the excitation of a harbour eigenfrequency by the very short wavelength tsunami generated by the earthquake. A similar feature at the same location was also observed for the slightly larger 2018 Mw 6.8 Zakynthos earthquake [27] (Supplementary Figure 4). For both these case studies, as for the Zemmouri-Boumerdes event (see Main Text), the implemented uncertainty model is sufficient to account for these peaks at first order. The inclusion of high-resolution modelling, at least for specific locations subject to this kind of amplification, can reduce future forecast uncertainty, especially for such relatively low magnitude events.

For the Maule cast study, both coastal and deep-sea data are tested, and the tsunami forecasts are not rejected in both cases. For DART data, a tendency to the overestimation may be observed in the results of the test. As discussed in the Main Text, this may be due to a slight overestimation of the propagation-factor uncertainty, which accounts for both source and tsunami inundation simplifications (see Methods). Indeed, the source models included in the ensemble for large magnitudes are rather sophisticated and, at the same time, the sensitivity to source details in the far-field is less pronounced, the propagation in the deep ocean up to DARTs is relatively simple and substantially linear, and the resolution of the numerical simulations is sufficient. Then, the test of DART data is repeated also removing the propagation-factor uncertainty. In both cases the test is passed, but after the removal of propagation uncertainty the distribution of residuals is distributed is nicely distributed around 0. However, we note that this is a single case study. A fine tuning of the modelling of this kind of uncertainty should be target of future research.

The PTF results are equally good in estimating the earthquakes that did not generate any measurable tsunami (seven events: two events in Albania, Kasos Is., Gibraltar, Norcia, Lesbos Is., and Turkey earthquakes). In these cases, PTF consistently forecasts an essentially negligible tsunami (< 0.10 m) at all the observation points (Supplementary Figure 5). The statistical test fails only for Gibraltar event, probably due to a very small signal-to-noise ratio in the available measurements preventing the observation of a small (< 0.2 m) but not negligible tsunami.

Supplementary Figures

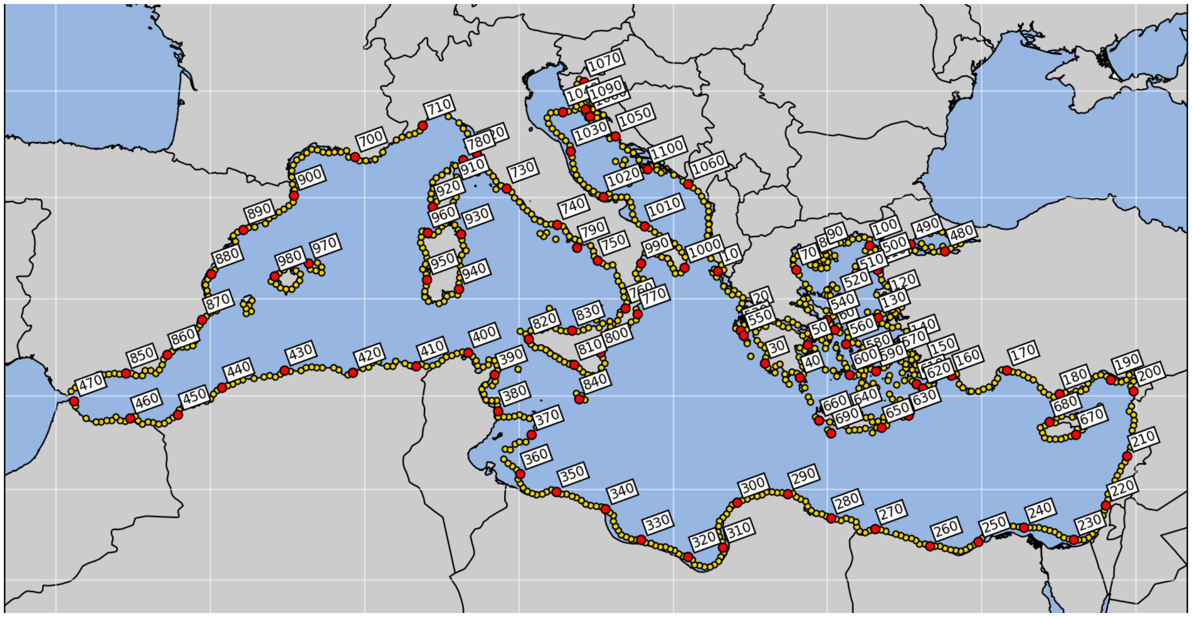

Supplementary Figure 1. Map of Forecast Points in the Mediterranean Sea. Forecast points for PTF output; the details (ID, longitude, latitude, and depth) of each forecast point are reported in Supplementary Data 1.

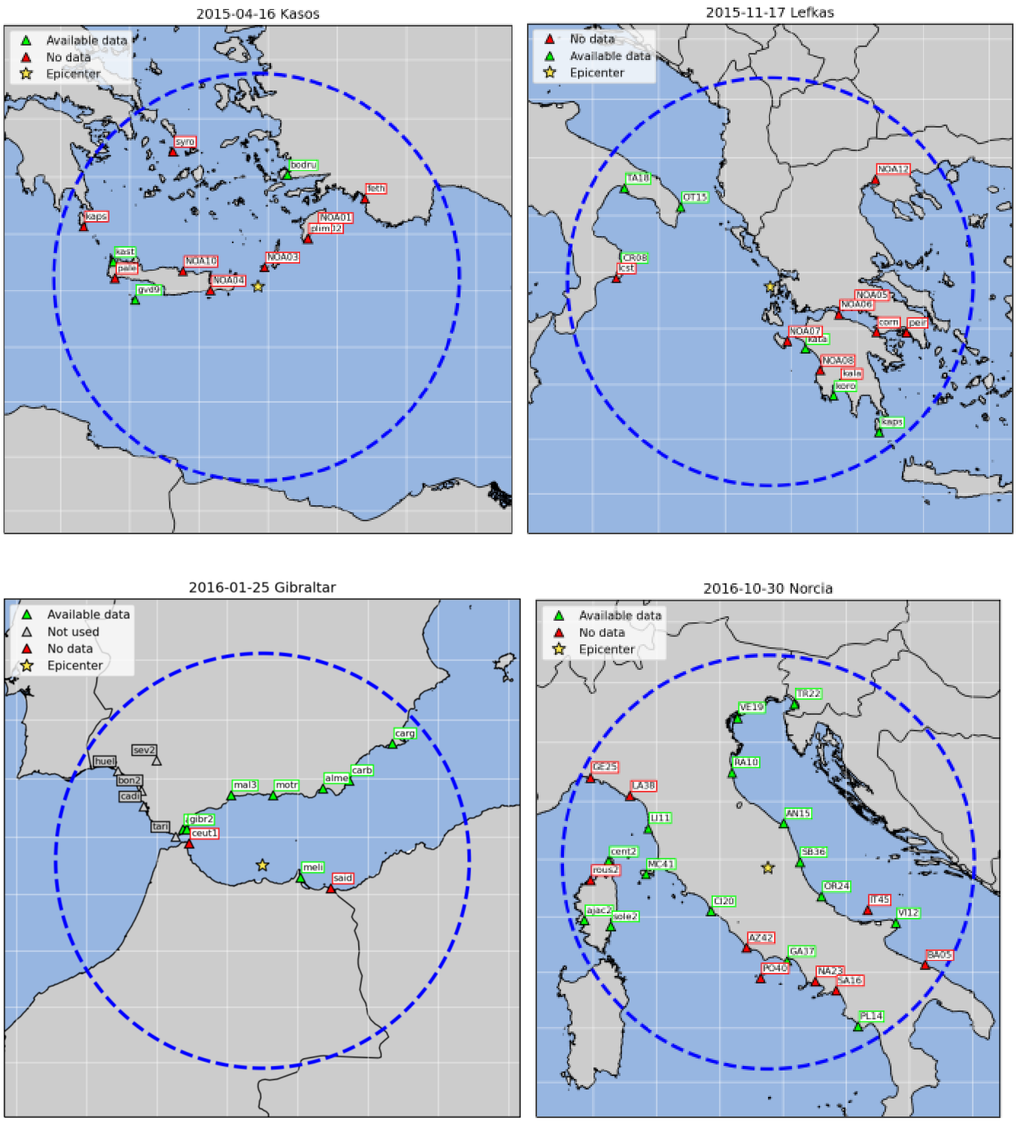

Supplementary Figure 2. Tide gauge data (continues on the next 2 pages).
Tide gauge sensors within 400 km from the epicenter for all the tested earthquakes (stations' lists in Supplementary Table 6, for run-up (49). In green the sensors for which the data were present at the time of the events, in red the ones not available, and in grey the sensors that lay outside the computational domain of PTF (and thus, not included even if available).

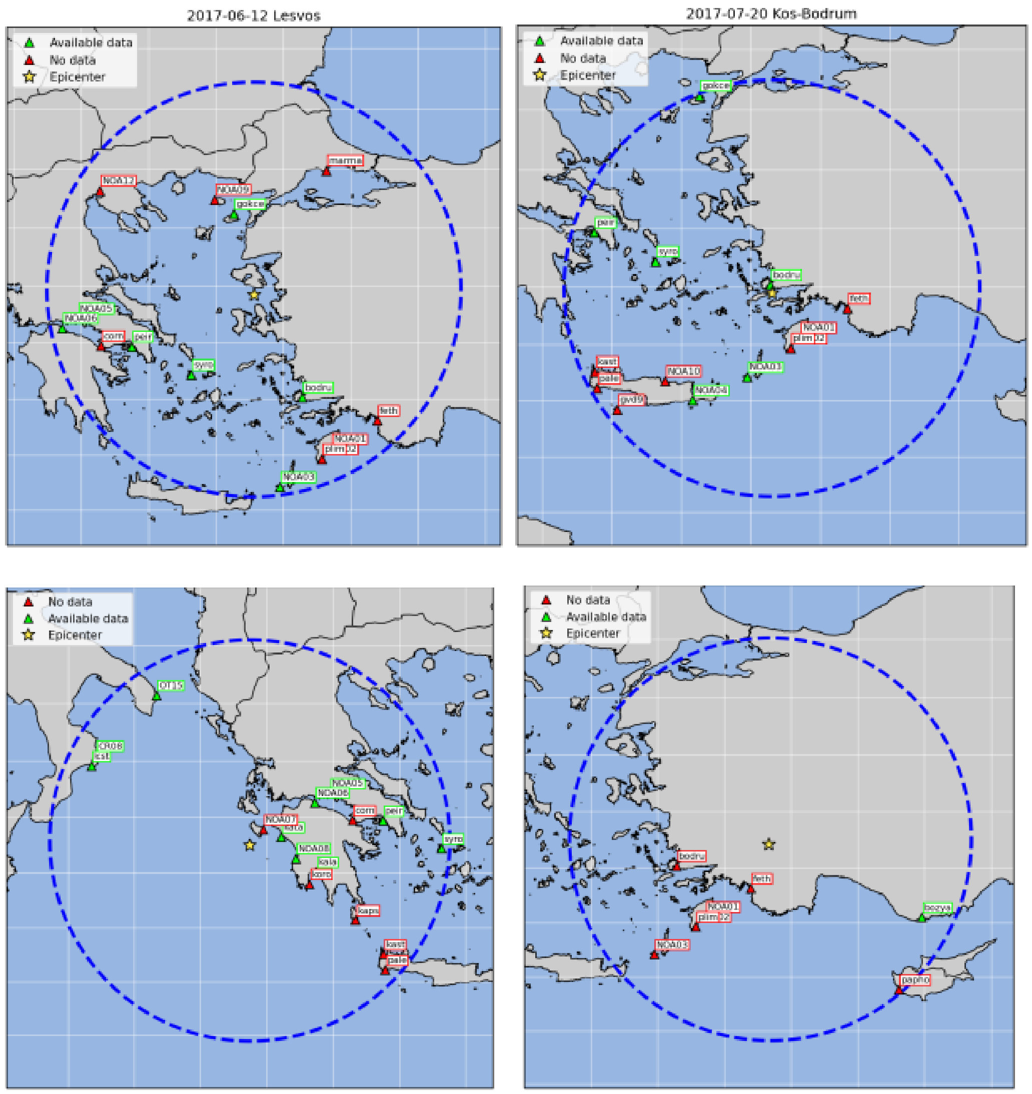

Supplementary Figure 2 (continued)

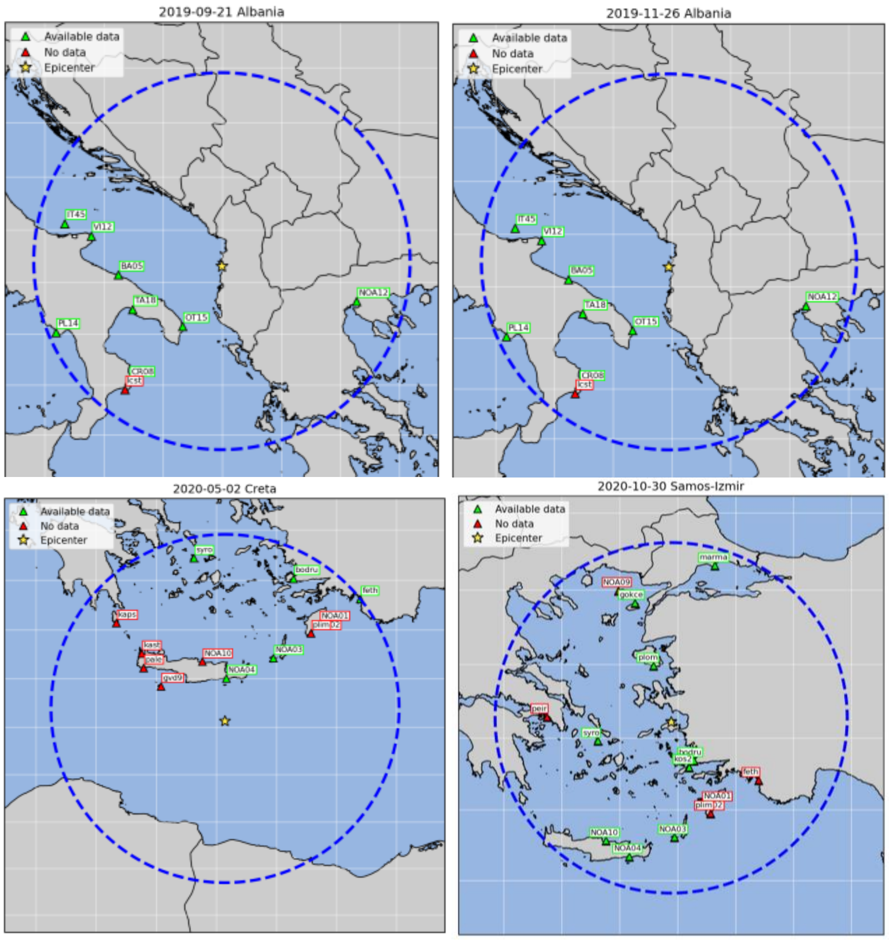

Supplementary Figure 2 (continued)

2017 Kos-Bodrum earthquake

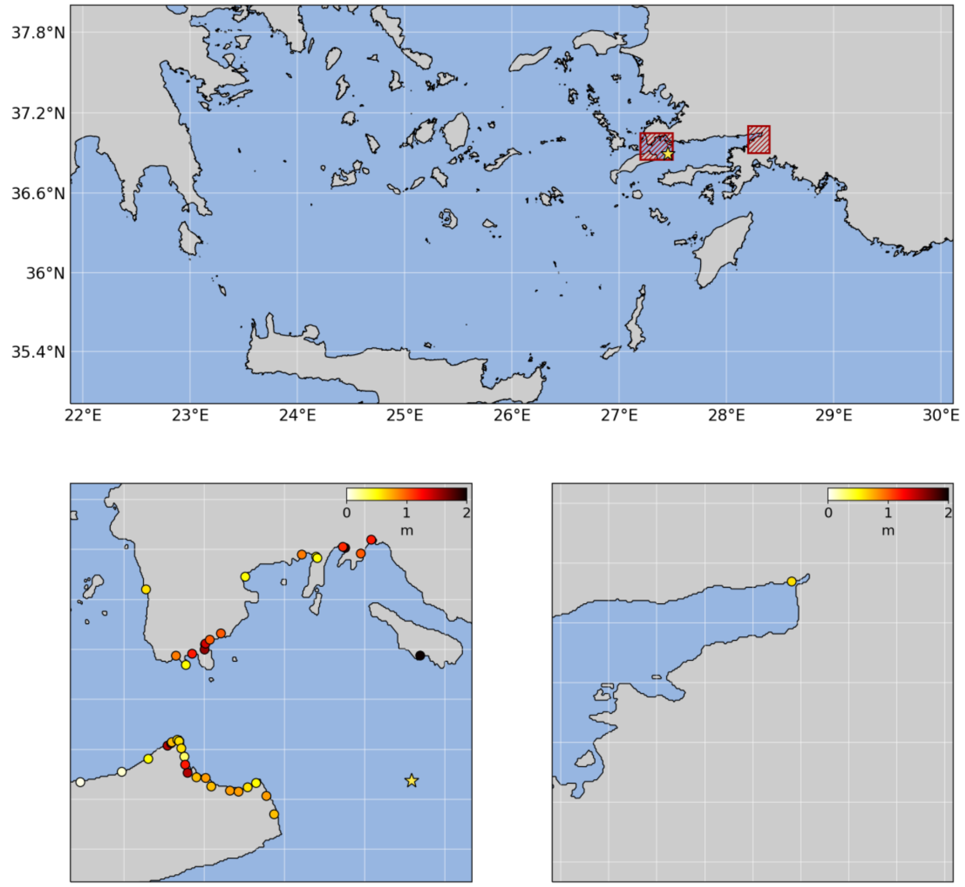

Supplementary Figure 3. Run-up data (continues on the next 2 pages).
Run-up observations from field surveys [18–20]. Colours are scaled to the intensity of the observed run-up

2020 Samos-Izmir earthquake

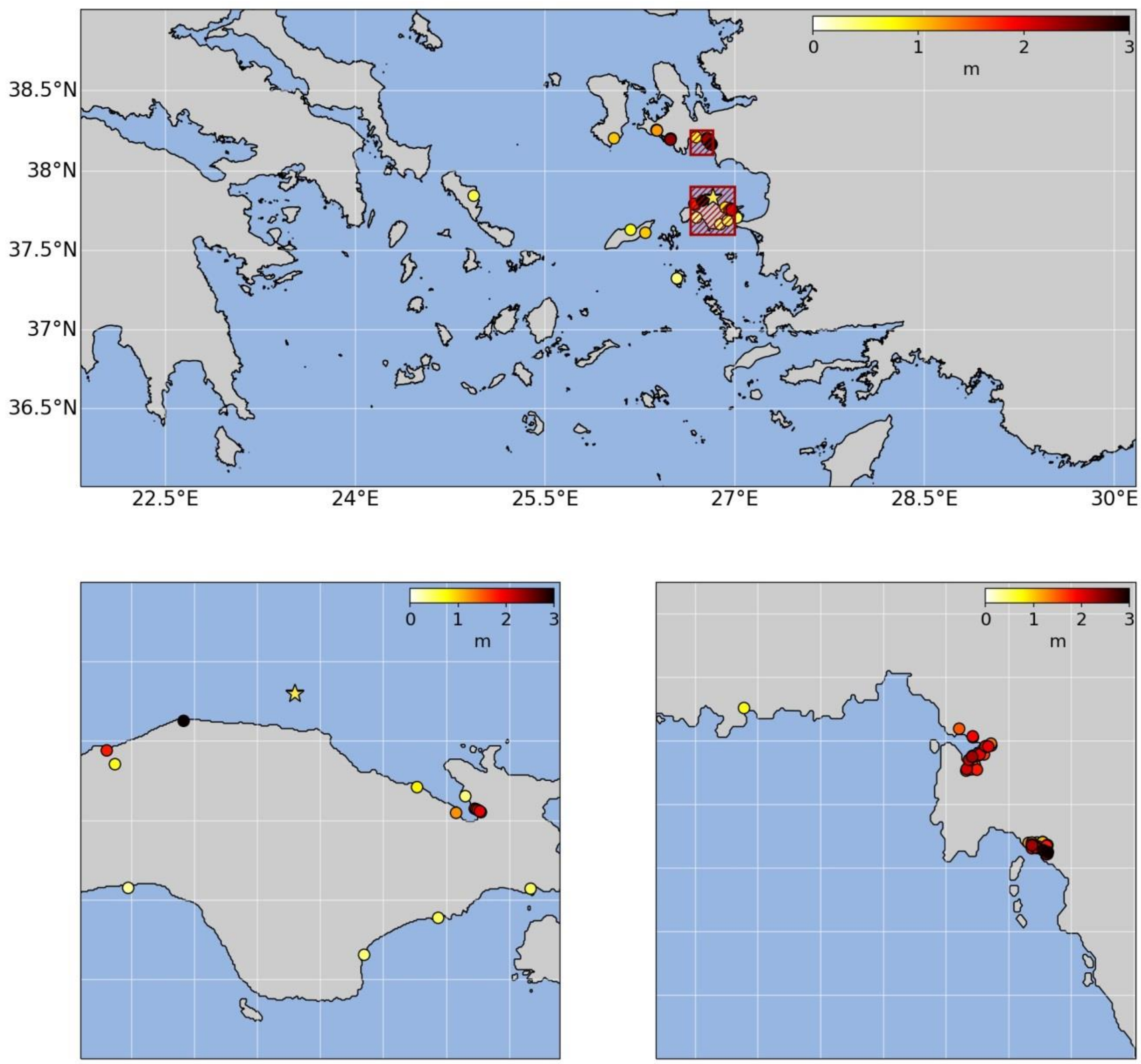

Supplementary Figure 3 (continued)

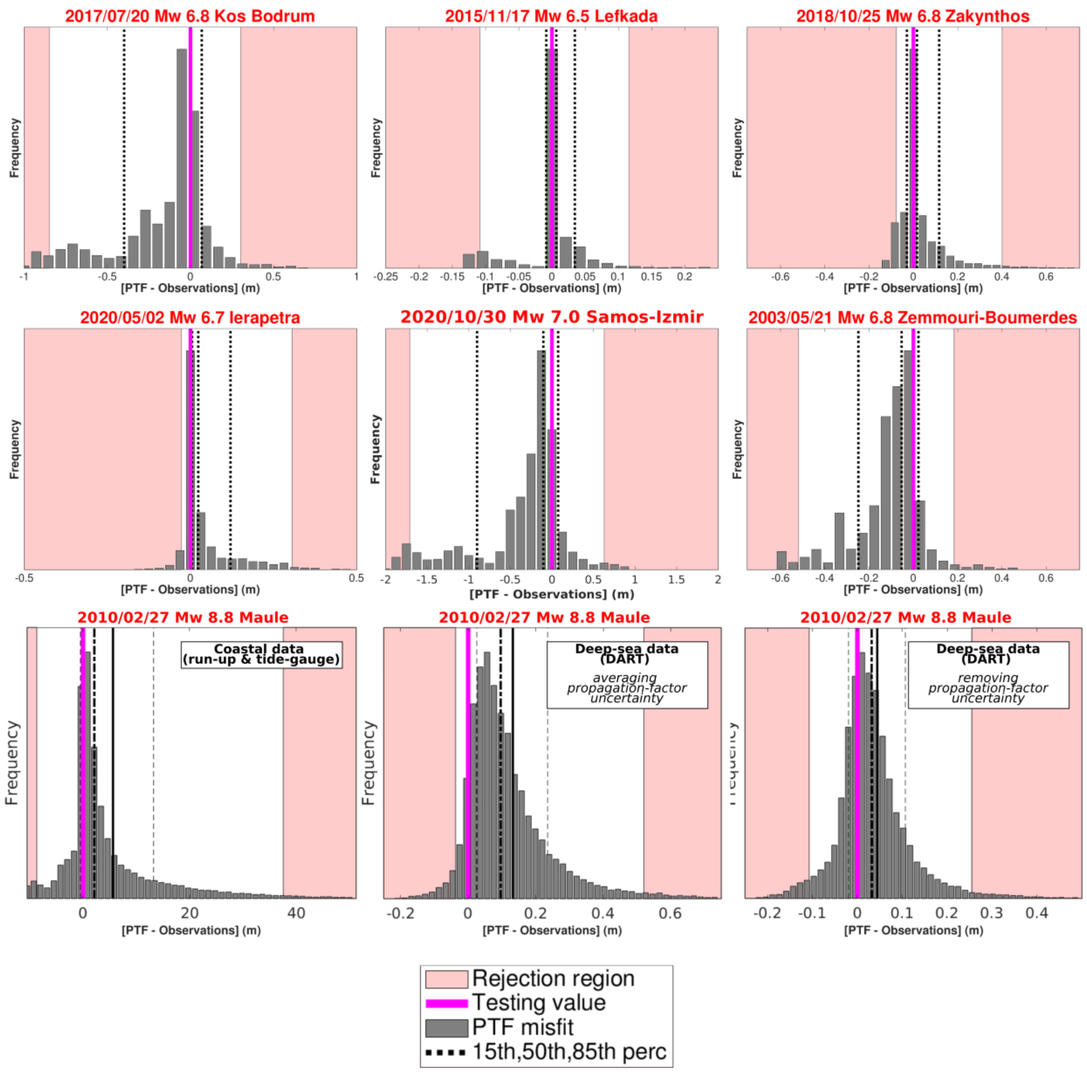

Supplementary Figure 4. Testing tsunami wave amplitude forecasts. Earthquakes with a recorded tsunami. Tsunami amplitudes are sampled from the PTF source ensemble and observations and staked for all observation points. To keep spatial correlations, the uncertainty on the propagation is averaged (see Methods).

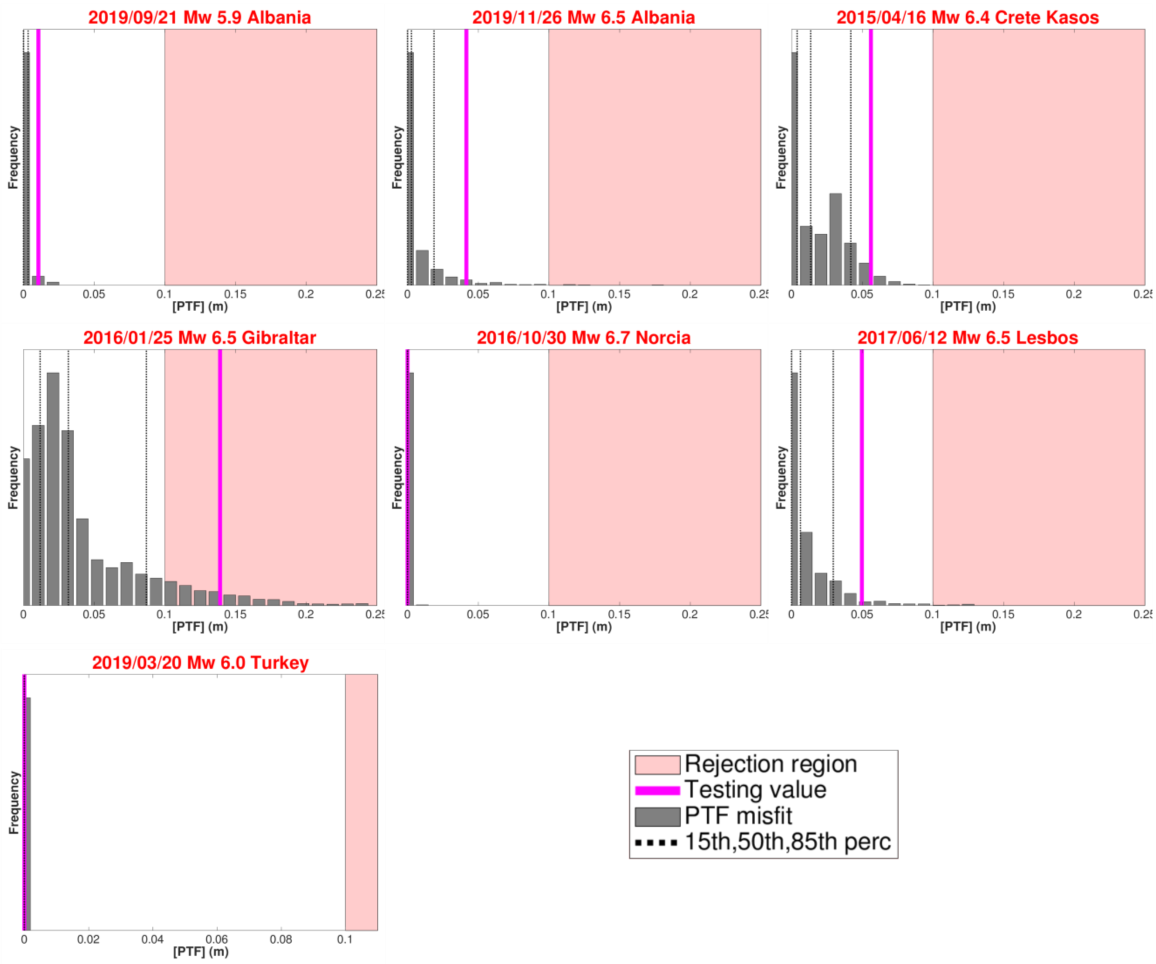

Supplementary Figure 5. Testing tsunami wave amplitude forecasts.

Earthquakes without a recorded tsunami. Tsunami amplitudes are sampled from the PTF source ensemble. To keep spatial correlations, the uncertainty on the propagation is averaged (see Methods).

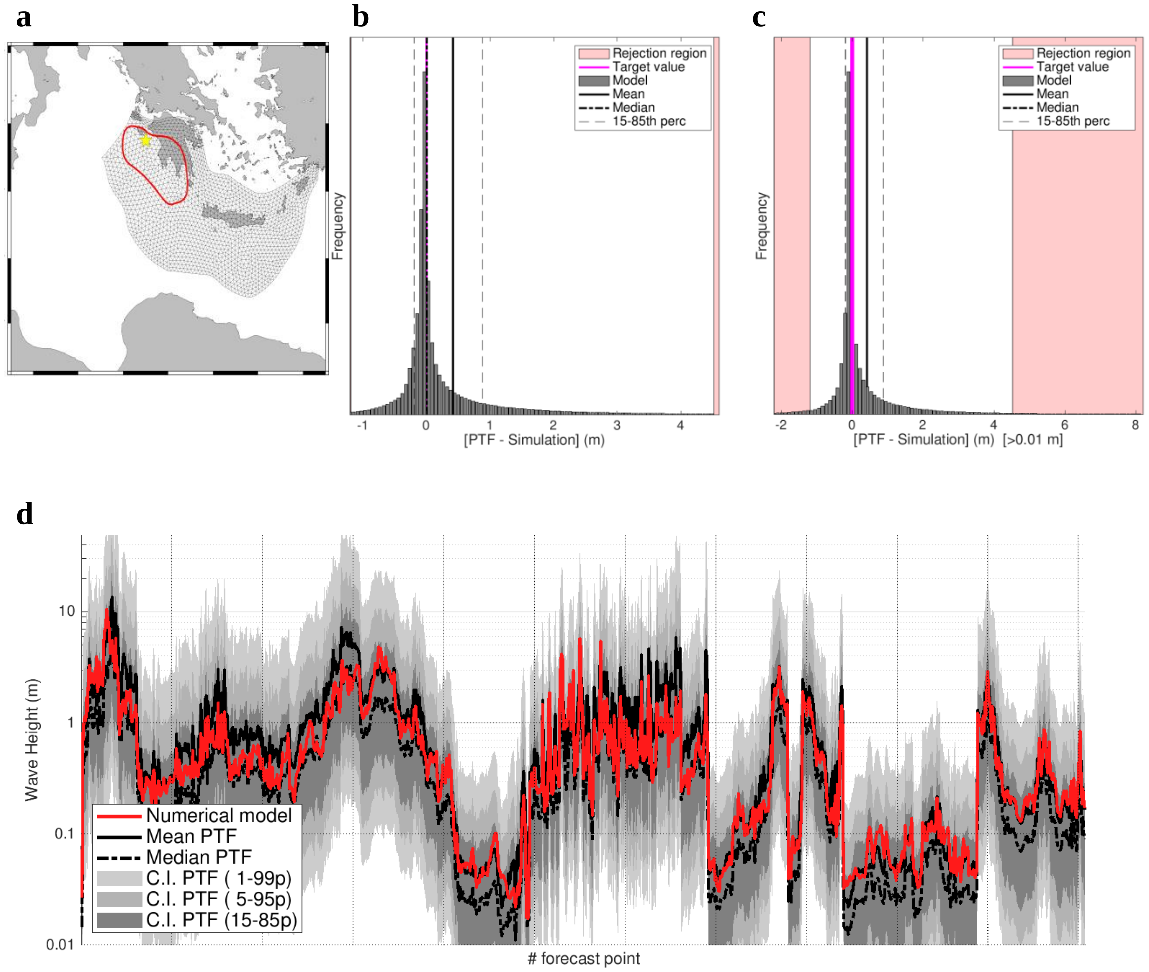

Supplementary Figure 6. Testing PTF for NEAMWave17 M8.5 scenario.

a) Sketch of the scenario earthquake rupture area (solid red line) selected on the Hellenic arc subduction zone (modelled as a triangular mesh, in black); yellow star represents the epicentre. b) Test of the accuracy of PTF tsunami wave amplitude forecast against the numerical simulation at all locations; c) as b), but in locations with a significant tsunami (> 0.01 m). d) Comparison between the tsunami maximum wave amplitude foreseen by numerical tsunami simulation and PTF statistics.

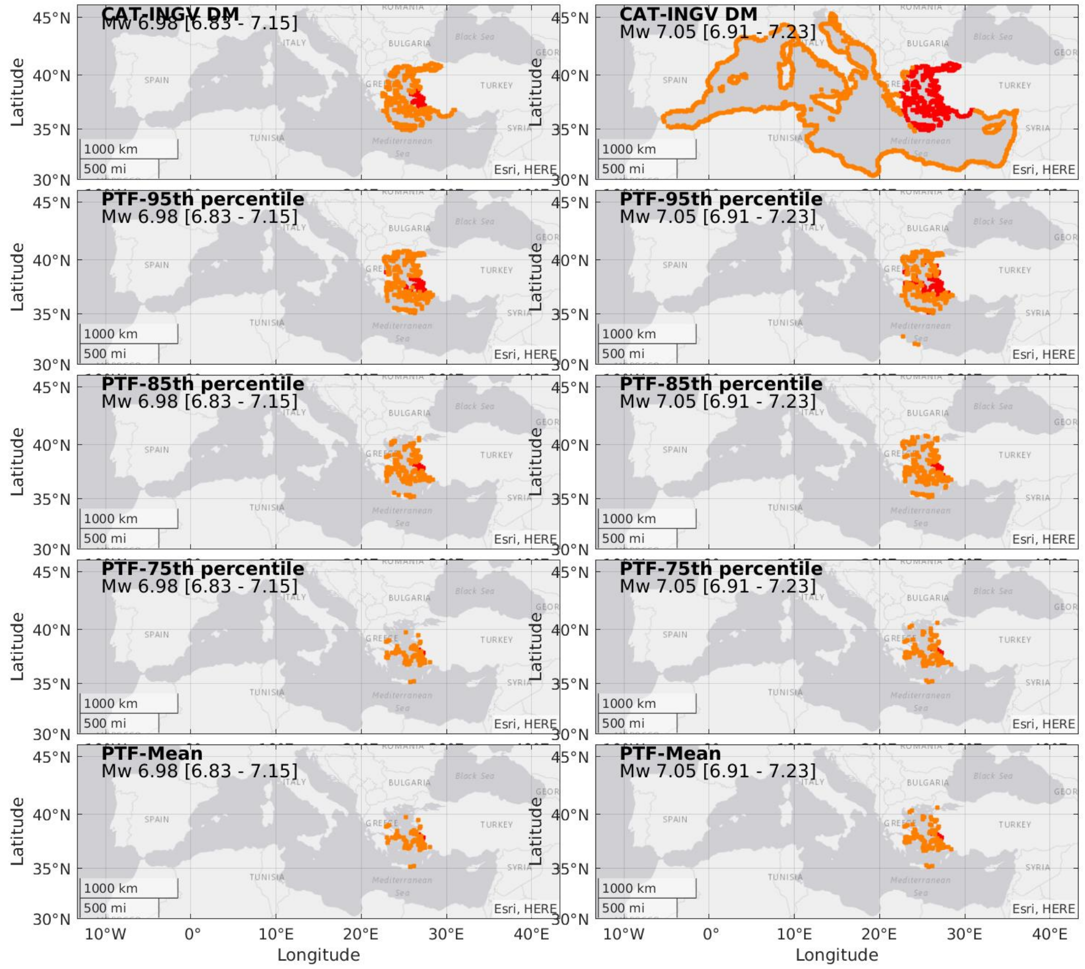

Supplementary Figure 7. DM and PTF alert levels for small perturbations to the magnitude.

DM and PTF alert level definition for the Samos-Izmir earthquakes, considering small perturbations to the magnitude estimation. Alert level based on DM (first row), different PTF percentiles (intermediate rows) and PTF mean (last row) are reported considering a shift of the magnitude distribution slightly below (left column) and above (right column) the magnitude threshold of $M_w = 7.0$.

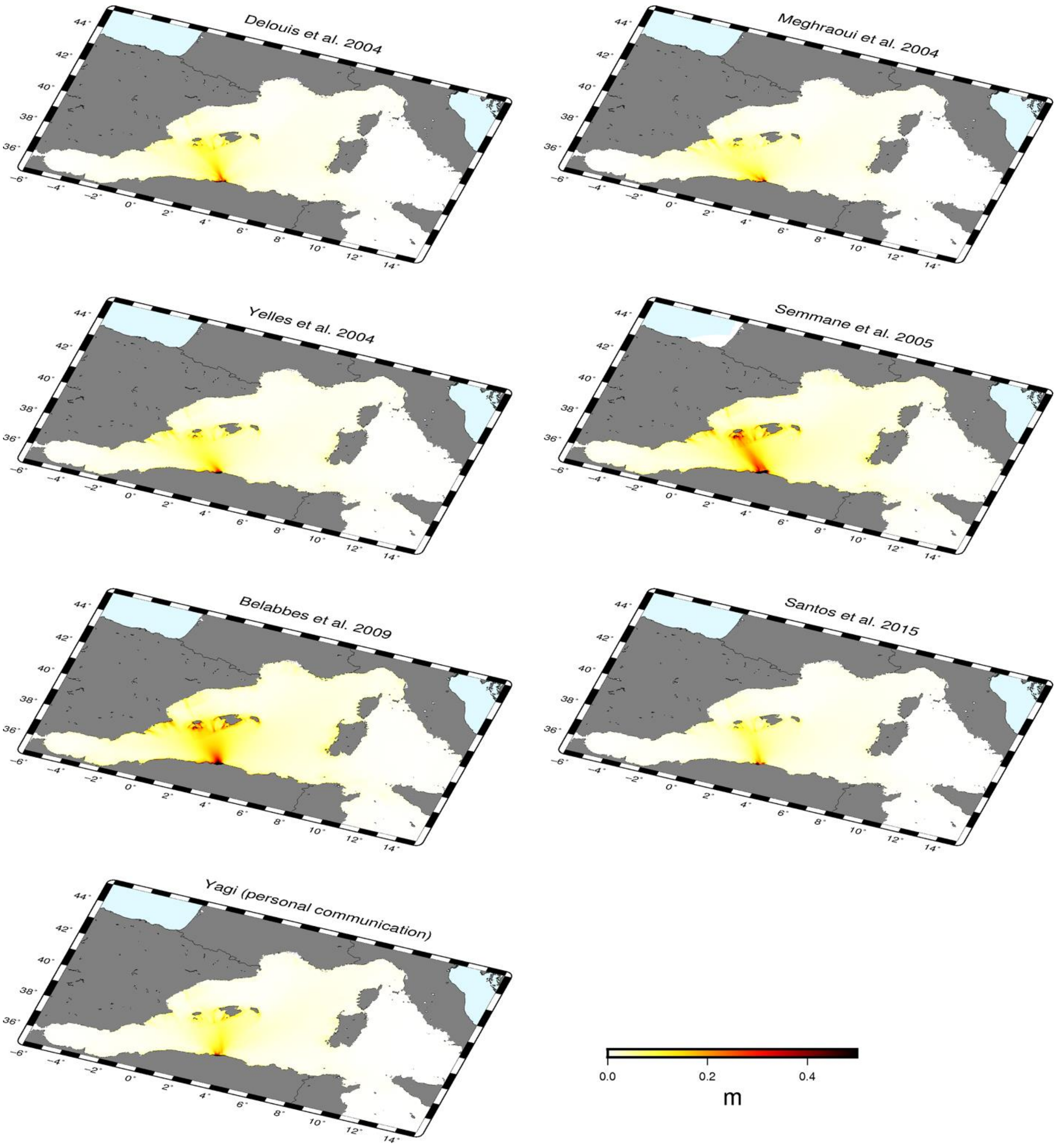

Supplementary Figure 8. Modelled wave amplitude for the 2003 Zemmouri-Boumerdes tsunami.

Map of the maximum wave amplitude from numerical tsunami simulations for all of the finite fault models of the Zemmouri-Boumerdes earthquake present in this study.

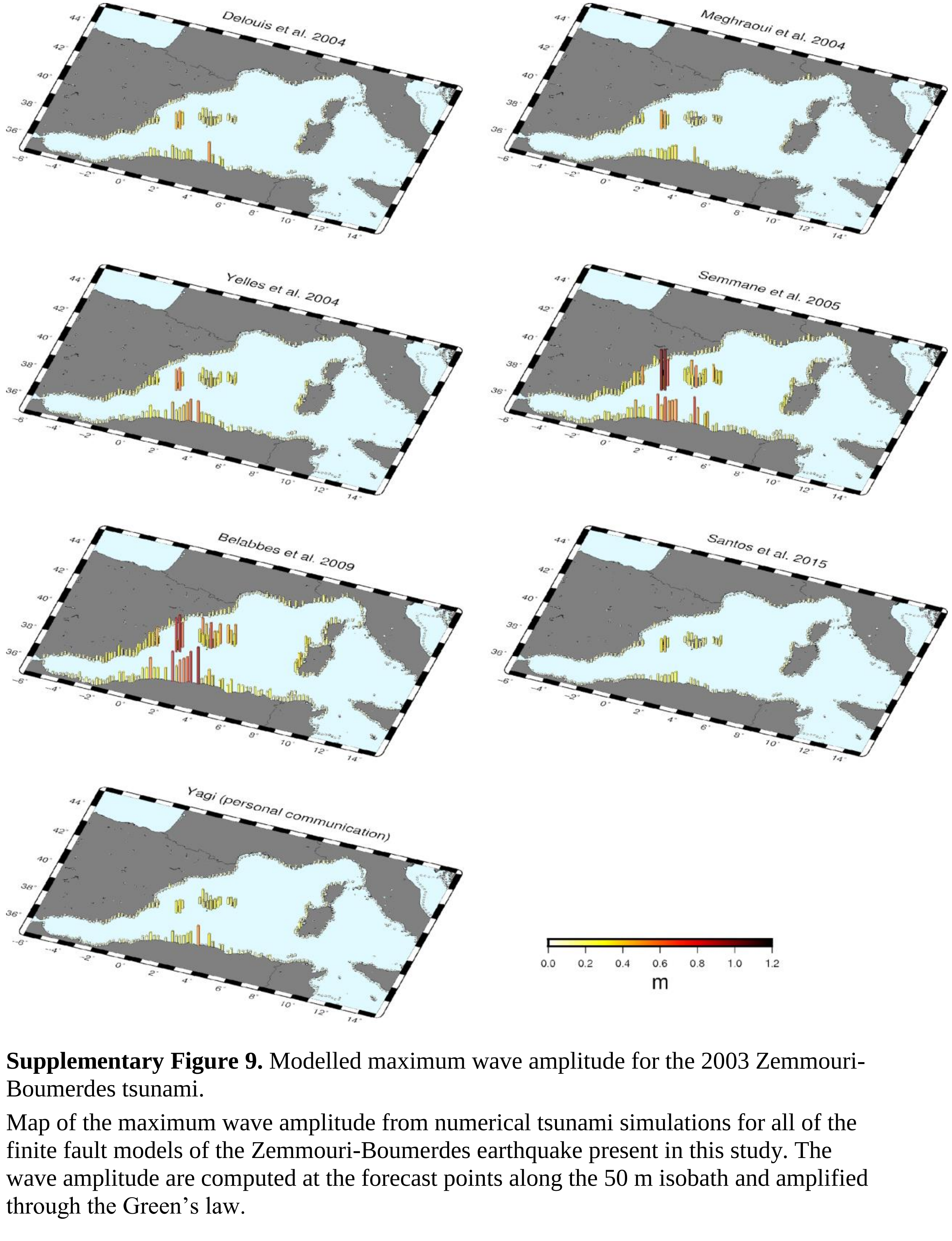

Supplementary Figure 9. Modelled maximum wave amplitude for the 2003 Zemmouri-Boumerdes tsunami.

Map of the maximum wave amplitude from numerical tsunami simulations for all of the finite fault models of the Zemmouri-Boumerdes earthquake present in this study. The wave amplitude are computed at the forecast points along the 50 m isobath and amplified through the Green's law.

Supplementary Tables

Acronym	***Description***
BMS	Best-Matching Scenario
BS	Background Seismicity
CAT-INGV	Centro Allerta Tsunami - Istituto Nazionale di Geofisica e Vulcanologia
DM	Decision Matrix
DPC	Dipartimento della Protezione Civile (Italian Civil Protection)
EE	Early-Est software
ENV	Envelop method
IOC/UNESCO	Intergovernative Oceanografic Commission of UNESCO
ISPRA	Istituto Superiore per la Protezione e la Ricerca Ambientale
NEAM	North-eastern Atlantic, the Mediterranean and connected seas
NEAMTWS	NEAM Tsunami Warning System (http://www.ioc-tsunami.org/)
NEAMTHM18	NEAM Tsunami Hazard Model 2018 (http://www.tsumaps-neam.eu/)
PS	Predominant Seismicity
PTHA	Probabilistic Tsunami Hazard Analysis
PTF	Probabilistic Tsunami Forecasting
TSP	Tsunami Service Provider
TEWS	Tsunami Early Warning System

Supplementary Table 1. Acronyms and abbreviations.
List of acronyms and abbreviations.

	Origin Time	EE version	Lat Lon Depth	Location Covariant Matrix	Mwp
				XX XY XZ YY YZ ZZ	p16 p50 p84
2003/05/21 M6.8 Boumerdes	2003.05.21T18:44:21.38	2	36.826 3.643 9.8	12.439 -0.867 -0.579 15.943 -0.245 12.045	6.86 6.91 7.01
		5	***36.868 3.652 9.8***	***12.510 1.225 -0.500 16.847 0.598 11.099***	***6.75 6.87 6.98***
		8	36.882 3.661 9.8	13.558 0.791 -0.362 15.479 1.362 11.362	6.73 6.81 6.90
2015/04/16 M6.4 Kasos Is.	2015.04.16T18:07:44.51	2	34.956 26.714 9.8	13.475 4.315 -0.279 28.745 0.956 6.881	6.03 6.25 6.36
		**5**	**35.163 26.745 19.5**	**10.959 3.538 1.224 19.924 2.467 11.587**	**6.17 6.30 6.47**
		8	35.149 26.763 19.5	10.684 3.385 1.082 20.314 2.318 11.303	6.11 6.23 6.39
2015/11/17 M6.5 Lefkas Is.	2015.11.17T07:10:10.28	2	38.805 20.505 19.5	1.385 0.317 -0.289 2.707 YZ -0.159 9.571	6.37 6.58 6.73
		**5**	**38.804 20.511 19.7**	**2.392 0.523 -0.184 4.693 0.348 3.446**	**6.36 6.53 6.72**
		8	38.739 20.509 12.9	1.477 0.380 -0.543 2.767 1.297 9.415	6.29 6.44 6.61
2016/01/25 M6.5 Gibraltar	2016.01.25T04:22:01.31	2	35.599 -3.700 19.5	15.582 -6.599 0.587 17.299 0.593 10.7516	6.27 6.44 6.59
		**5**	**35.448 -3.731 9.8**	**9.315 -2.044 -0.388 21.677 0.888 11.918**	**6.28 6.39 6.54**
		8	35.532 -3.731 9.8	11.174 -0.976 -0.353 20.279 1.078 10.915	6.27 6.40 6.54
2016/10/30 M6.7 Norcia	2016.10.30T06:40:18.93	2	42.863 13.158 10.1	1.209 0.393 -0.011 1.522 -0.030 0.304	6.58 6.72 6.90
		**5**	**42.869 13.151 10.1**	**1.406 0.410 -0.005 1.874 -0.036 0.233**	**6.54 6.72 6.84**
		8	42.858 13.143 10.1	1.474 0.410 -0.002 1.967 0.005 0.219	6.49 6.62 6.82
2017/06/12 M6.5 Lesvos Is.	2017.06.12T12:28:39.5	2	38.763 26.358 19.5	1.972 0.472 -0.006 1.873 0.004 9.687	6.09 6.42 6.68
		**5**	**38.848 26.376 19.5**	**2.627 0.811 -0.956 1.827 -0.467 10.009**	**6.24 6.51 6.69**
		8	38.842 26.339 10.0	3.019 1.332 -0.0283 3.732 -0.120 3.912	6.29 6.43 6.63
2017/07/20 M6.8 Kos-Bodrum	2017.07.20T22:31:12.27	2	36.752 27.448 19.5	11.832 2.331 -0.528 15.998 -0.360 11.769	6.47 6.71 6.88
		**5**	**36.918 27.444 9.8**	**11.190 2.297 -0.820 17.475 0.427 11.728**	**6.70 6.86 7.07**
		8	36.925 27.453 9.8	11.453 2.928 -0.812 17.635 0.256 11.961	6.63 6.78 7.03
2018/10/25 M6.8 Zakynthos	2018.10.25T22:54:50.69	2	37.343 20.522 19.5	1.564 0.644 -0.064 2.623 0.027 9.880	6.67 6.84 7.16
		**5**	**37.496 20.608 10.0**	**1.708 0.270 0.099 2.750 0.486 8.041**	**6.71 6.85 7.06**
		8	37.485 20.621 10.0	2.933 0.628 -0.076 4.024 0.232 3.293	6.64 6.83 7.07
2019/03/20 M6.0 Turkey	2019.03.20T06:34:29.64	2	37.424 29.492 9.8	12.137 2.073 0.118 16.577 0.265 11.248	5.52 5.59 5.98
		**5**	**37.438 29.500 9.8**	**12.211 2.884 0.027 16.499 0.861 12.09**	**5.56 5.73 5.92**
		8	37.410 29.518 15.2	13.248 3.978 -0.989 15.048 1.009 12.167	5.57 5.72 5.91
2019/09/21 M5.9 Albania	2019.09.21T14:04:25.67	2	41.314 19.426 10.0	2.060 0.246 0.082 2.334 -0.12 8.684	5.58 5.81 6.03
		**5**	**41.317 19.475 10.0**	**2.383 0.118 -0.116 2.789 0.065 6.639**	**5.65 5.81 5.99**
		8	41.323 19.482 19.5	1.913 0.039 -0.703 2.144 0.438 9.626	5.63 5.81 5.95
2019/11/26 M6.5 Albania	2019.11.26T02:54:12.53	2	41.377 19.426 10.0	3.576 0.749 -0.340 2.339 0.576 8.462	6.29 6.53 6.62
		**5**	**41.365 19.541 19.5**	**1.726 0.045 -0.266 2.195 0.470 10.075**	**6.36 6.54 6.72**
		8	41.379 19.521 19.5	2.060 0.132 -0.505 2.281 0.509 8.589	6.35 6.54 6.70
2020/05/02 M6.7 Ierapetra	2020.05.02T12:51:05.9	2	34.052 25.690 19.5	7.778 3.866 0.141 28.428 0.858 11.705	6.27 6.47 6.75
		**5**	**34.288 25.739 9.8**	**10.846 1.224 0.403 20.290 0.717 11.185**	**6.50 6.68 6.77**
		8	34.260 25.721 9.8	11.822 2.567 0.048 19.888 0.820 11.047	6.49 6.66 6.75
2020/10/30 M7.0 Samos-Izmir	2020-10-30T11:51:26.1	2	37.839 26.837 9.8	13.636 3.427 0.147 14.539 0.782 12.124	6.91 7.05 7.23
		**5**	**37.839 26.829 9.8**	**13.948 4.844 0.278 18.112 0.179 10.253**	**6.83 6.98 7.15**
		8	37.839 26.829 9.8	14.170 4.938 -0.042 18.557 0.078 10.344	6.82 6.94 7.14
NEAMWave	-	-	37.50 21.00 12.0	100. 0. 0. 100. 0. 100.	8.30 8.50 8.70
2010/02/27 M8.8 Maule	2010.02.27-06:34:10.39	-	-36.122 -72.898 30.5	100. 0. 0. 100. 0. 100.	8.60 8.80 9.00

Supplementary Table 2. Early-Est (EE) location and magnitude parameters.

Location and magnitude parameters in the Mediterranean area retrospectively applying the Early-Est (EE) software. From left to right: origin time, latitude, longitude [degree] and depth [km] of the epicentre, 6 elements of the location covariant matrix [km^2], Magnitude M_{wp} (16th percentile, median and 84th percentile). EE version 5 (in bold) is taken as a reference.

Source	Plane1[a]			Plane2		
	Strike (deg)	Dip (deg)	Rake (deg)	Strike (deg)	Dip (deg)	Rake (deg)
Global CMT (https://www.globalcmt.org/)	57	44	71	262	49	107
USGS (https://earthquake.usgs.gov/data/comcat/)	54	47	88	237	43	92
INGV (http://cnt.rm.ingv.it/)	65	27	86	250	63	92
Braunmiller and Bernardi 2005	62	25	82	251	65	94
CNRS (http://wphase.unistra.fr/)	75	30	98	246	61	85
Yelles et al. 2004	55	43	84	-	-	-
Semmane et al. 2005	54	47	90	-	-	-
Meghraoui et al. 2004	54	50	90	-	-	-
Santos&al.2015	64	40	91	-	-	-
Belabbès et al. 2009	65	35	90	-	-	-
Delouis et al. 2004	70	45	99	-	-	-
Yagi pers. comm.	75	40	126	-	-	-

[a] *Plane 1 is the principal fault according to field observations*.

Supplementary Table 3. 2003 Zemmouri-Boumerdes fault geometry and mechanisms. Fault geometry and mechanism for 5 seismic moment tensors and 7 finite fault models (see Supplementary Note 4) inversions.

	**1.5 STD**		**2.0 STD**		**2.5 STD**		**3.0 STD**	
	**Scenarios**	**Comp. Time**	**Scenarios**	**Comp. Time**	**Scenarios**	**Comp. Time**	**Scenarios**	**Comp. Time**
NEAMWave2017 M8.5 exercise	8964 PS 0 BS	~ 30’’	**14676PS 0 BS**	**~ 30’’**	19410 PS 0 BS	~45’’	24136 PS 104688 BS	> 120’’
2003/05/21 M6.8 Boumerdes	0 PS 4320 BS	< 15’’	**0 PS 15408 BS**	**~ 30’’**	0 PS 29520 BS	~45’’	0 PS 49824 BS	~ 75’’
2015/04/16 M6.4 Kasos Is.	0 PS 1872 BS	< 15’’	**0 PS 4320 BS**	**~15’’**	0 PS 22032 BS	~30’’	0 PS 37152 BS	~60’’
2015/11/17 M6.5 Lefkas Is.	0 PS 3312 BS	< 15’’	**0 PS 13680 BS**	**~30’’**	0 PS 29952 BS	~45’’	0 PS 116928 BS	>120’’
2016/01/25 M6.5 Gibraltar	0 PS 2304 BS	< 15’’	**0 PS 4032 BS**	**< 15’’**	0 PS 8064 BS	~15’’	0 PS 14256 BS	~30’’
2016/10/30 M6.7 Norcia	0 PS 7056 BS	< 15”	**0 PS 18144 BS**	**~30’’**	0 PS 51408 BS	~ 75’’	0 PS 91008 BS	~120’’
2017/06/12 M6.5 Lesvos Is.	0 PS 5472 BS	< 15”	**0 PS 19152 BS**	**~30’’**	0 PS 78768 BS	~120’’	0 PS 172368 BS	>120’’
2017/07/20 M6.8 Kos-Bodrum	0 PS 11952 BS	~ 30”	**0 PS 48096 BS**	**~75’’**	0 PS 116784 BS	> 120’’	0 PS 227952 BS	> 120’’
2018/10/25 M6.8 Zakynthos	924 PS 8640 BS	~30”	**1576 PS 19008 BS**	**~30’’**	3884 PS 65088 BS	~ 110’’	6256 PS 144720 BS	> 120’’
2019/03/20 M6.0 Turkey	0 PS 0 BS	-	**0 PS 4608 BS**	**< 15’’**	0 PS 9216 BS	~15’’	0 PS 18576 BS	30’’
2019/09/21 M5.9 Albania	0 PS 0 BS	-	**0 PS 5184 BS**	**< 15’’**	0 PS 6912 BS	< 15’’	0 PS 6912 BS	< 15”
2019/11/26 M6.5 Albania	0 PS 7056 BS	~ 30”	**0 PS 18144 BS**	**~30’’**	0 PS 34992 BS	~ 45’’	0 PS 90720 BS	~ 105’’
2020/05/02 M6.7 Ierapetra	832 PS 0 BS	< 15”	**1652 PS 14832 BS**	**~30’’**	2848 PS 26352 BS	~ 60’’	5190 PS 58320 BS	~ 90’’
2020/10/30 M7.0 Samos-Izmir	0 PS 16128 BS	~ 25’’	0 PS 38736 BS	~ 50’’	0 PS 95472 BS	~ 110’’	0 PS 277632 BS	> 120’’
2010/02/27 M8.8 Maule (Chile)	-	-	**13380 PS 0 BS**	**~30’’**	-	-	-	-

Supplementary Table 4. Ensemble members and computational times.

Ensemble members and computational times for different cutoffs. A cutoff of 2 STD is taken as reference. Computational times must be considered as an upper limit, being obtained with a serial non-engineered MATLAB code (MATLAB ver. R2019b, The MathWorks Inc., Natick, Massachusetts, 2019) with preloaded basic information, running in an HP ProLiant DL580 GEN9 four 14-core Intel(R) Xeon(R) with E7-4830 CPUs clocked at 2.0 GHz (56 total compute cores and 3072 GB RAM).

	**Global-CMT**						**Quick Regional-CMT**					
	**Plane 1**			**Plane 2**			**Plane 1**			**Plane 2**		
	Strike (deg)	Dip (deg)	Rake (deg)	Strike (deg)	Dip (deg)	Rake (deg)	Strike (deg)	Dip (deg)	Rake (deg)	Strike (deg)	Dip (deg)	Rake (deg)
2003/05/21 M6.8 Boumerdes	57	44	71	262	49	107	64	27	86	250	63	92
2015/04/16 M6.4 Kasos Is.	56	43	21	310	76	131	51	51	26	304	70	138
2015/11/17 M6.5 Lefkas Is.	22	64	179	113	89	26	23	71	179	113	89	19
2016/01/25 M6.5 Gibraltar	120	73	166	214	76	17	211	75	10	119	80	165
2016/10/30 M6.7 Norcia	154	37	-96	342	53	-85	155	37	-98	345	53	-84
2017/06/12 M6.5 Lesvos Is.	286	43	-93	110	47	-87	84	33	-131	311	66	-67
2017/07/20 M6.8 Kos-Bodrum	278	36	-82	88	55	-96	296	49	-55	68	52	-124
2018/10/25 M6.8 Zakynthos	11	28	165	114	83	63	17	27	168	117	85	63
2019/03/20 M6.0 Turkey	321	42	-87	137	48	-93	330	34	-79	136	57	-98
2019/09/21 M5.9 Albania	336	31	112	130	62	77	328	37	95	142	53	86
2019/11/26 M6.5 Albania	351	25	114	145	68	79	351	22	115	145	71	80
2020/05/02 M6.7 Ierapetra (*)	257[a]	24[a]	71[a]	97[a]	68[a]	98[a]	273	26	94	89	64	88
2020/10/30 M7.0 Samos-Izmir (*)	270	37	-95	96	53	-86	289	40	-69	82	53	-107

[*] *from Quick CMT catalogue*

Supplementary Table 5. Fault geometry and mechanism for all the events in the testing dataset.

Fault geometry and mechanism estimations for the all the events in the testing dataset used for testing the PTF source model in hind-casting mode (Fig. 4a).

Event	Stations' codes per event	Provider
2015/04/16 M6.4 Kasos Is.	bodru	KOERI
	kast, gvd9	IOC
2015/11/17 M6.5 Lefkas Is.	CR08, kaps, kata, koro, OT15, TA18	IOC
2016/01/25 M6.5 Gibraltar	alge, alme, carb, carg, gibr2, mal3, meli, motr	IOC
2016/10/30 M6.7 Norcia	ajac2, AN15, cent2, CI20, GA37, LI11, MC41, OR24, PL14, RA10, SB36, sole2, TR22, VE19, VI12	IOC
2017/06/12 M6.5 Lesvos Is.	bodru, gokce	KOERI
	NOA03, NOA05, NOA06	TAD
	peir, syro	IOC
2017/07/20 M6.8 Kos-Bodrum	bodru, gokce	KOERI
	NOA03, NOA04	TAD
	peir, syro	IOC
2018/10/25 M6.8 Zakynthos	CR08, kala, kata, lcst	IOC
	NOA05, NOA06, NOA08	TAD
	OT15, peir, syro	IOC
2019/03/20 M6.0 Turkey	bozya	KOERI
2019/09/21 M5.9 Albania	BA05, CR08, IT45, OT15, PL14, TA18, VI12	IOC
	NOA12	TAD
2019/11/26 M6.5 Albania	BA05, CR08, IT45, OT15, PL14, TA18, VI12	IOC
	NOA12	TAD
2020/05/02 M6.7 Ierapetra	bodru	KOERI
	feth, syro	IOC
	NOA03, NOA04	TAD
2020/10/30 M7.0 Samos-Izmir	bodru, gokce, marma	KOERI
	NOA03, NOA04, NOA10	TAD
	kos1, kos2, plom, syro	IOC

IOC (Intergovernmental Oceanographic Commission of UNESCO): http://www.ioc-sealevelmonitoring.org/list.php
TAD (Tsunami Alert Device) Server: https://webcritech.jrc.ec.europa.eu/TAD_server/Home?group=NOA
KOERI http://sea.koeri.boun.edu.tr/worldSealevelinterface/?list=true&orderby=name

Supplementary Table 6. Tide-gauge stations.

Tide-gauge stations used for each event for testing purposes (Supplementary Figure 3). Data have been automatically searched in a radius within 400 km from the epicentre. More information about tide-gauge stations and managing institutions are reported in Supplementary Data 3.

	G-CMT Double-Couple	***G-CMT Fault Plane***	***QRCMT Double-Couple***	***QRCMT Fault Plane***
All (13 events)	0.13	0.35	0.19	0.42
All, since October 2016 (9 events)	0.27	0.55	0.09	0.28
2003/05/21 M6.8 Boumerdes	0.08	0.11	0.74	1
2015/04/16 M6.4 Kasos Is.	0.10	0.21	0.23	0.21
2015/11/17 M6.5 Lefkas Is.	0.69	0.71	0.69	0.71
2016/01/25 M6.5 Gibraltar	0.65	0.35	0.65	0.35
2016/10/30 M6.7 Norcia	0.41	0.41	0.42	0.42
2017/06/12 M6.5 Lesvos Is.	0.30	0.47	0.13	0.32
2017/07/20 M6.8 Kos-Bodrum	0.40	0.14	0.66	0.46
2018/10/25 M6.8 Zakynthos	0.07	0.22	0.07	0.22
2019/03/20 M6.0 Turkey	0.66	1	0.09	0.09
2019/09/21 M5.9 Albania	0.47	1	0.64	1
2019/11/26 M6.5 Albania	0.58	1	0.58	1
2020/05/03 M6.7 Ierapetra	0.54[a]	0.45[a]	0.85	0.95
2020/10/30 M7.0 Samos-Izmir	0.51a	0.21a	0.22	0.09

[a] *earthquake parameters from Quick global CMT catalogue*

Supplementary Table 7. P-Value of tests on strike, dip, and rake angles.

The null hypothesis would be rejected for P-Value < 0.05 or 0.01. In our tests, the null hypothesis is never rejected. The tests have been performed both individually for each event and simultaneously for all the events (see Methods), comparing PTF input to Global CMT (G-CMT, https://www.globalcmt.org/, [35,36]) and Quick Regional-CMT (QRCMT http://autorcmt.bo.ingv.it/quicks.html, [37]) catalogues for both preferred fault plane and double couples. To assure complete independence between the inference model and testing data, we repeated the test with the 8 events occurring after September 2016, obtaining equivalent results.

**Depth**	**Epicenter Location**	**M**	**Tsunami Potential**	**Type of Bulletin**		
<100km	Offshore or close the coast (≦40 km inland)	5.5≦M≦6.0	Nil	**Information Bulletin**	**Information Bulletin**	**Information Bulletin**
		6.0<M≦6.5	Weak potential of local tsunami	**Local Tsunami Advisory**	**Information Bulletin**	**Information Bulletin**
	Inland (>40km and ≦100km	5.5≦M≦6.5	Nil	**Information Bulletin**	**Information Bulletin**	**Information Bulletin**
	Offshore or close the coast (≦100 km inland)	6.5<M≦7.0	Potential of destructive local tsunami <100km	**Local Tsunami Watch**	**Regional Tsunami Advisory**	**Information Bulletin**
		7.0<M≦7.5	Potential of destructive regional tsunami <400km	**Local Tsunami Watch**	**Regional Tsunami Watch**	**Basin-wide Tsunami Advisory**
		M>7.5	Potential of destructive tsunami in the whole basin >400km	**Local Tsunami Watch**	**Regional Tsunami Watch**	**Basin-wide Tsunami Watch**
≧100km	Offshore or close to the coast (≦100 km inland)	M≧5.5	Nil	**Information Bulletin**	**Information Bulletin**	**Information Bulletin**
				Local≦100 km	100≦Regional<400	Basin-wide ≧400

Supplementary Table 8. CAT-INGV NEAMTWS Decision Matrix (DM). CAT-INGV NEAMTWS Decision Matrix (DM) for the Mediterranean Sea.

	**DM**	**ENV**	**PTF-p99**	**PTF-p95**	**PTF-p90**	**PTF-p85**	**PTF-p80**	**PTF-p70**	**PTF-p60**	**PTF-Med**	**PTF-Mean**	**BMS**
**Proportion of correct alert-levels**	***0.42***	***0.64***	***0.47***	***0.67***	***0.76***	***0.84***	***0.87***	***0.86***	***0.86***	***0.84***	***0.86***	***0.86***
**Proportion of false-alarms**	***0.55***	***0.34***	***0.52***	***0.32***	***0.21***	***0.12***	***0.07***	***0.03***	***0.02***	***<0.01***	***0.05***	***0.02***
**Proportion of missed-alarms**	***0.03***	***0.02***	***< 0.01***	***0.02***	***0.03***	***0.04***	***0.06***	***0.11***	***0.12***	***0.15***	***0.09***	***0.12***

Supplementary Table 9. Correct/missed/false alarms for DM, ENV, BMS and PDF.

Observed percentages of correct/missed/false alarms, adopting DM, ENV, BMS and PDF-based definition of alert levels

Supplementary References

1. Lomax, A., Michelini, A. & Curtis, A. Earthquake Location, Direct, Global-Search Methods. in *Encyclopedia of Complexity and Systems Science* (ed. Meyers, R. A.) 2449–2473 (Springer, 2009). doi:10.1007/978-0-387-30440-3_150.
2. Lomax, A. & Michelini, A. Mwpd: A duration–amplitude procedure for rapid determination of earthquake magnitude and tsunamigenic potential from P waveforms. *Geophys J Int* **176**, 200–214 (2009).
3. Bernardi, F. *et al.* Appraising the Early-est earthquake monitoring system for tsunami alerting at the Italian Candidate Tsunami Service Provider. *Natural Hazards and Earth System Sciences* **15**, 2019–2036 (2015).
4. Chock, G., Yu, G., Thio, H. K. & Lynett, P. J. Target Structural Reliability Analysis for Tsunami Hydrodynamic Loads of the ASCE 7 Standard. *Journal of Structural Engineering* **142**, 04016092 (2016).
5. Belabbès, S., Wicks, C., Çakir, Z. & Meghraoui, M. Rupture parameters of the 2003 Zemmouri (Mw 6.8), Algeria, earthquake from joint inversion of interferometric synthetic aperture radar, coastal uplift, and GPS. *Journal of Geophysical Research: Solid Earth* **114**, B03406 (2009).
6. Delouis, B. *et al.* Slip distribution of the 2003 Boumerdes-Zemmouri earthquake, Algeria, from teleseismic, GPS, and coastal uplift data. *Geophysical Research Letters* **31**, L18607 (2004).
7. Meghraoui, M. *et al.* Coastal uplift and thrust faulting associated with the Mw = 6.8 Zemmouri (Algeria) earthquake of 21 May, 2003. *Geophysical Research Letters* **31**, L19605 (2004).

8. Santos, R., Caldeira, B., Bezzeghoud, M. & Borges, J. F. The Rupture Process and Location of the 2003 Zemmouri–Boumerdes Earthquake (Mw 6.8) Inferred from Seismic and Geodetic Data. *Pure Appl. Geophys.* **172**, 2421–2434 (2015).
9. Semmane, F., Campillo, M. & Cotton, F. Fault location and source process of the Boumerdes, Algeria, earthquake inferred from geodetic and strong motion data. *Geophysical Research Letters* **32**, L01305 (2005).
10. Yagi, Y. & Fukahata, Y. Introduction of uncertainty of Green's function into waveform inversion for seismic source processes. *Geophysical Journal International* **186**, 711–720 (2011).
11. Yelles, K., Lammali, K., Mahsas, A., Calais, E. & Briole, P. Coseismic deformation of the May 21st, 2003, Mw = 6.8 Boumerdes earthquake, Algeria, from GPS measurements. *Geophysical Research Letters* **31**, L13610 (2004).
12. Okada, Y. Surface deformation due to shear and tensile faults in a half-space. *Bulletin of the Seismological Society of America* **75**, 1135–1154 (1985).
13. de la Asunción, M. *et al.* Efficient GPU implementation of a two waves TVD-WAF method for the two-dimensional one layer shallow water system on structured meshes. *Computers & Fluids* **80**, 441–452 (2013).
14. IOC/UNESCO. *[Intergovernmental Oceanographic Commission / UNESCO] NEAMWave17 – A Tsunami Warning and Communication Exercise for the North-eastern Atlantic, the Mediterranean, and Connected Seas Region, 31 October – 3 November 2017: Exercise Instructions*. (2017).

15. Strasser, F. O., Arango, M. C. & Bommer, J. J. Scaling of the Source Dimensions of Interface and Intraslab Subduction-zone Earthquakes with Moment Magnitude. *Seismological Research Letters* **81**, 941–950 (2010).
16. Heidarzadeh, M. & Satake, K. The 21 May 2003 Tsunami in the Western Mediterranean Sea: Statistical and Wavelet Analyses. *Pure Appl. Geophys.* **170**, 1449–1462 (2013).
17. Power, W., Downes, G. & Stirling, M. Estimation of Tsunami Hazard in New Zealand due to South American Earthquakes. *Pure appl. geophys.* **164**, 547–564 (2007).
18. Dogan, G. G. *et al.* The 20th July 2017 Bodrum–Kos Tsunami Field Survey. *Pure Appl. Geophys.* **176**, 2925–2949 (2019).
19. Dogan, G. G. *et al.* The 30 October 2020 Aegean Sea Tsunami: Post-Event Field Survey Along Turkish Coast. *Pure Appl. Geophys.* **178**, 785–812 (2021).
20. Triantafyllou, I. *et al.* The Tsunami Caused by the 30 October 2020 Samos (Aegean Sea) Mw7.0 Earthquake: Hydrodynamic Features, Source Properties and Impact Assessment from Post-Event Field Survey and Video Records. *JMSE* **9**, 68 (2021).
21. Ekström, G., Nettles, M. & Dziewoński, A. M. The global CMT project 2004–2010: Centroid-moment tensors for 13,017 earthquakes. *Physics of the Earth and Planetary Interiors* **200–201**, 1–9 (2012).
22. Dziewonski, A. M., Chou, T.-A. & Woodhouse, J. H. Determination of earthquake source parameters from waveform data for studies of global and regional seismicity. *Journal of Geophysical Research: Solid Earth* **86**, 2825–2852 (1981).

23. Pondrelli, S. European-Mediterranean Regional Centroid-Moment Tensors Catalog (RCMT) [Data set]. Istituto Nazionale di Geofisica e Vulcanologia (INGV). https://doi.org/10.13127/rcmt/euromed (2002).
24. National Geophysical Data Center. Global Historical Tsunami Database. doi:10.7289/V5PN93H7.
25. NOAA. [National Oceanic and Atmospheric Administration] National Data Buoy Center. https://www.ndbc.noaa.gov/.
26. Melgar, D. *et al.* Source characteristics of the 2015 Mw6.5 Lefkada, Greece, strike-slip earthquake. *Journal of Geophysical Research: Solid Earth* **122**, 2260–2273 (2017).
27. Cirella, A. *et al.* The 2018 Mw 6.8 Zakynthos (Ionian Sea, Greece) earthquake: seismic source and local tsunami characterization. *Geophys J Int* **221**, 1043–1054 (2020).
