# Supplementary material for: Probabilistic tsunami forecasting for early warning
Source: Nat Commun. 2021 Sep 28;12:5677. doi: 10.1038/s41467-021-25815-w (PMC8479076; doi:10.1038/s41467-021-25815-w)
Supplement: Supplementary file 3 — Description of Additional Supplementary Files [file 41467_2021_25815_MOESM3_ESM.pdf]

Description of Additional Supplementary Files

File name: Supplementary Dataset 1
Description: Detailed information about Forecast Points. The detailed information about the Forecast Points in the Mediterranean used in this study, including ID, longitude, latitude, and depth, is reported in the file csv file.

File name: Supplementary Dataset 2
Description: Seismic stations used by Early-Est. For each station, we provided the code of the network code, the station code, latitude and longitude in degree and elevation in meters. For the relocation of the events, we selected only the stations within a radius of 50 degree from each epicentre.

File name: Supplementary Dataset 3
Description: Sealevel records. Sea-level records for the tidegauge stations grouped event by event as listed in Supplementary Table 6. Data are presented as twocolumn text files (.txt), containing time (in seconds from the earthquake origin time) and the sea level variation (in meters) together with a plot figure (.png), showing both the raw and the tide-removed data. Characteristics of each tidegauge station (i.e., station code, provider, country, location, institutions, latitude, longitude) are also provided (Sea_Level_Stations.docx). These data are used for testing tsunami forecasts and can be found in one zip file.

File name: Supplementary Dataset 4
Description: Automatic figures produced by PTF. Repository of the automatic figures produced by PTF for all the considered case studies of Supplementary Table 2. For each earthquake, we have one figure: (a) the real-time estimation of the source factor, as reported in the monitoring room in terms of the marginal distributions for each source parameter: magnitude and depth distributions (global and for PS/BS separately); slip distribution for PS; fault centre, strike, dip and rake distributions for BS. (b) the hazard maps for mean, median, 5th, and 95th percentiles. (c) the alert levels corresponding to the CAT-INGV DM, and PTF’s mean, median, 95th, 90th, 85th, 80th, 75th, 70th, 65th, 60th, and 55th percentiles.

File name: Supplementary Dataset 5
Description: Alert levels comparison figures. Repository of the alert levels comparison figures. All figures contain two panels: (a) the panel above contains the comparison between different alert levels at all forecast points with observations; (b) the middle panel contains the overall statistics of correct / missed / false alarms given by each model for all forecast points with observations; (c) the panel below contains the total number of alerted forecast points (watch or advisory).
