# Supplementary figures and images for: Probabilistic tsunami forecasting for early warning (part 1 of 2)
Source: Nat Commun. 2021 Sep 28;12:5677. doi: 10.1038/s41467-021-25815-w (PMC8479076; doi:10.1038/s41467-021-25815-w)

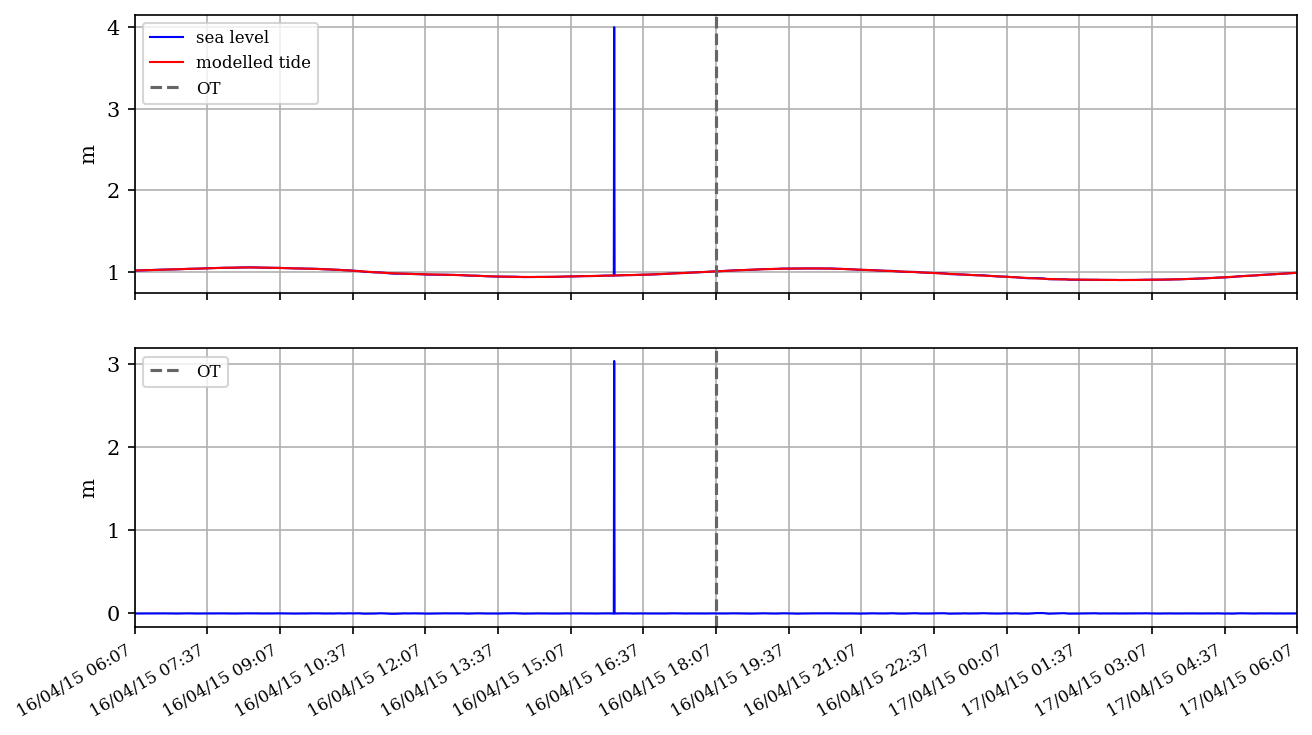

Supplement: Supplementary file 6 — Supplementary Dataset 3. Sea-level records. [file 41467_2021_25815_MOESM6_ESM.zip › sea_level_records/2015-04-16_Kasos/bodru.rad.rmn.png]

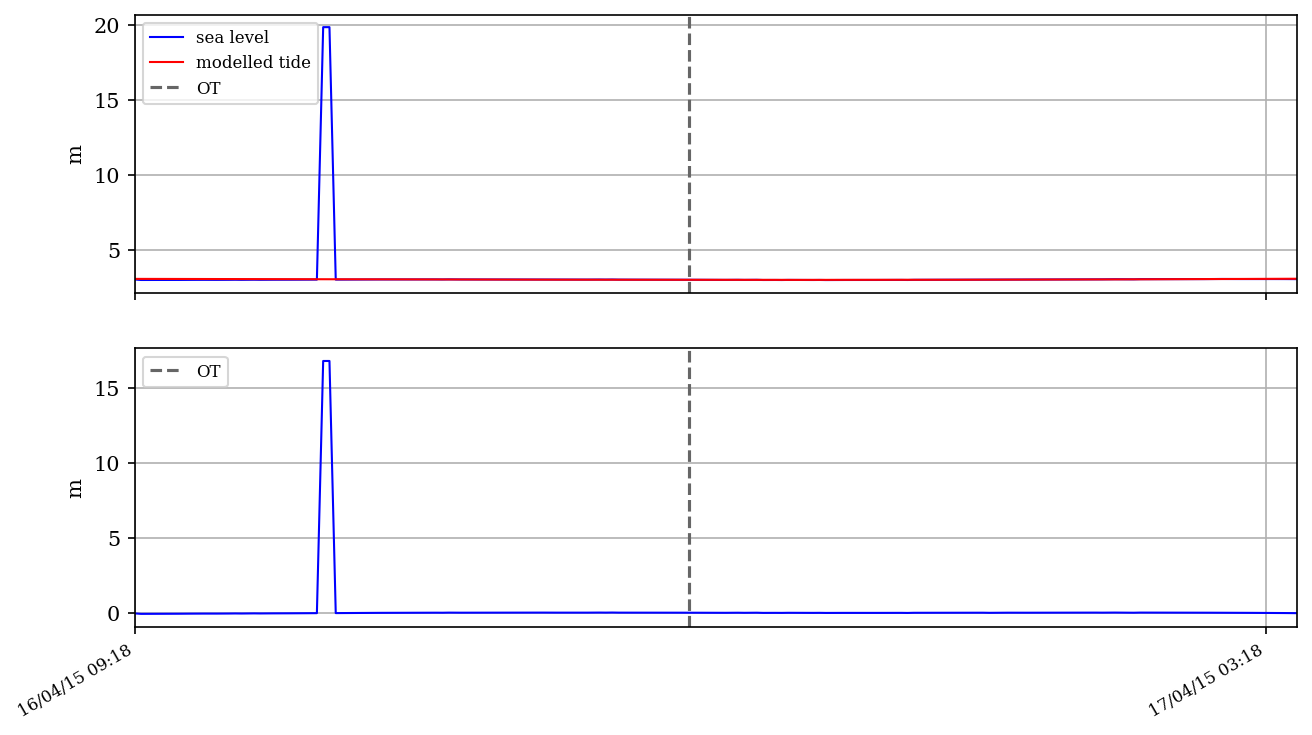

Supplement: Supplementary file 6 — Supplementary Dataset 3. Sea-level records. [file 41467_2021_25815_MOESM6_ESM.zip › sea_level_records/2015-04-16_Kasos/gvd9.aqu.rmn.png]

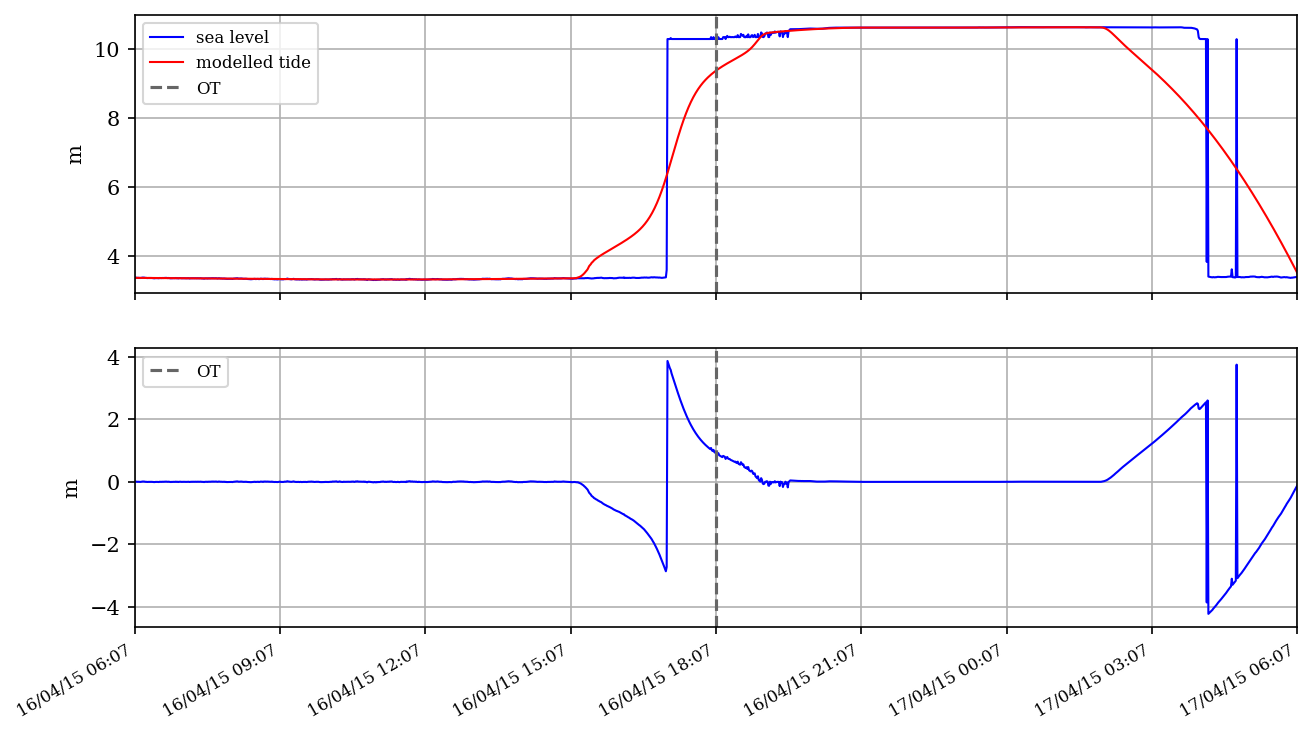

Supplement: Supplementary file 6 — Supplementary Dataset 3. Sea-level records. [file 41467_2021_25815_MOESM6_ESM.zip › sea_level_records/2015-04-16_Kasos/kast.rad.rmn.png]

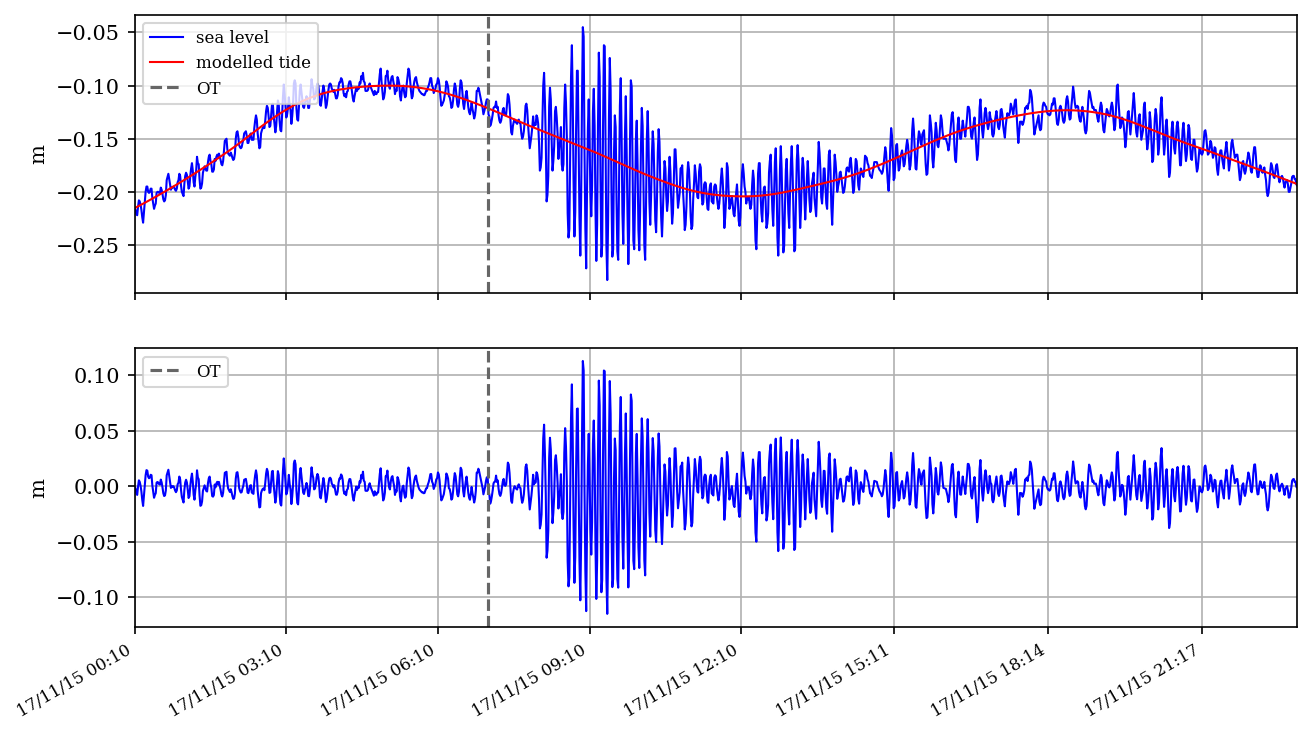

Supplement: Supplementary file 6 — Supplementary Dataset 3. Sea-level records. [file 41467_2021_25815_MOESM6_ESM.zip › sea_level_records/2015-11-17_Lefkas/CR08.rad.rmn.png]

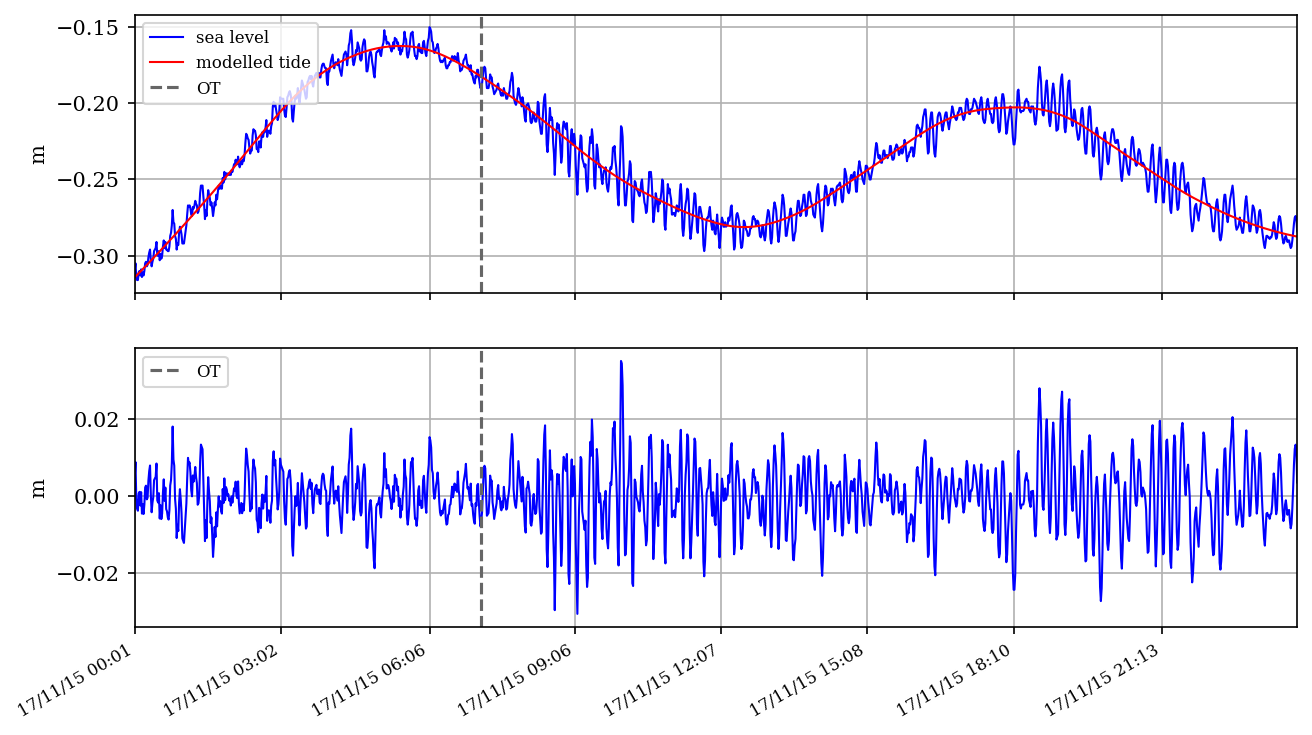

Supplement: Supplementary file 6 — Supplementary Dataset 3. Sea-level records. [file 41467_2021_25815_MOESM6_ESM.zip › sea_level_records/2015-11-17_Lefkas/OT15.rad.rmn.png]

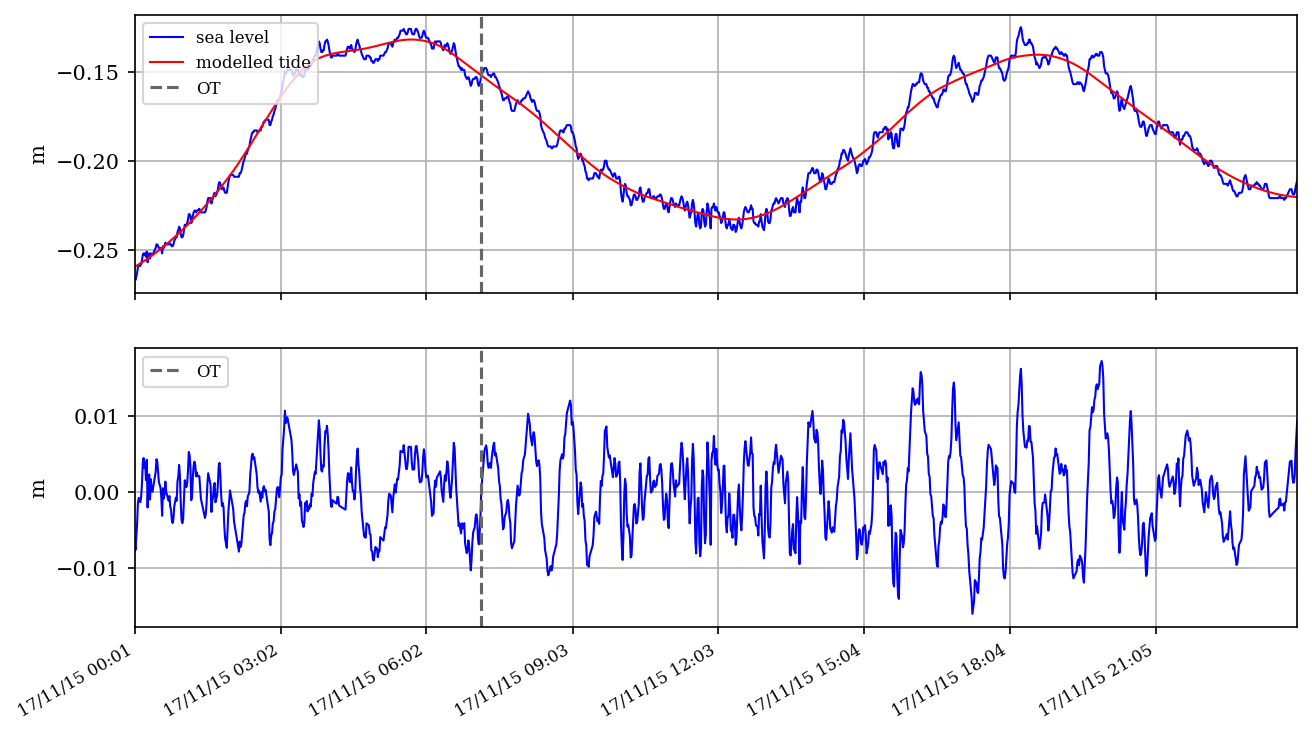

Supplement: Supplementary file 6 — Supplementary Dataset 3. Sea-level records. [file 41467_2021_25815_MOESM6_ESM.zip › sea_level_records/2015-11-17_Lefkas/TA18.rad.rmn.png]

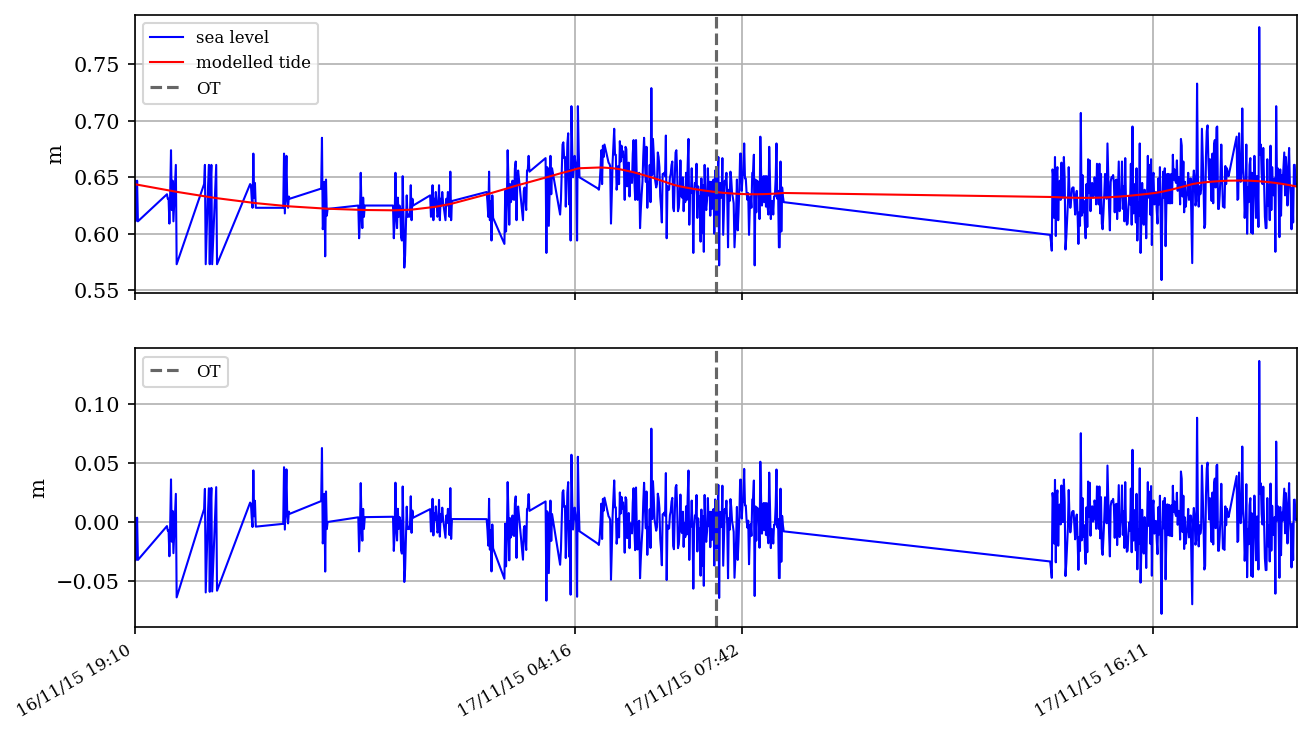

Supplement: Supplementary file 6 — Supplementary Dataset 3. Sea-level records. [file 41467_2021_25815_MOESM6_ESM.zip › sea_level_records/2015-11-17_Lefkas/kaps.rad.rmn.png]

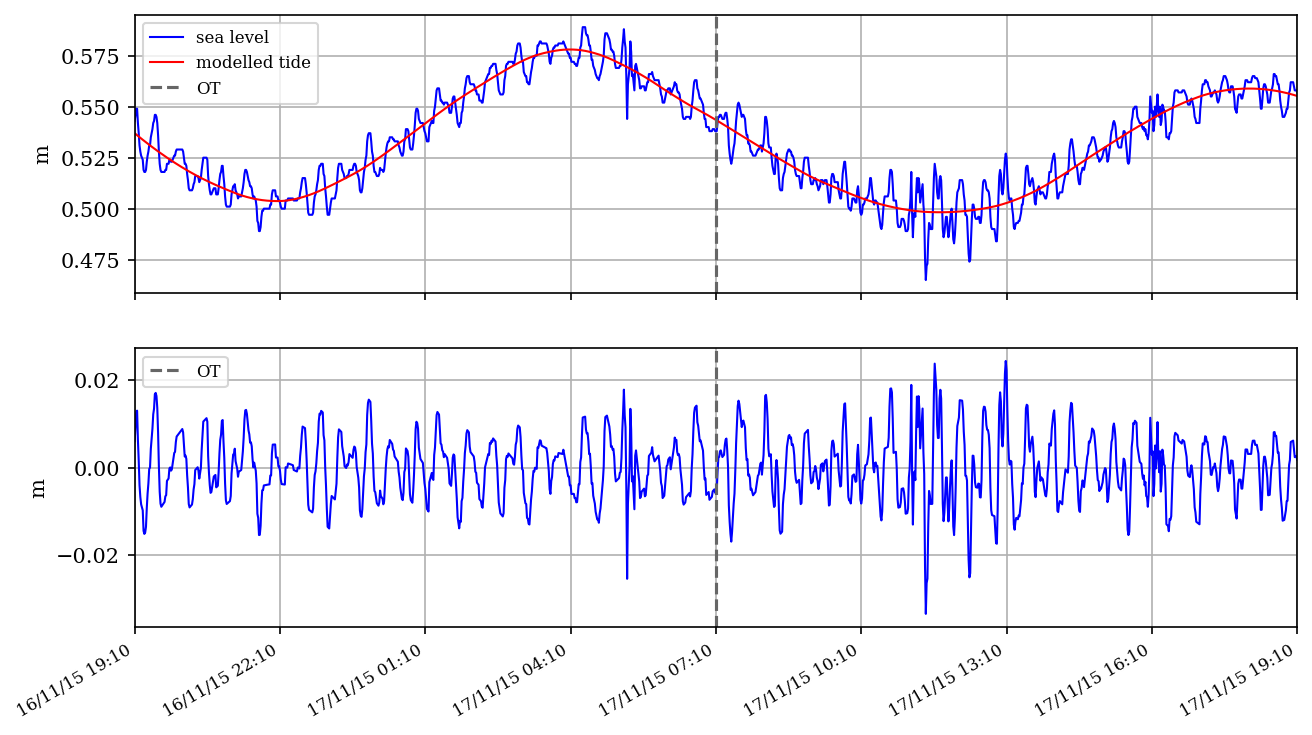

Supplement: Supplementary file 6 — Supplementary Dataset 3. Sea-level records. [file 41467_2021_25815_MOESM6_ESM.zip › sea_level_records/2015-11-17_Lefkas/kata.pr1.rmn.png]

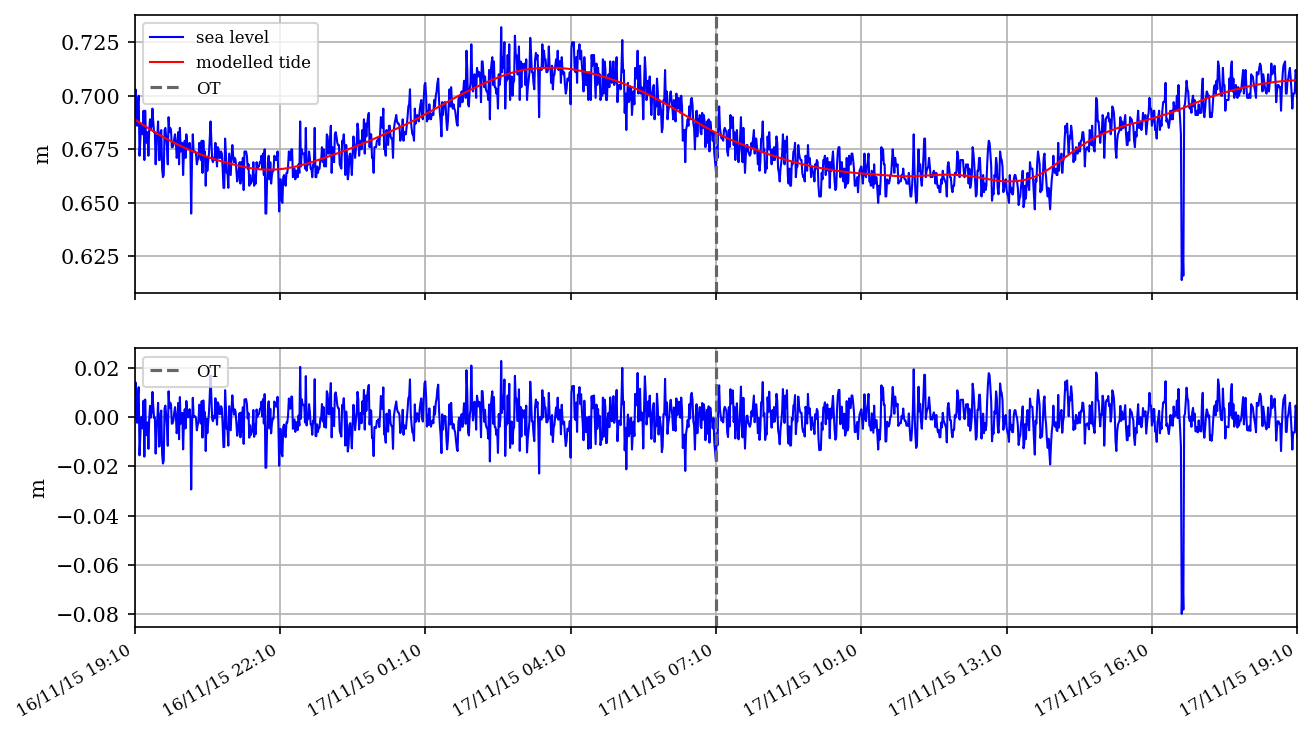

Supplement: Supplementary file 6 — Supplementary Dataset 3. Sea-level records. [file 41467_2021_25815_MOESM6_ESM.zip › sea_level_records/2015-11-17_Lefkas/koro.rad.rmn.png]

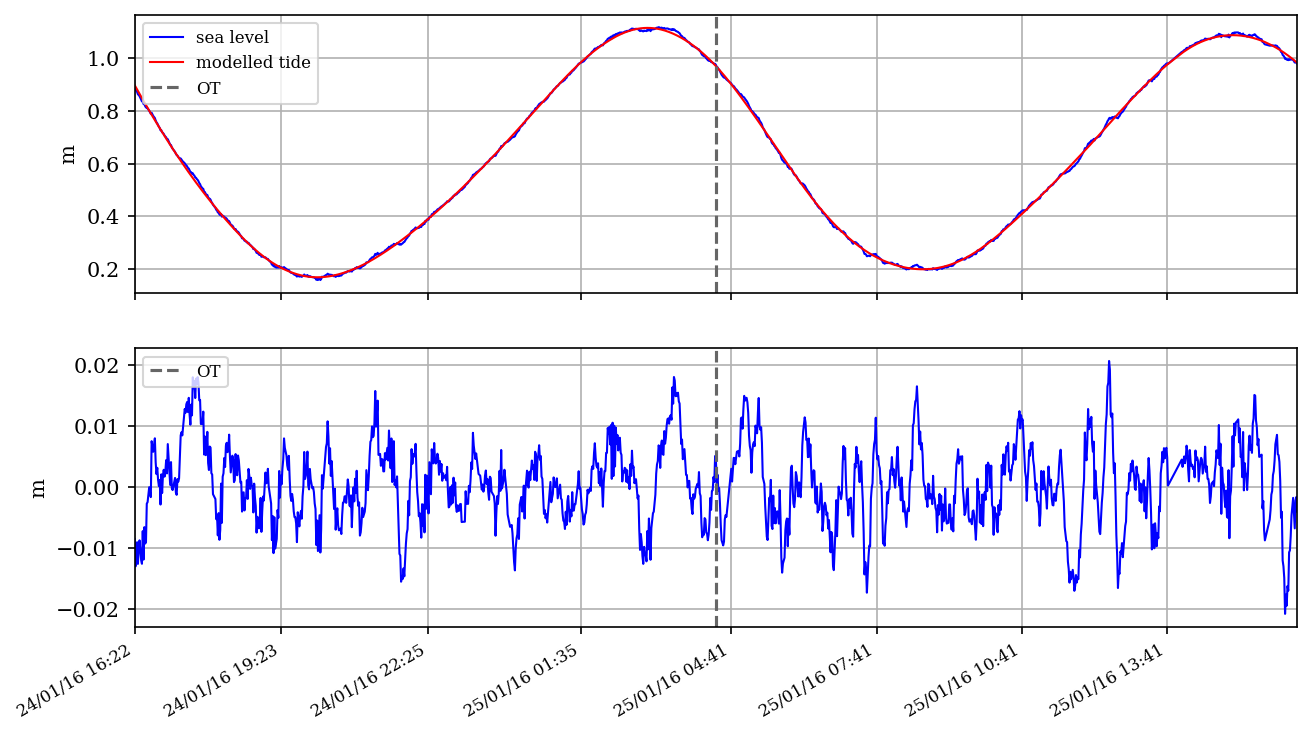

Supplement: Supplementary file 6 — Supplementary Dataset 3. Sea-level records. [file 41467_2021_25815_MOESM6_ESM.zip › sea_level_records/2016-01-25_Gibraltar/alge.rad.rmn.png]

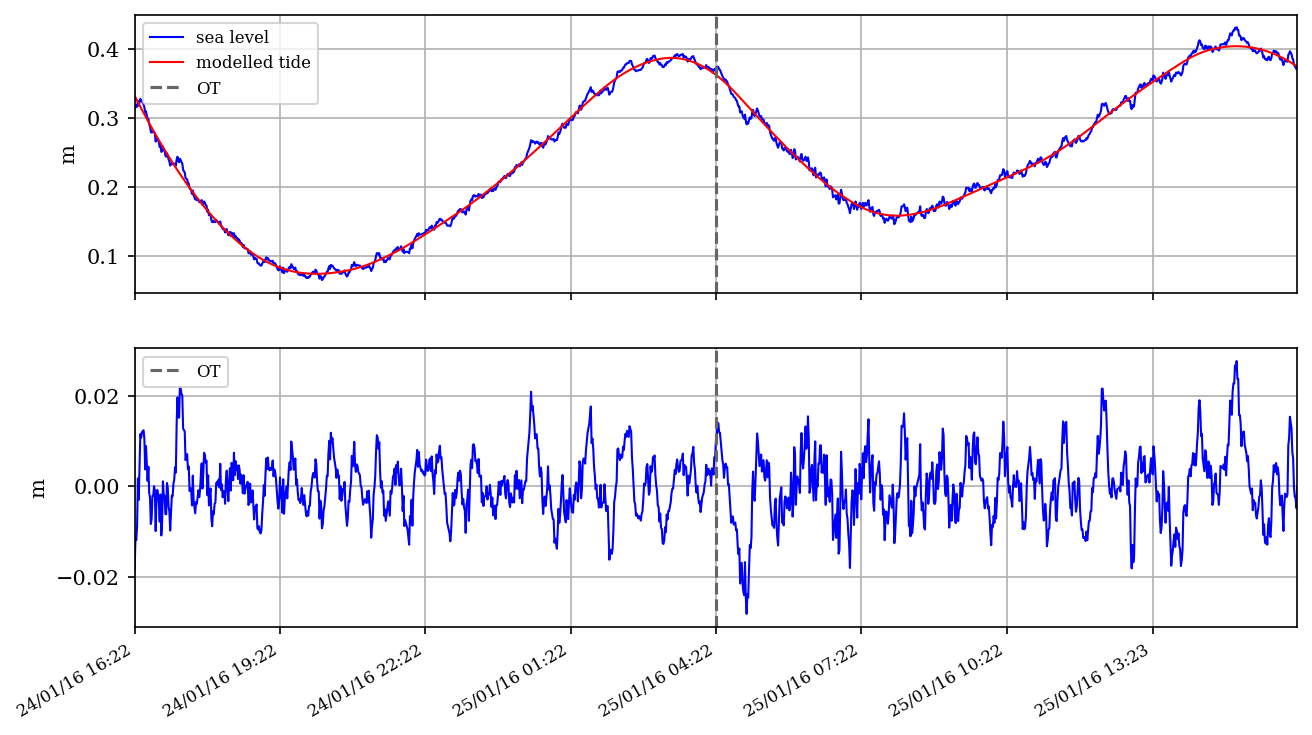

Supplement: Supplementary file 6 — Supplementary Dataset 3. Sea-level records. [file 41467_2021_25815_MOESM6_ESM.zip › sea_level_records/2016-01-25_Gibraltar/alme.rad.rmn.png]

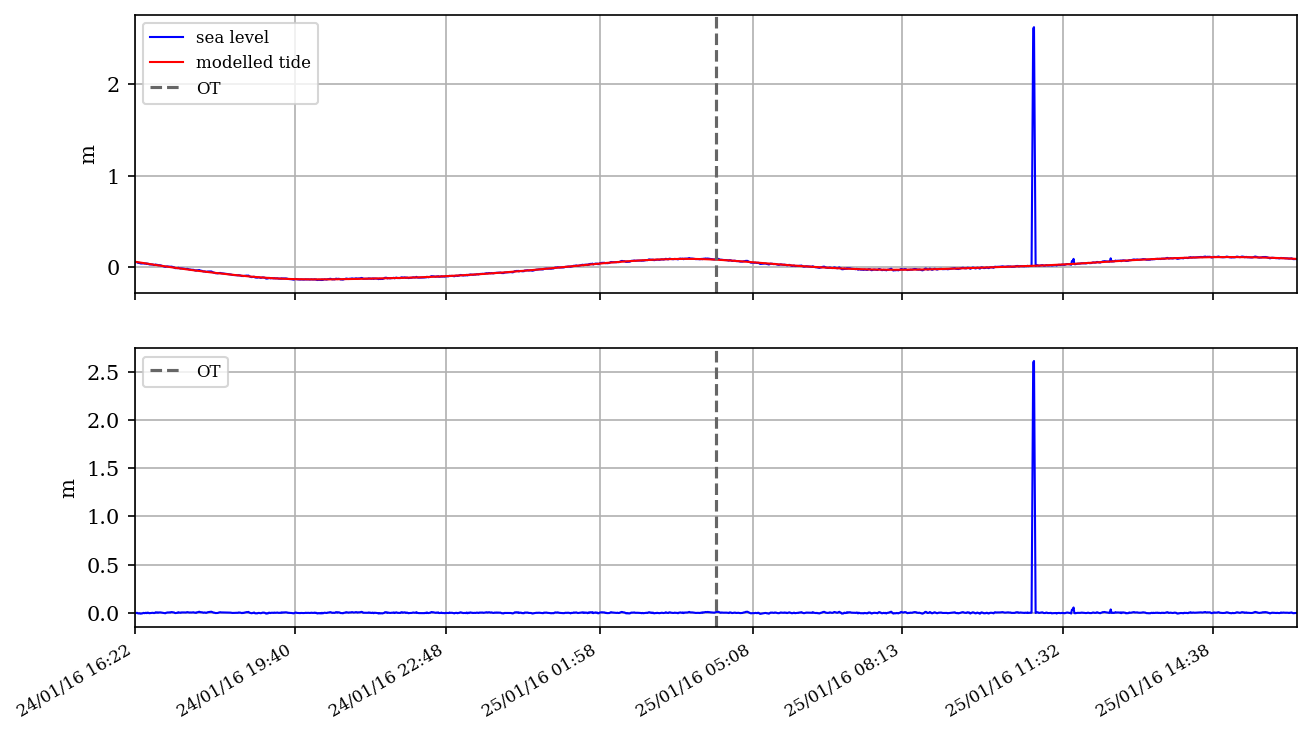

Supplement: Supplementary file 6 — Supplementary Dataset 3. Sea-level records. [file 41467_2021_25815_MOESM6_ESM.zip › sea_level_records/2016-01-25_Gibraltar/carb.rad.rmn.png]

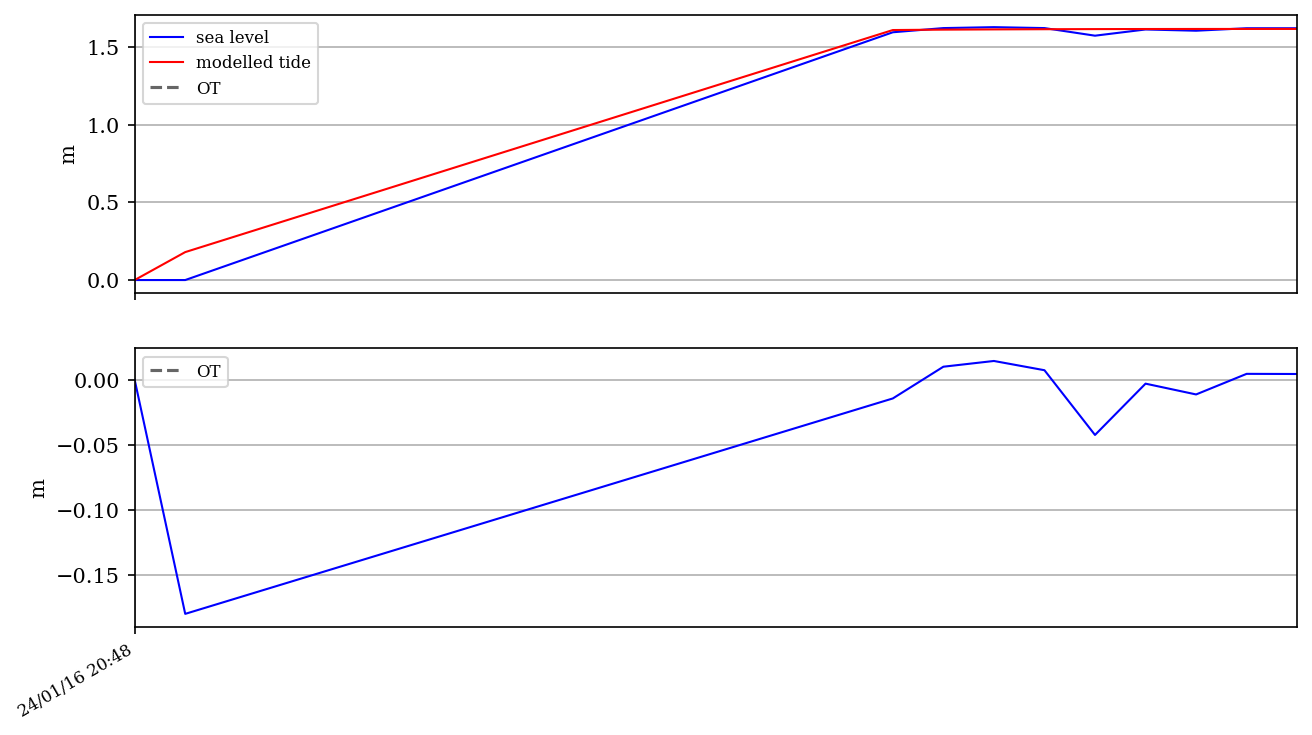

Supplement: Supplementary file 6 — Supplementary Dataset 3. Sea-level records. [file 41467_2021_25815_MOESM6_ESM.zip › sea_level_records/2016-01-25_Gibraltar/carg.rad.rmn.png]

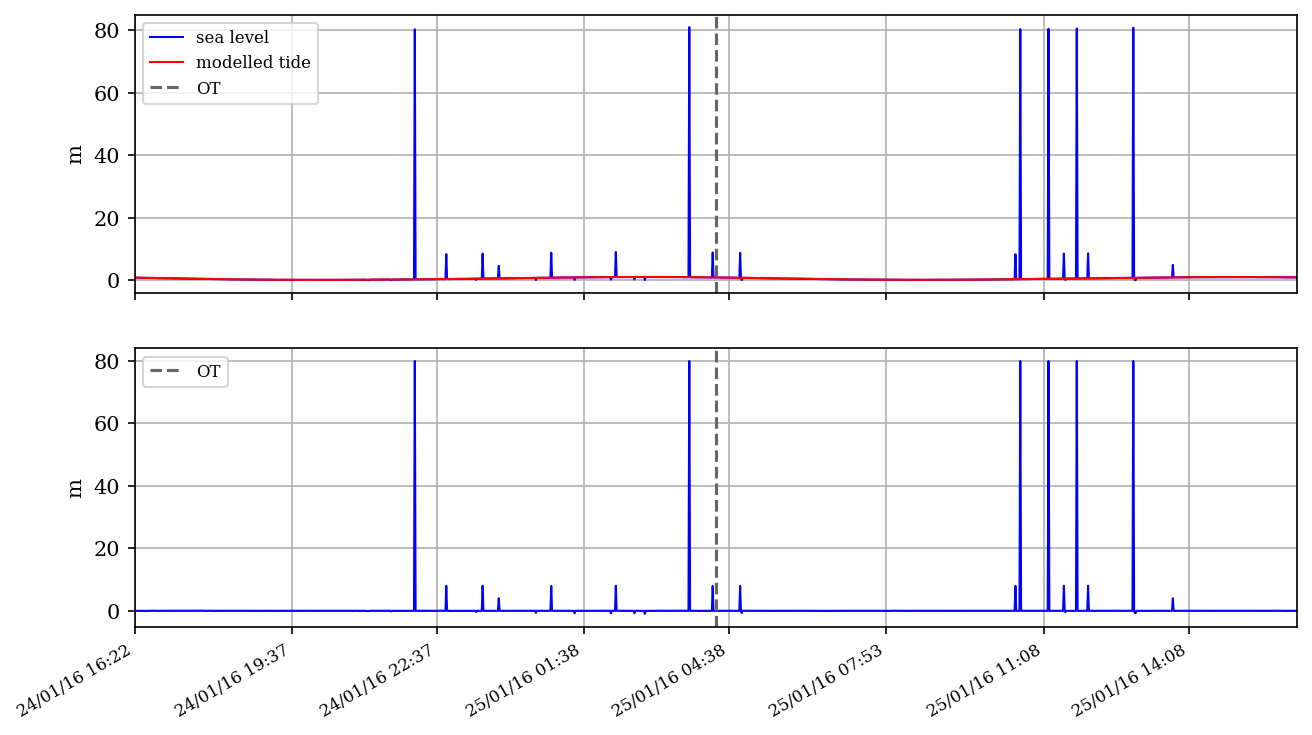

Supplement: Supplementary file 6 — Supplementary Dataset 3. Sea-level records. [file 41467_2021_25815_MOESM6_ESM.zip › sea_level_records/2016-01-25_Gibraltar/gibr2.rad.rmn.png]

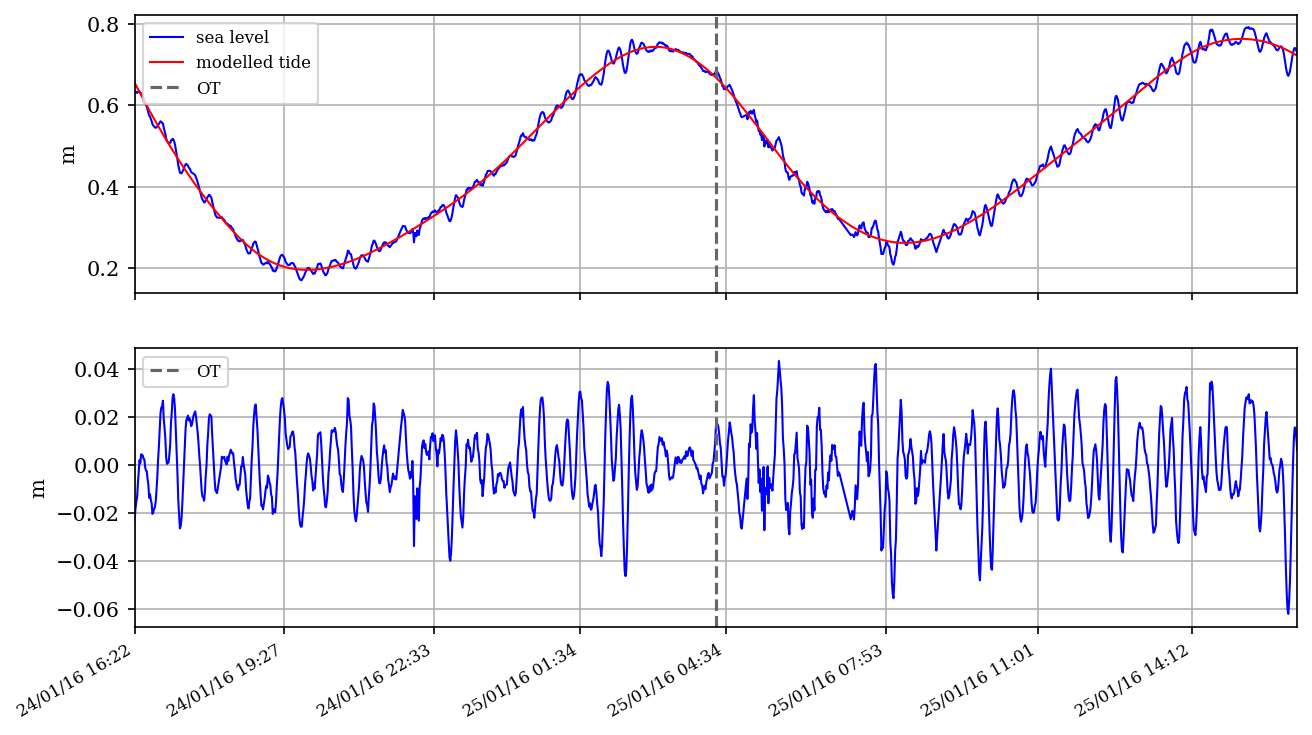

Supplement: Supplementary file 6 — Supplementary Dataset 3. Sea-level records. [file 41467_2021_25815_MOESM6_ESM.zip › sea_level_records/2016-01-25_Gibraltar/mal3.rad.rmn.png]

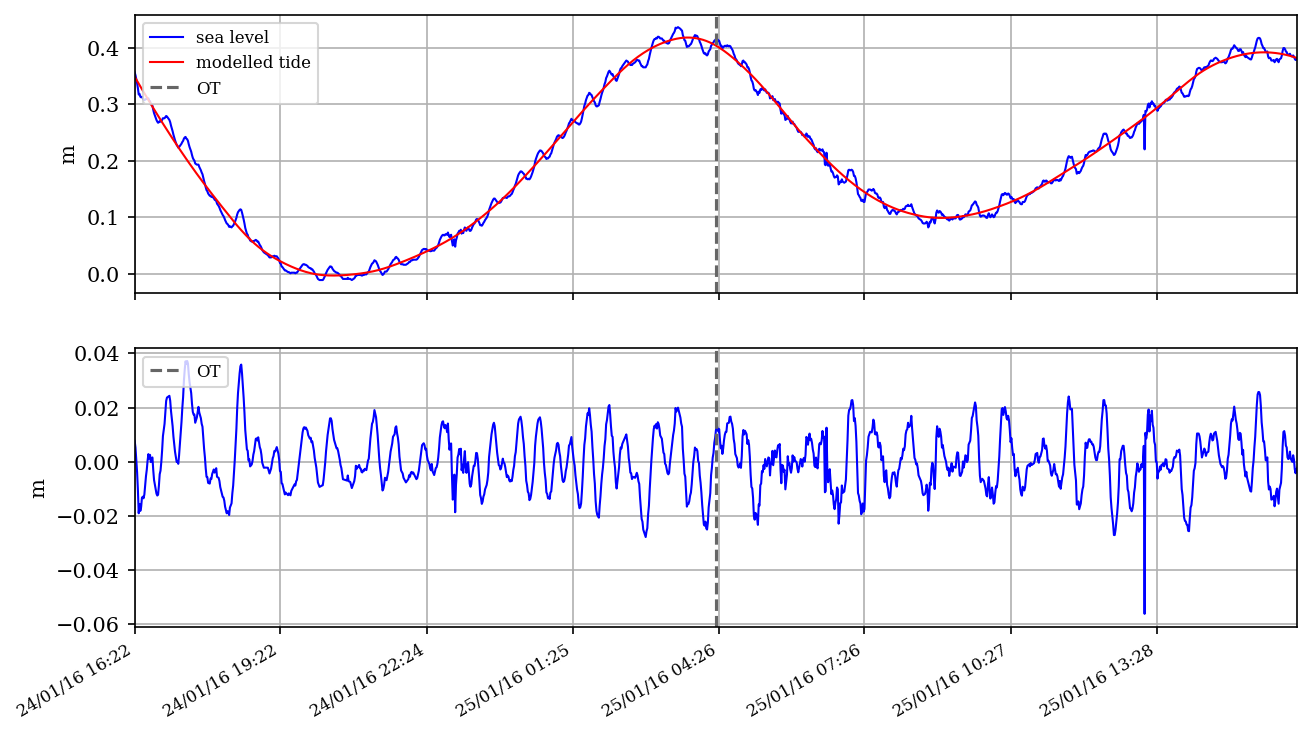

Supplement: Supplementary file 6 — Supplementary Dataset 3. Sea-level records. [file 41467_2021_25815_MOESM6_ESM.zip › sea_level_records/2016-01-25_Gibraltar/meli.rad.rmn.png]

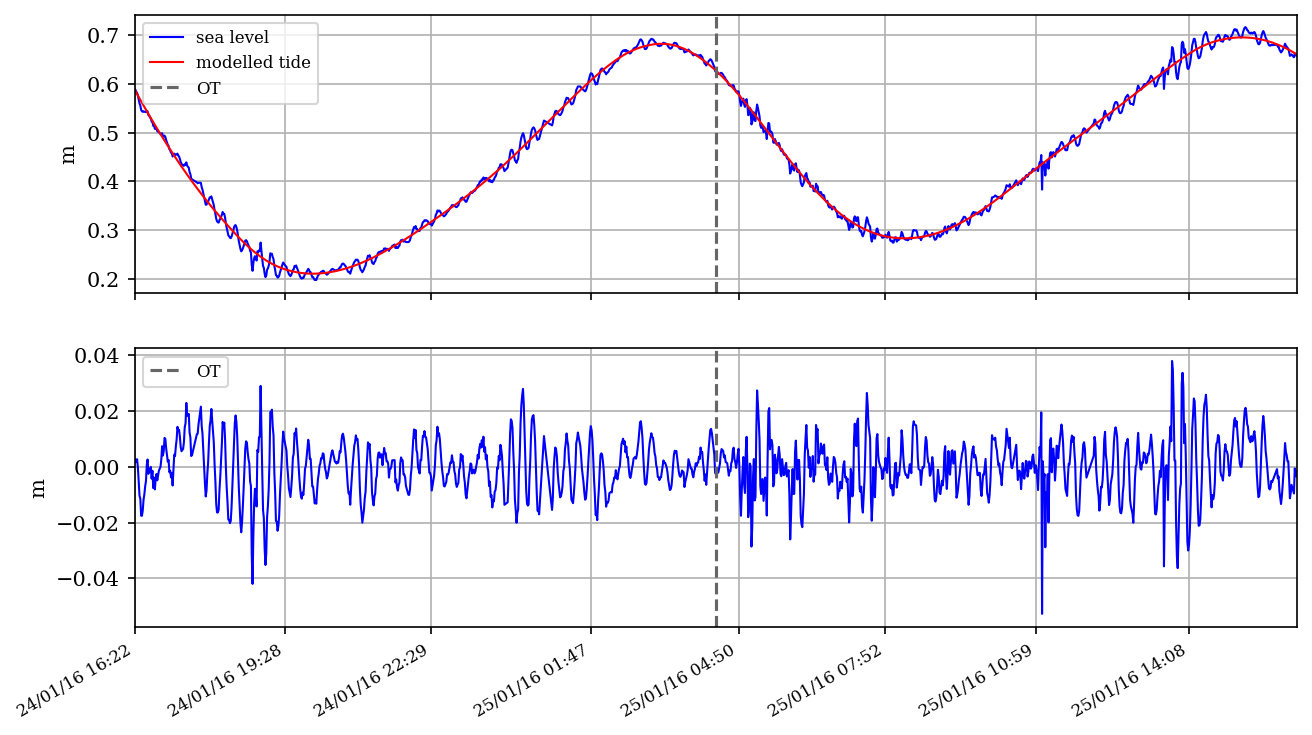

Supplement: Supplementary file 6 — Supplementary Dataset 3. Sea-level records. [file 41467_2021_25815_MOESM6_ESM.zip › sea_level_records/2016-01-25_Gibraltar/motr.rad.rmn.png]

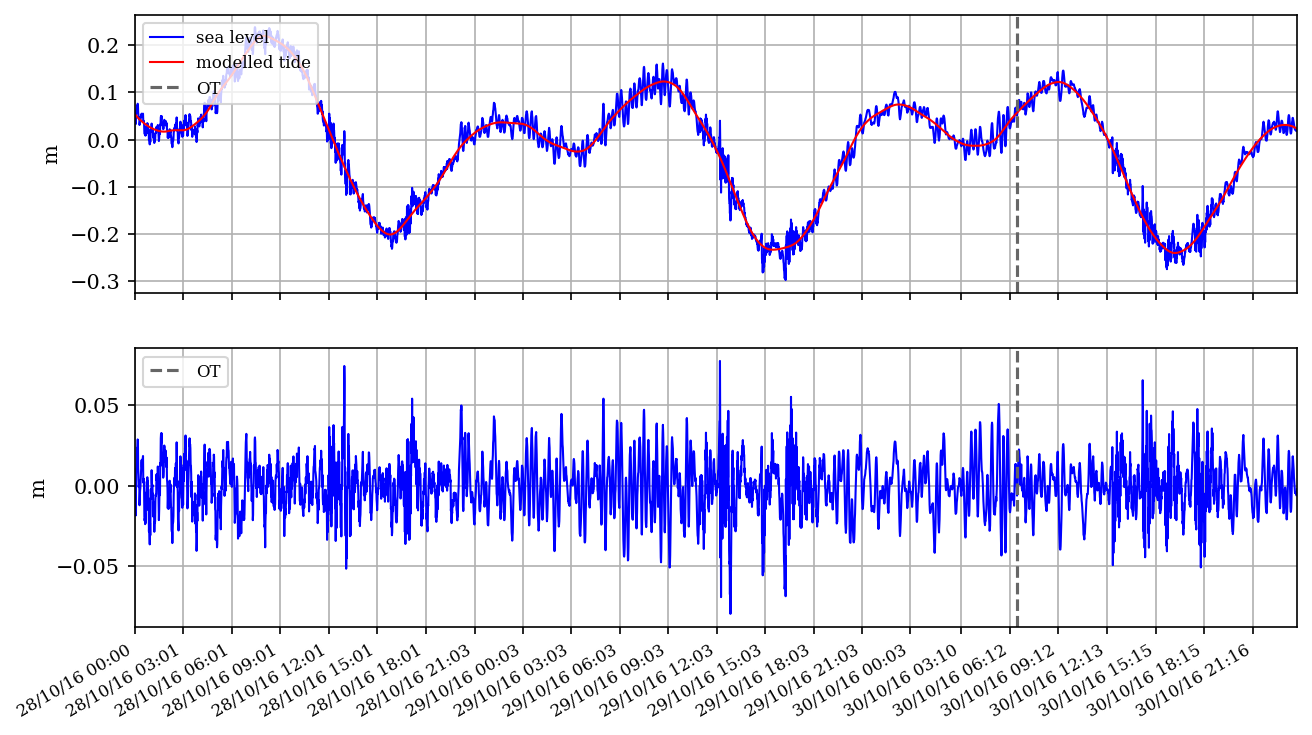

Supplement: Supplementary file 6 — Supplementary Dataset 3. Sea-level records. [file 41467_2021_25815_MOESM6_ESM.zip › sea_level_records/2016-10-30_Norcia/AN15.rad.rmn.png]

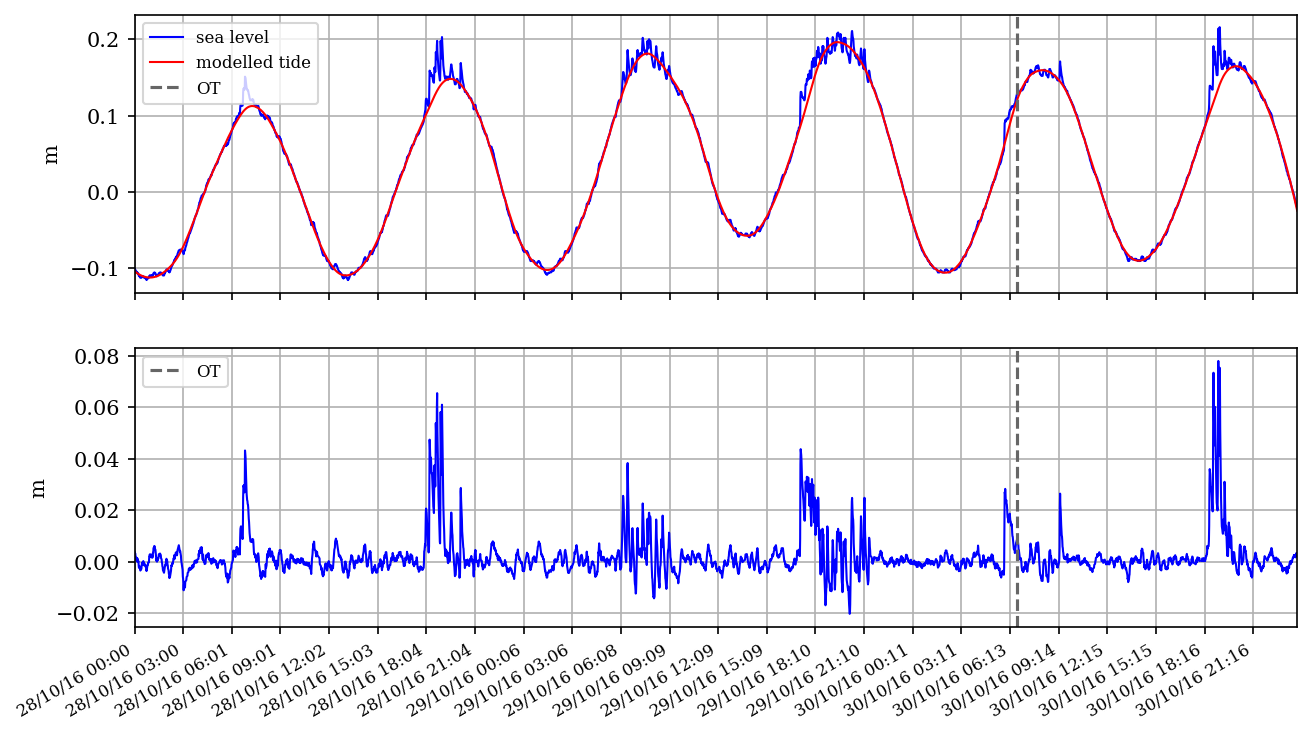

Supplement: Supplementary file 6 — Supplementary Dataset 3. Sea-level records. [file 41467_2021_25815_MOESM6_ESM.zip › sea_level_records/2016-10-30_Norcia/CI20.rad.rmn.png]

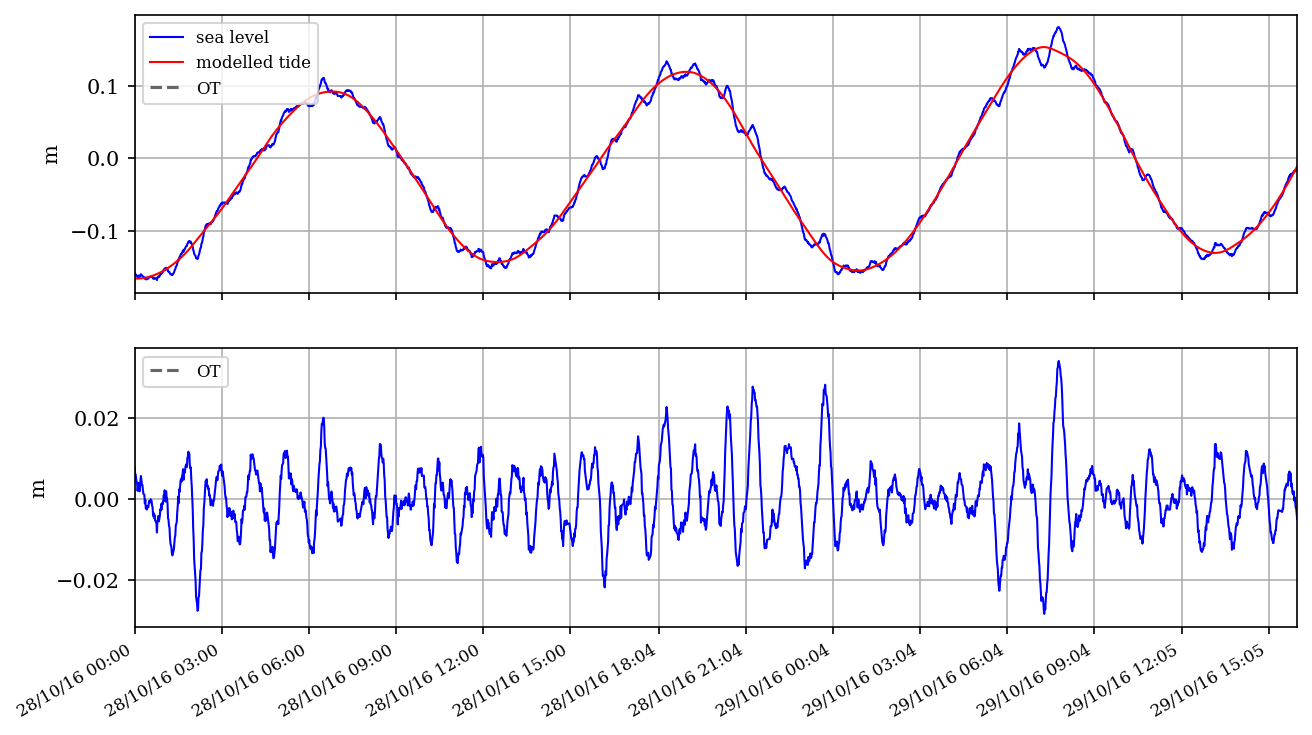

Supplement: Supplementary file 6 — Supplementary Dataset 3. Sea-level records. [file 41467_2021_25815_MOESM6_ESM.zip › sea_level_records/2016-10-30_Norcia/GA37.rad.rmn.png]

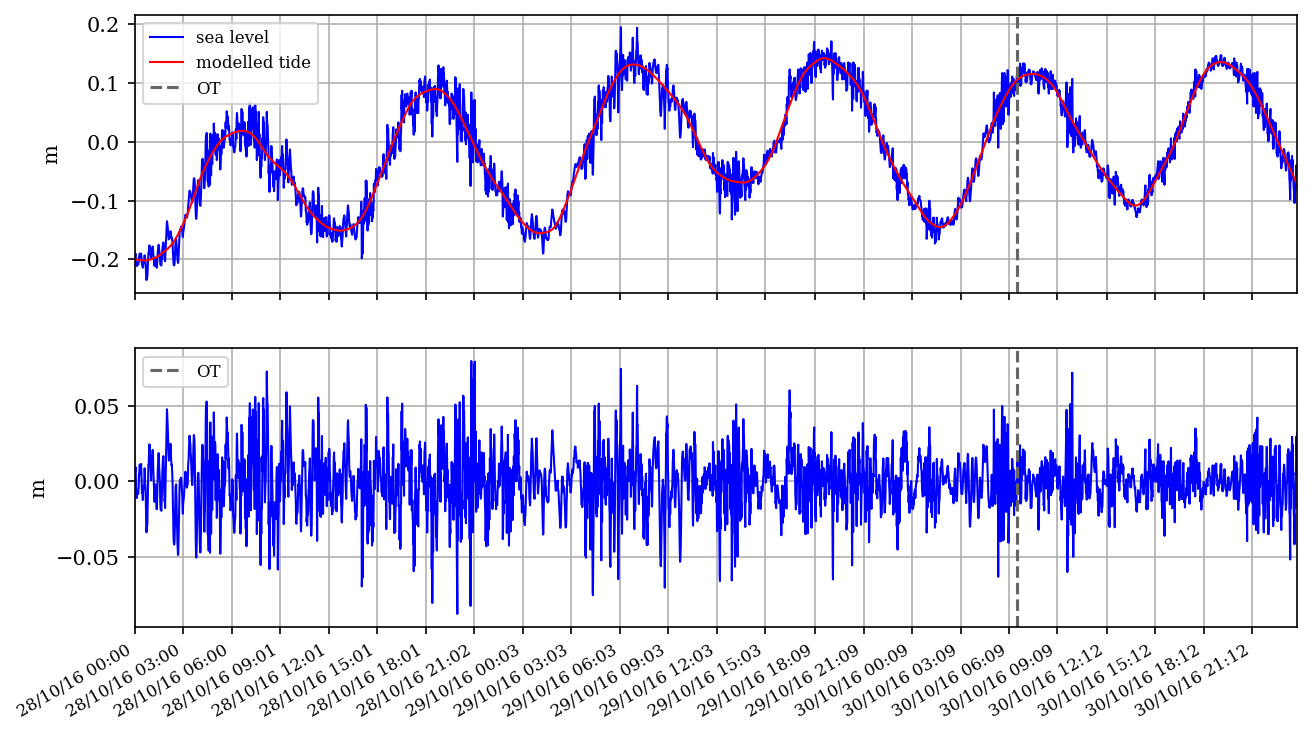

Supplement: Supplementary file 6 — Supplementary Dataset 3. Sea-level records. [file 41467_2021_25815_MOESM6_ESM.zip › sea_level_records/2016-10-30_Norcia/LI11.rad.rmn.png]

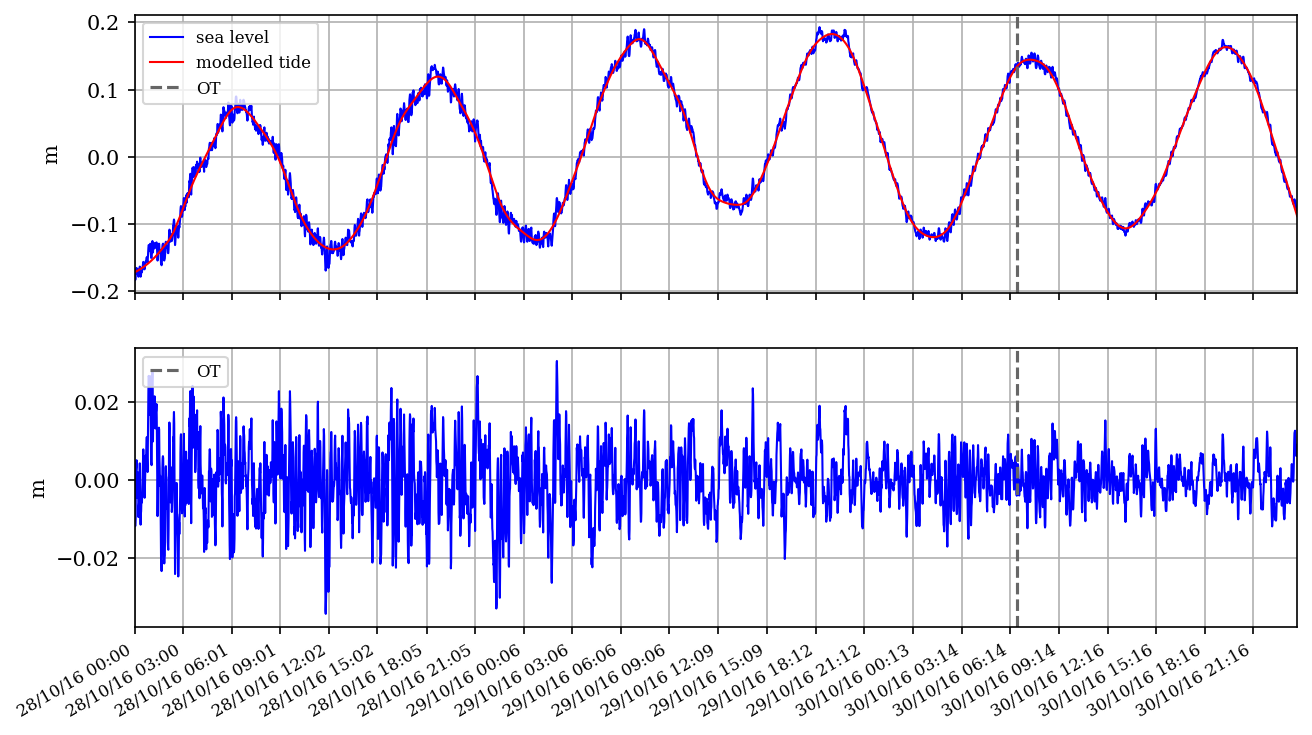

Supplement: Supplementary file 6 — Supplementary Dataset 3. Sea-level records. [file 41467_2021_25815_MOESM6_ESM.zip › sea_level_records/2016-10-30_Norcia/MC41.rad.rmn.png]

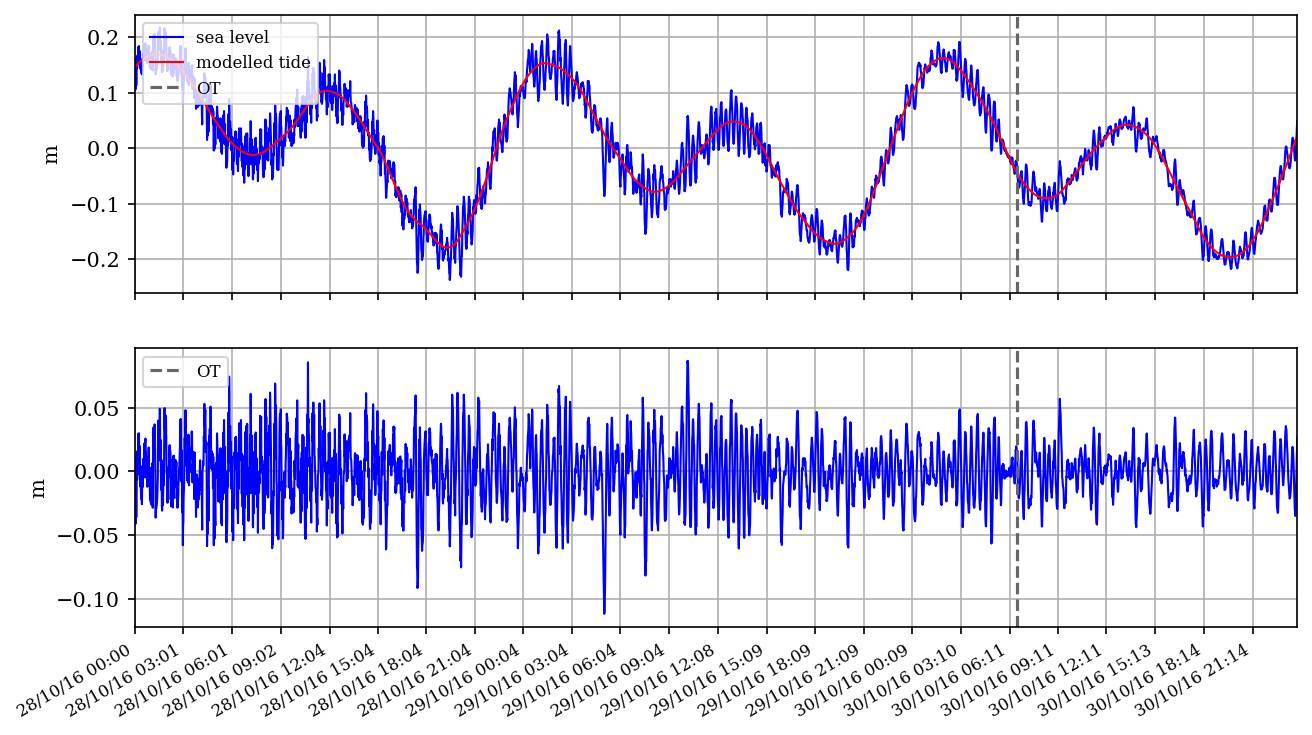

Supplement: Supplementary file 6 — Supplementary Dataset 3. Sea-level records. [file 41467_2021_25815_MOESM6_ESM.zip › sea_level_records/2016-10-30_Norcia/OR24.rad.rmn.png]

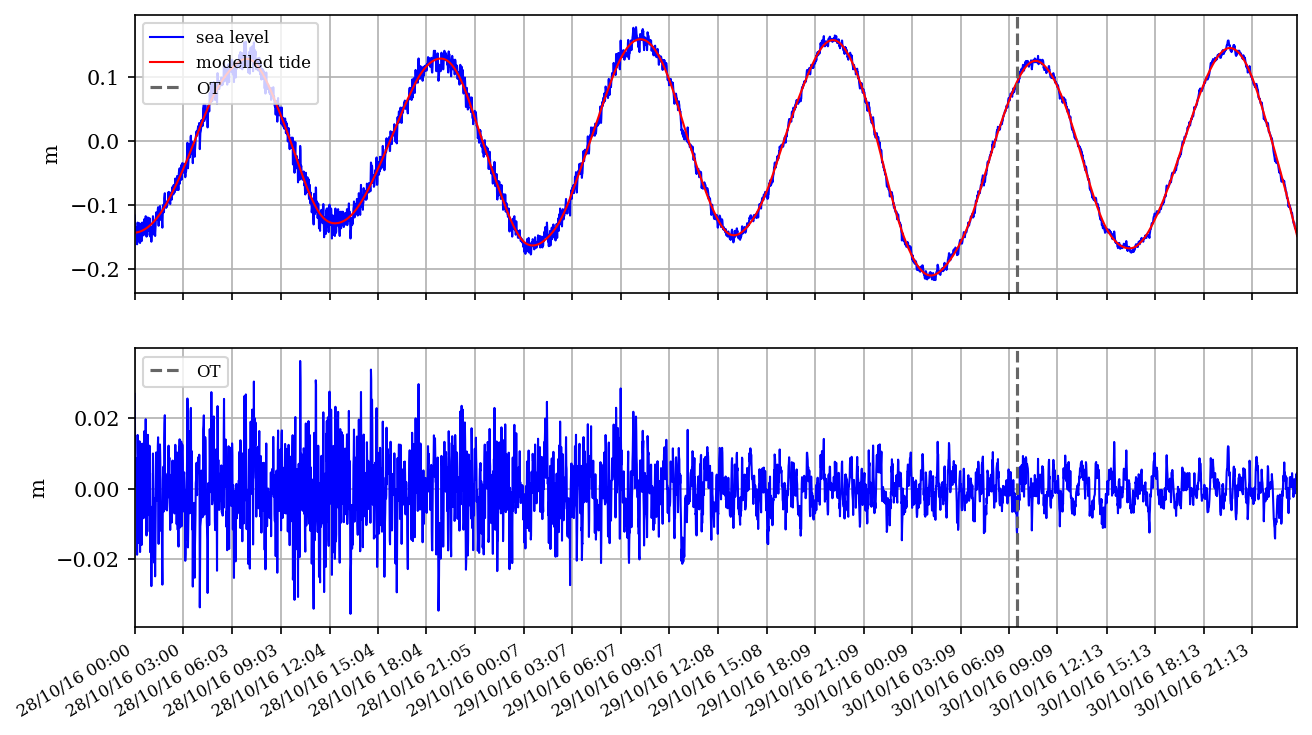

Supplement: Supplementary file 6 — Supplementary Dataset 3. Sea-level records. [file 41467_2021_25815_MOESM6_ESM.zip › sea_level_records/2016-10-30_Norcia/PL14.rad.rmn.png]

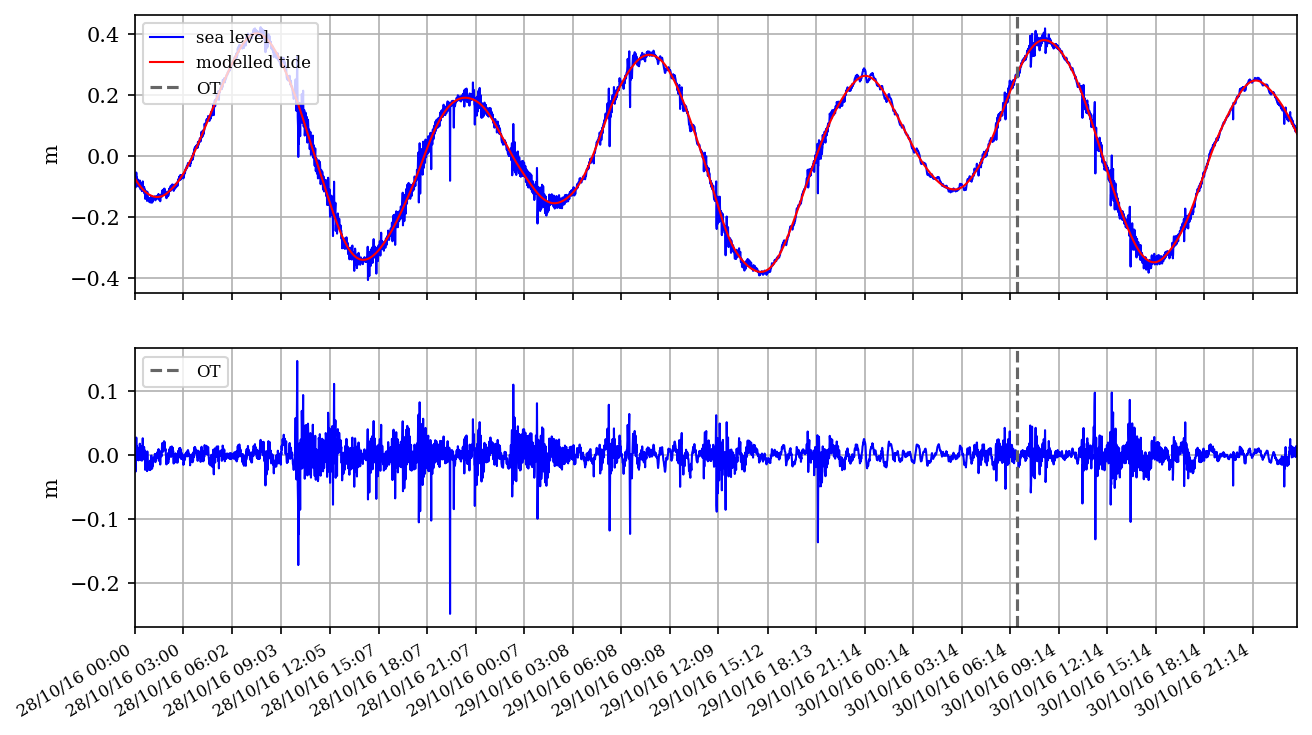

Supplement: Supplementary file 6 — Supplementary Dataset 3. Sea-level records. [file 41467_2021_25815_MOESM6_ESM.zip › sea_level_records/2016-10-30_Norcia/RA10.rad.rmn.png]

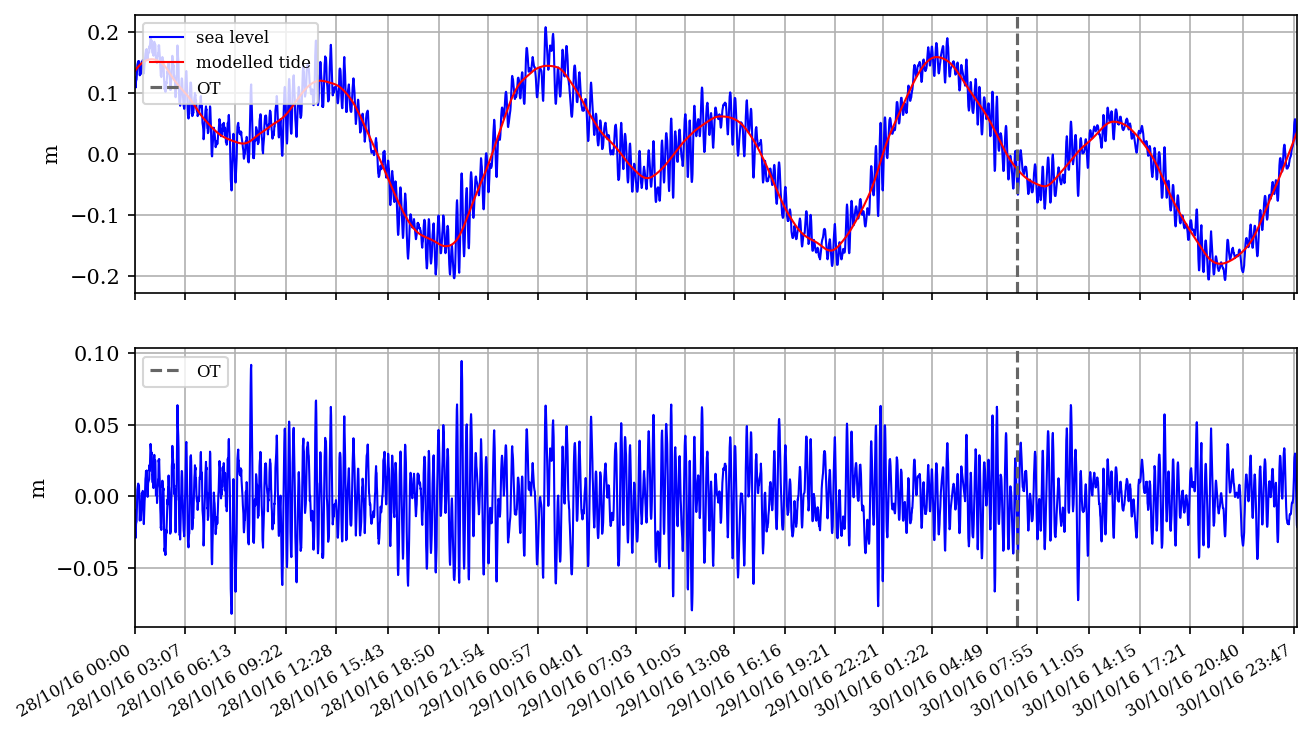

Supplement: Supplementary file 6 — Supplementary Dataset 3. Sea-level records. [file 41467_2021_25815_MOESM6_ESM.zip › sea_level_records/2016-10-30_Norcia/SB36.rad.rmn.png]

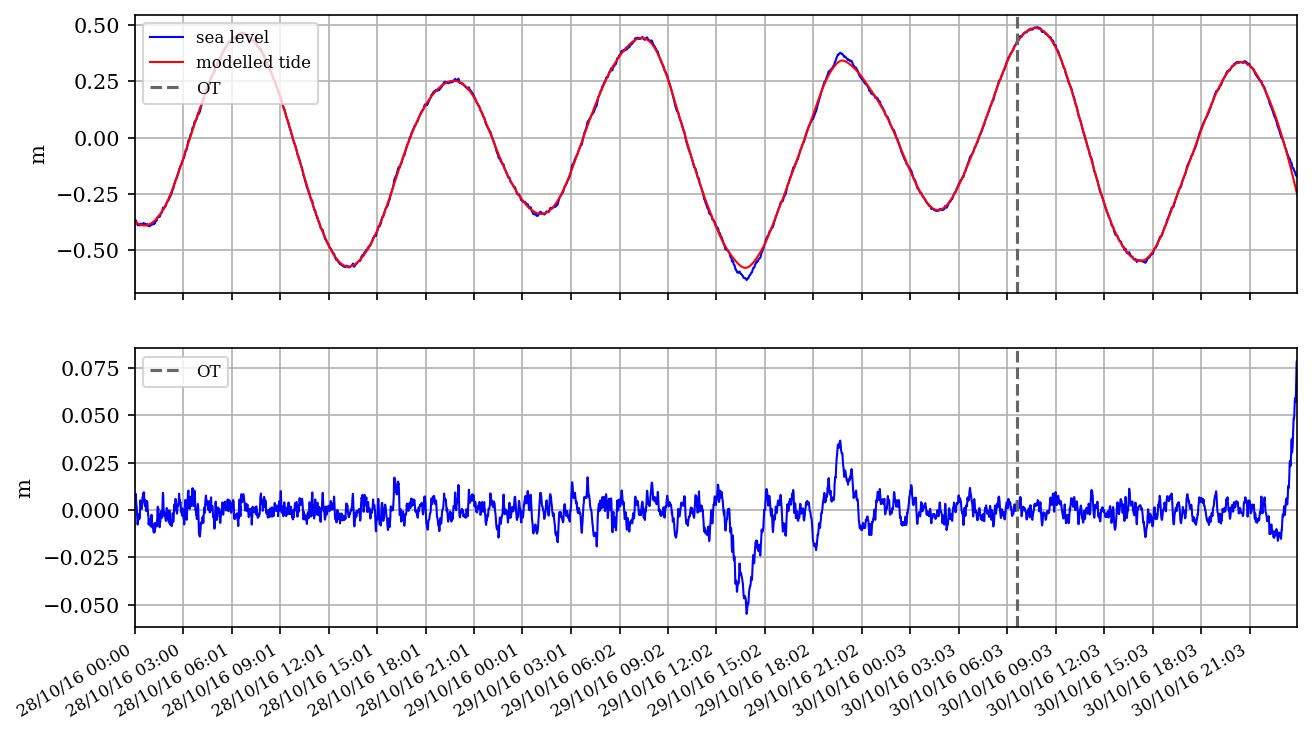

Supplement: Supplementary file 6 — Supplementary Dataset 3. Sea-level records. [file 41467_2021_25815_MOESM6_ESM.zip › sea_level_records/2016-10-30_Norcia/TR22.rad.rmn.png]

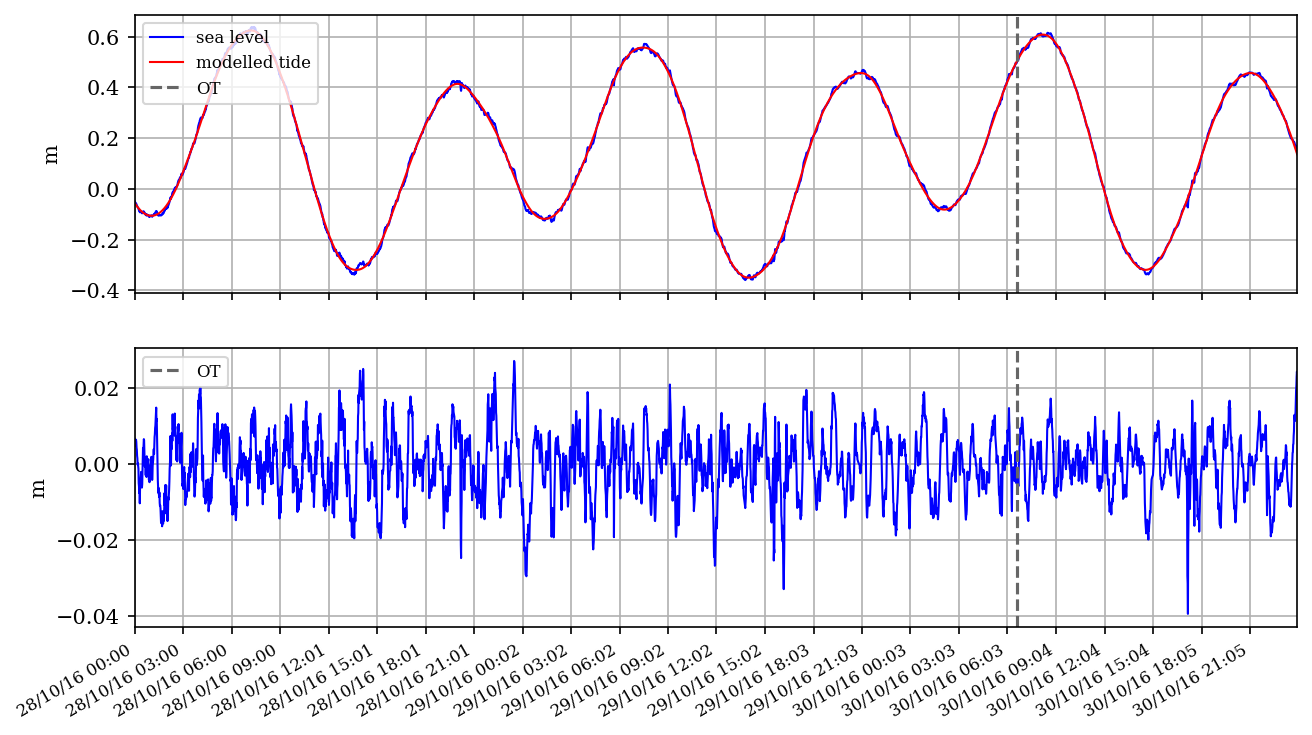

Supplement: Supplementary file 6 — Supplementary Dataset 3. Sea-level records. [file 41467_2021_25815_MOESM6_ESM.zip › sea_level_records/2016-10-30_Norcia/VE19.rad.rmn.png]

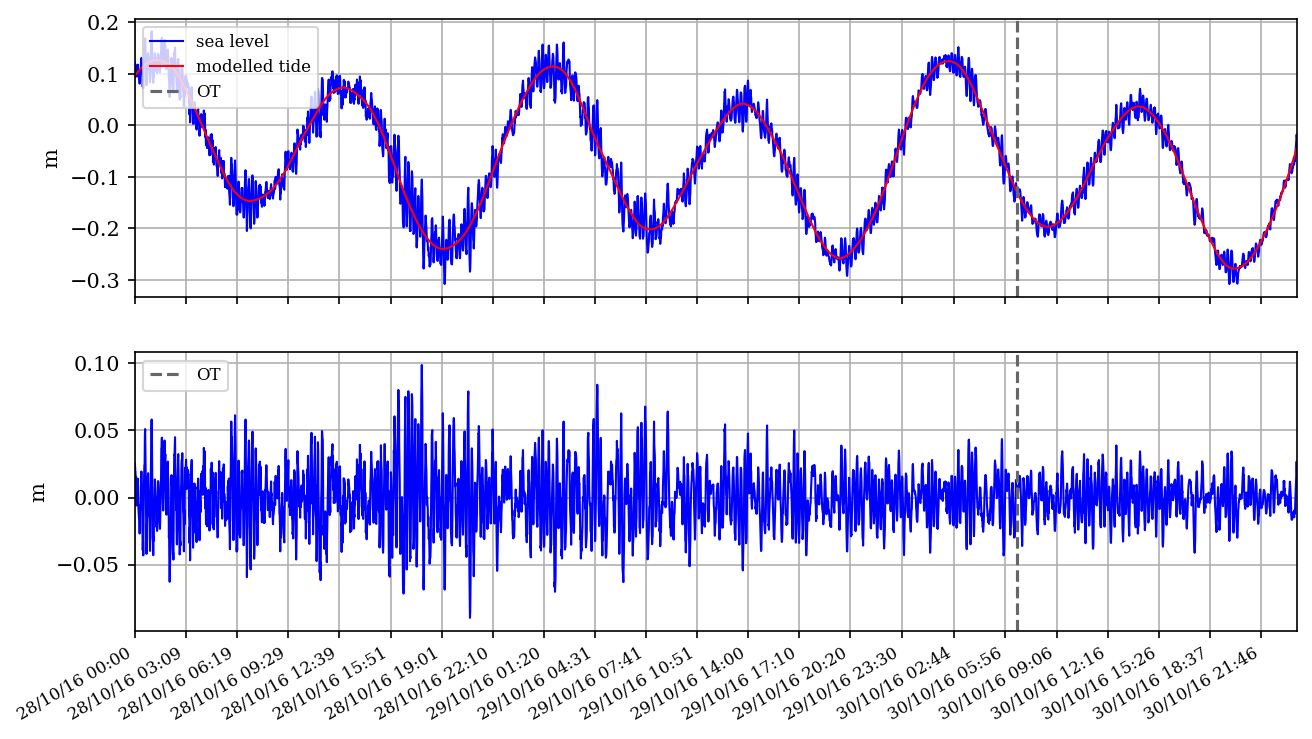

Supplement: Supplementary file 6 — Supplementary Dataset 3. Sea-level records. [file 41467_2021_25815_MOESM6_ESM.zip › sea_level_records/2016-10-30_Norcia/VI12.rad.rmn.png]

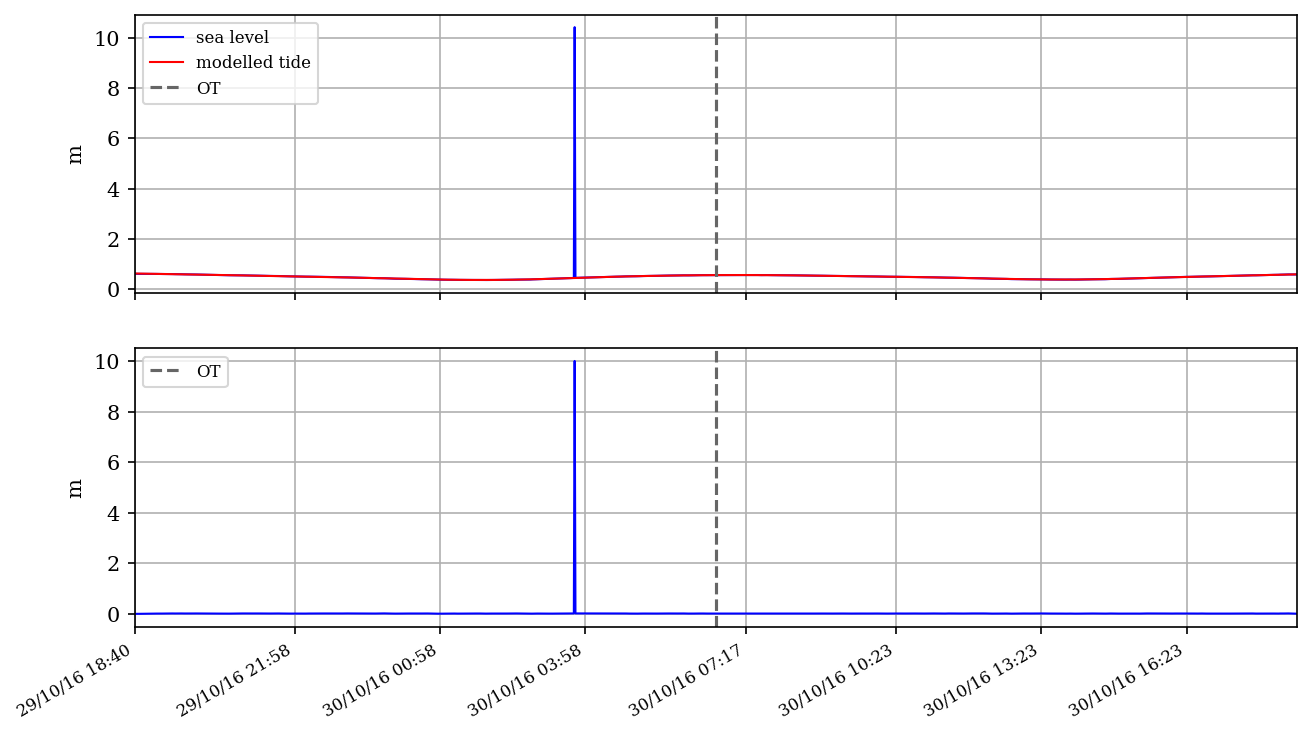

Supplement: Supplementary file 6 — Supplementary Dataset 3. Sea-level records. [file 41467_2021_25815_MOESM6_ESM.zip › sea_level_records/2016-10-30_Norcia/ajac2.rad.rmn.png]

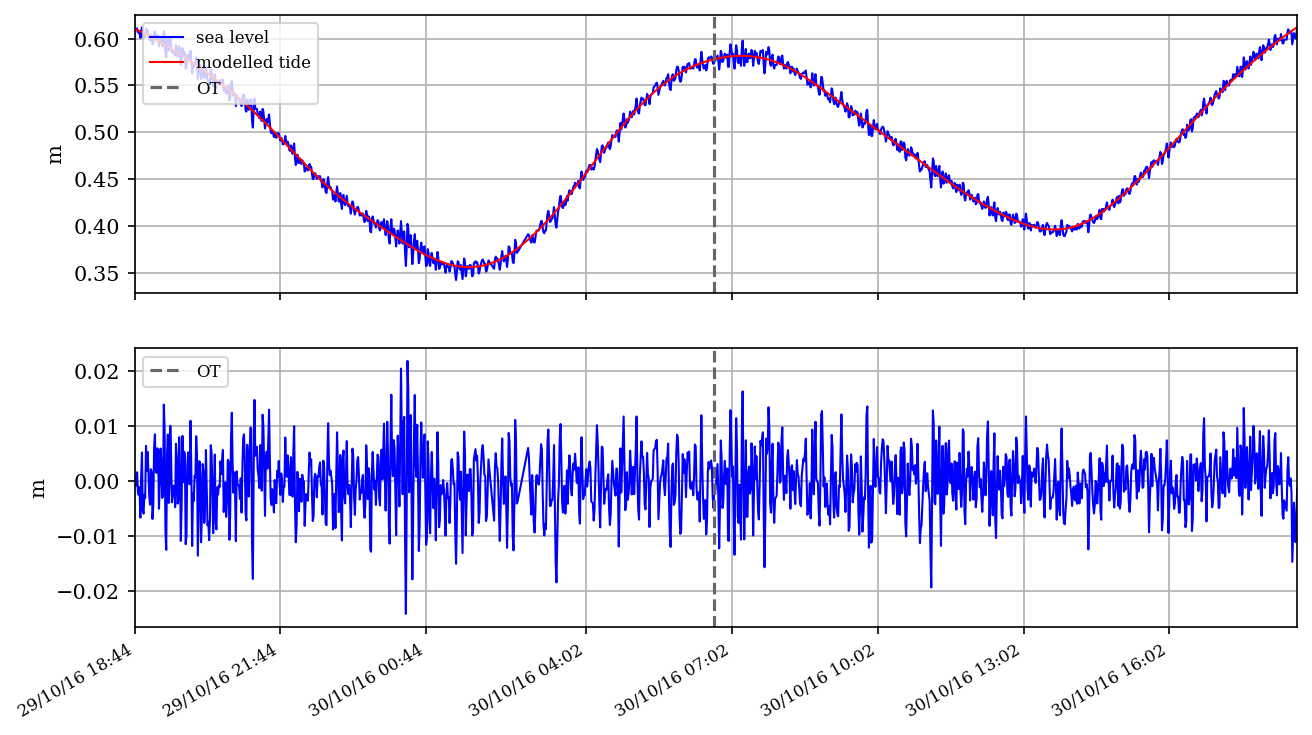

Supplement: Supplementary file 6 — Supplementary Dataset 3. Sea-level records. [file 41467_2021_25815_MOESM6_ESM.zip › sea_level_records/2016-10-30_Norcia/cent2.rad.rmn.png]

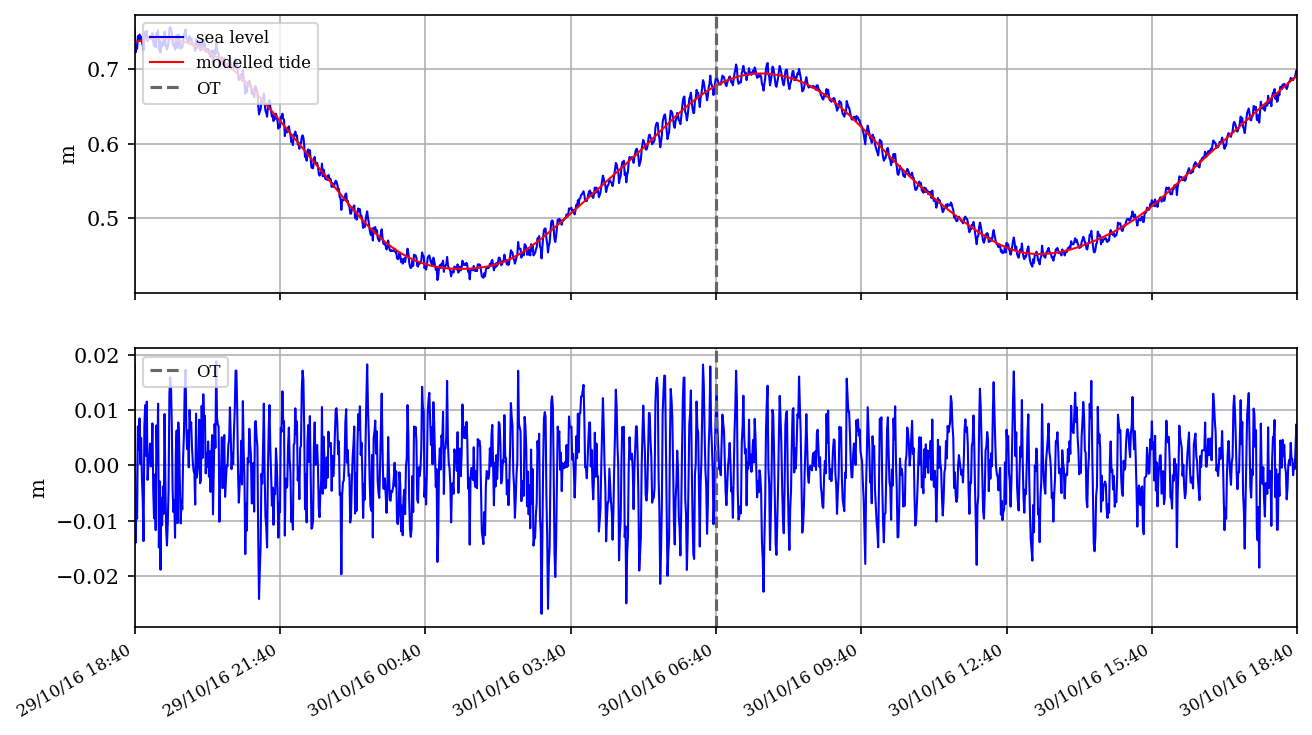

Supplement: Supplementary file 6 — Supplementary Dataset 3. Sea-level records. [file 41467_2021_25815_MOESM6_ESM.zip › sea_level_records/2016-10-30_Norcia/sole2.rad.rmn.png]

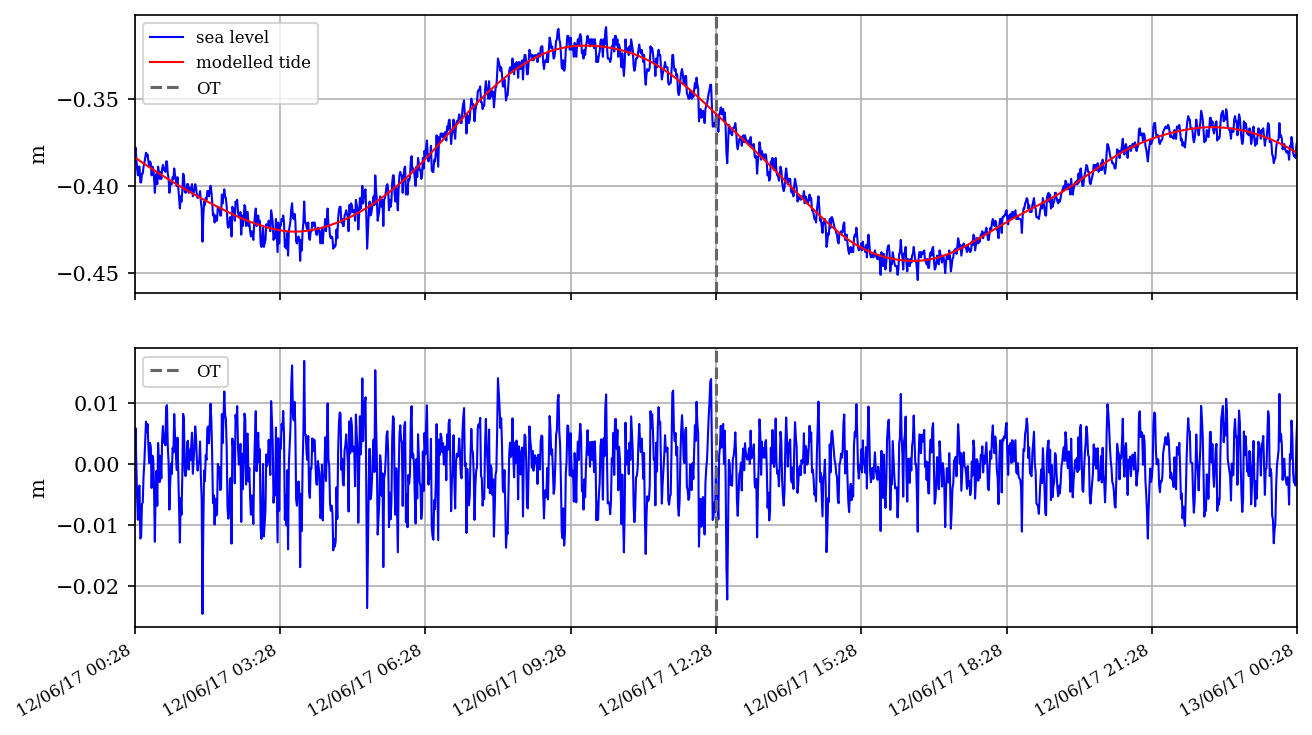

Supplement: Supplementary file 6 — Supplementary Dataset 3. Sea-level records. [file 41467_2021_25815_MOESM6_ESM.zip › sea_level_records/2017-06-12_Lesvos/NOA03.rad.rmn.png]

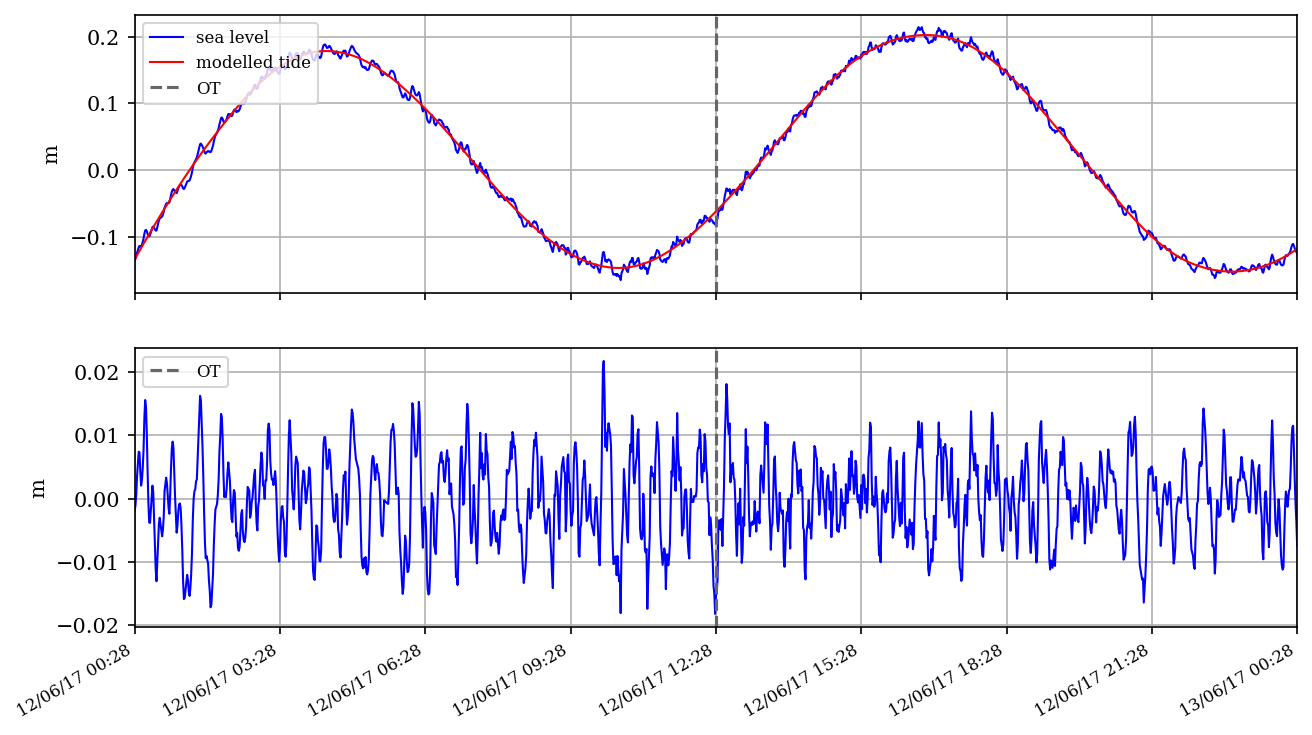

Supplement: Supplementary file 6 — Supplementary Dataset 3. Sea-level records. [file 41467_2021_25815_MOESM6_ESM.zip › sea_level_records/2017-06-12_Lesvos/NOA05.rad.rmn.png]

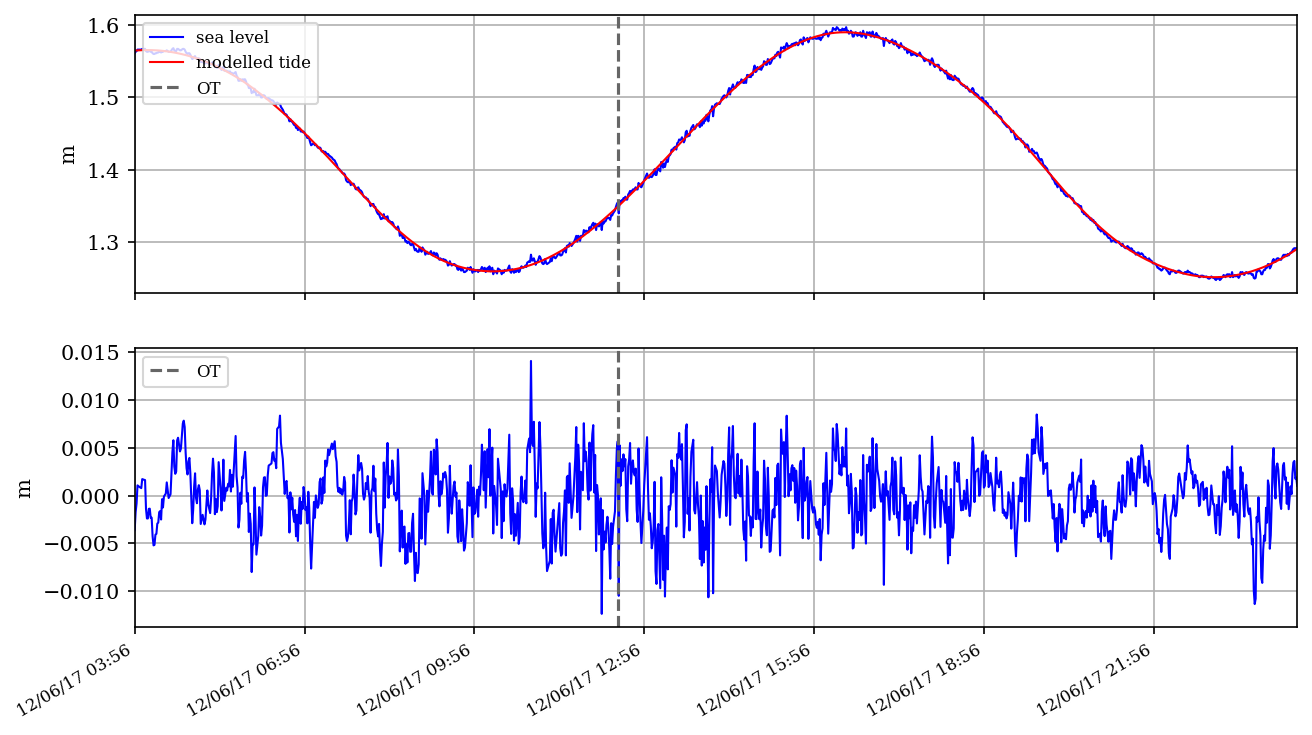

Supplement: Supplementary file 6 — Supplementary Dataset 3. Sea-level records. [file 41467_2021_25815_MOESM6_ESM.zip › sea_level_records/2017-06-12_Lesvos/NOA06.rad.rmn.png]

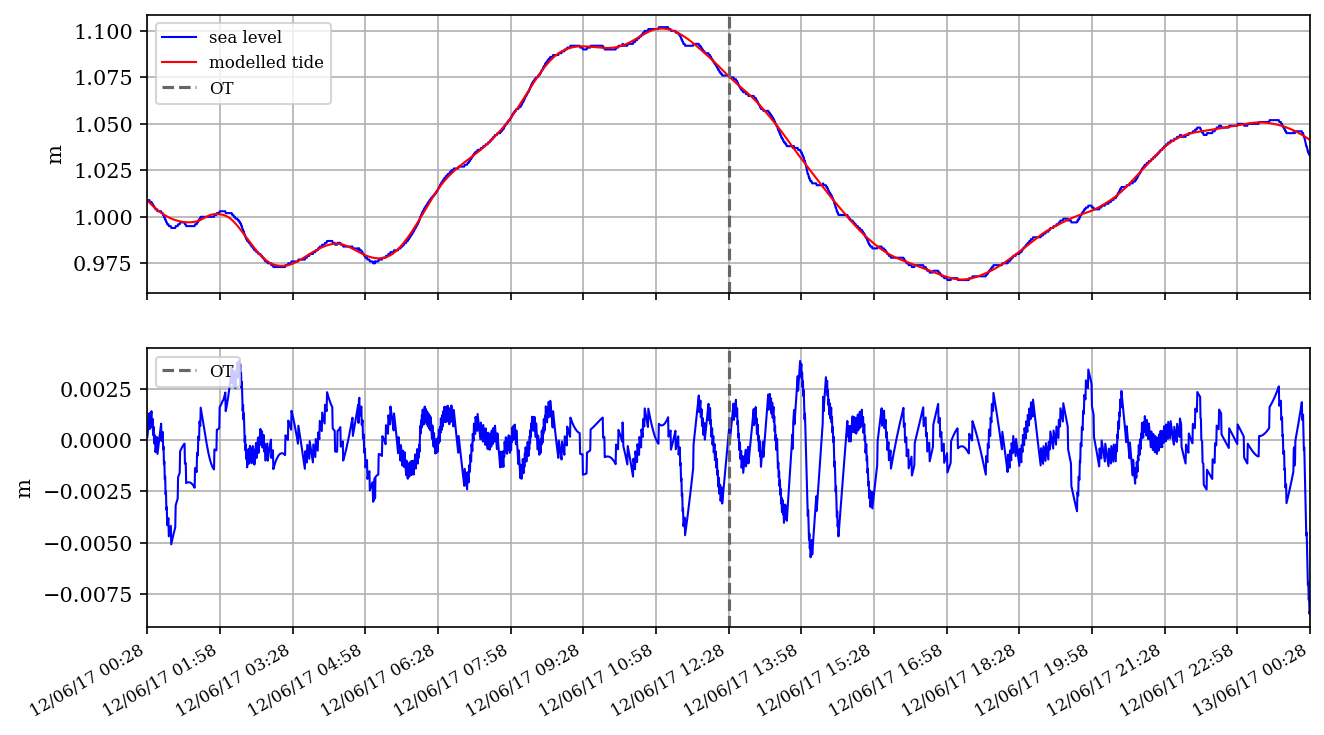

Supplement: Supplementary file 6 — Supplementary Dataset 3. Sea-level records. [file 41467_2021_25815_MOESM6_ESM.zip › sea_level_records/2017-06-12_Lesvos/bodru.rad.rmn.png]

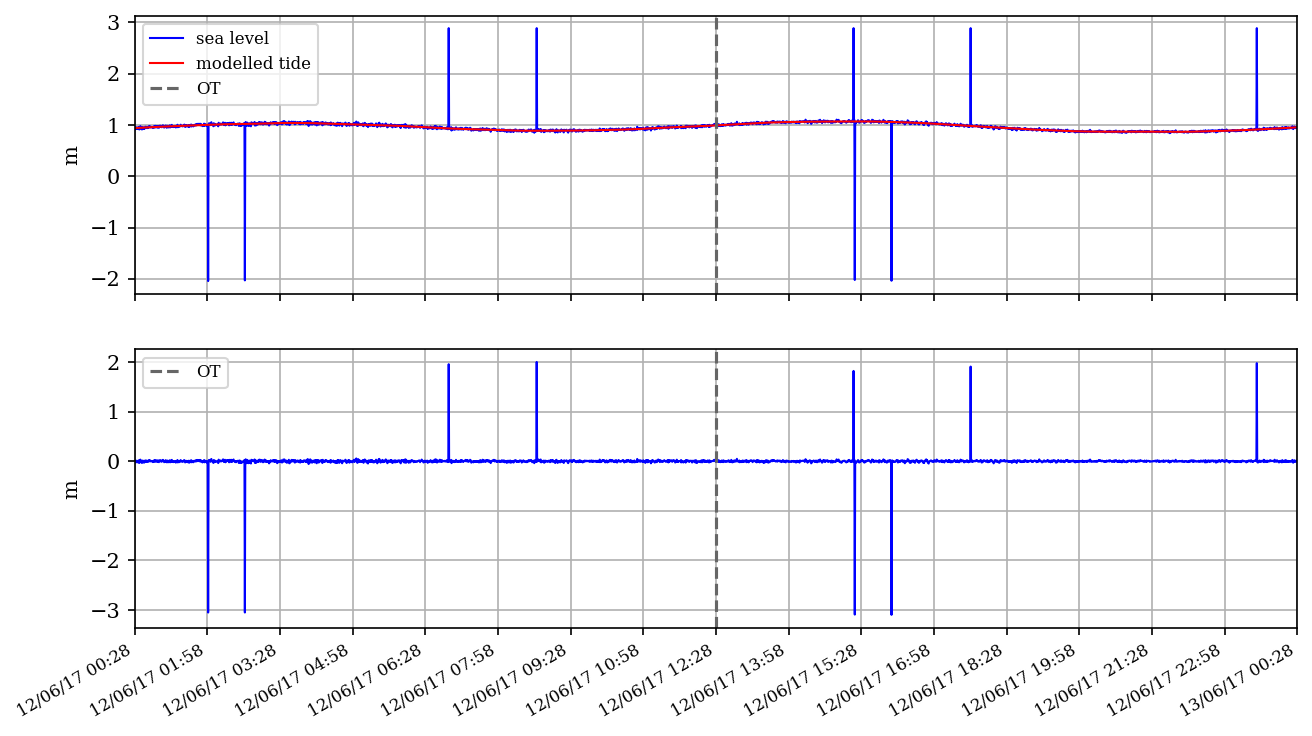

Supplement: Supplementary file 6 — Supplementary Dataset 3. Sea-level records. [file 41467_2021_25815_MOESM6_ESM.zip › sea_level_records/2017-06-12_Lesvos/gokce.rad.rmn.png]

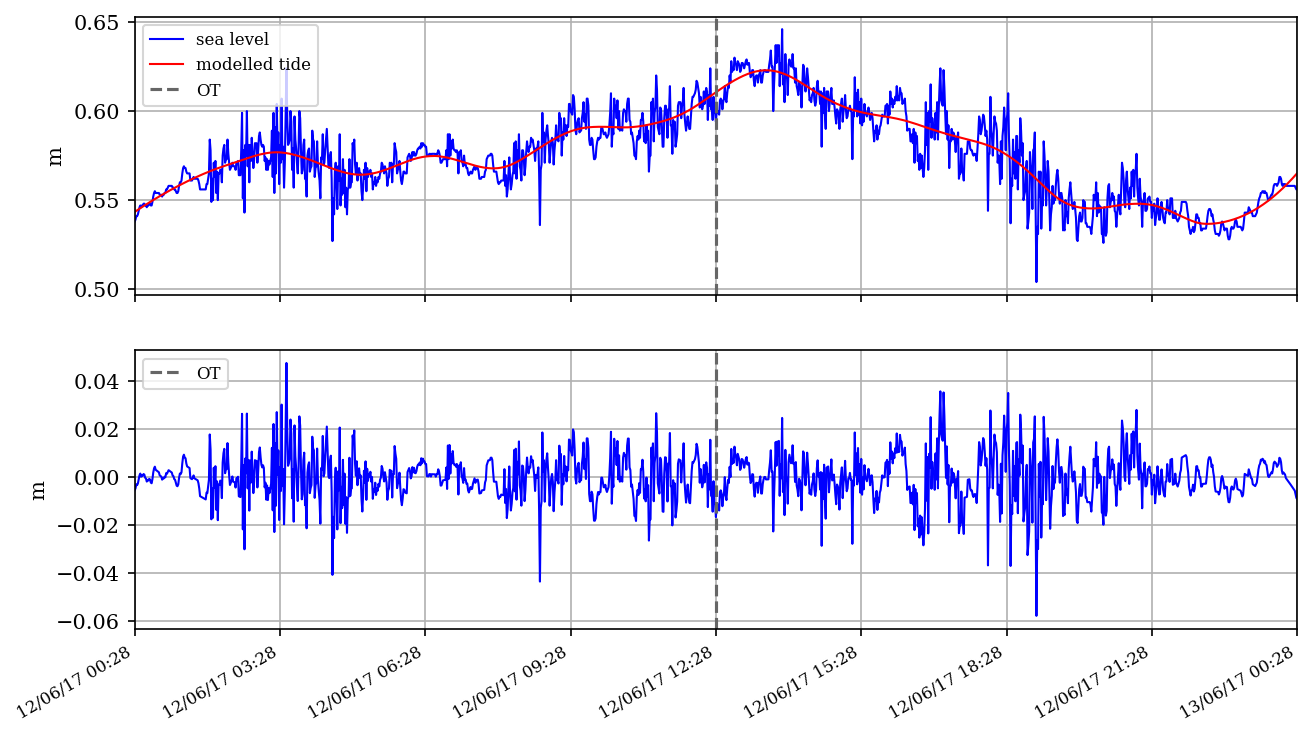

Supplement: Supplementary file 6 — Supplementary Dataset 3. Sea-level records. [file 41467_2021_25815_MOESM6_ESM.zip › sea_level_records/2017-06-12_Lesvos/peir.pr1.rmn.png]

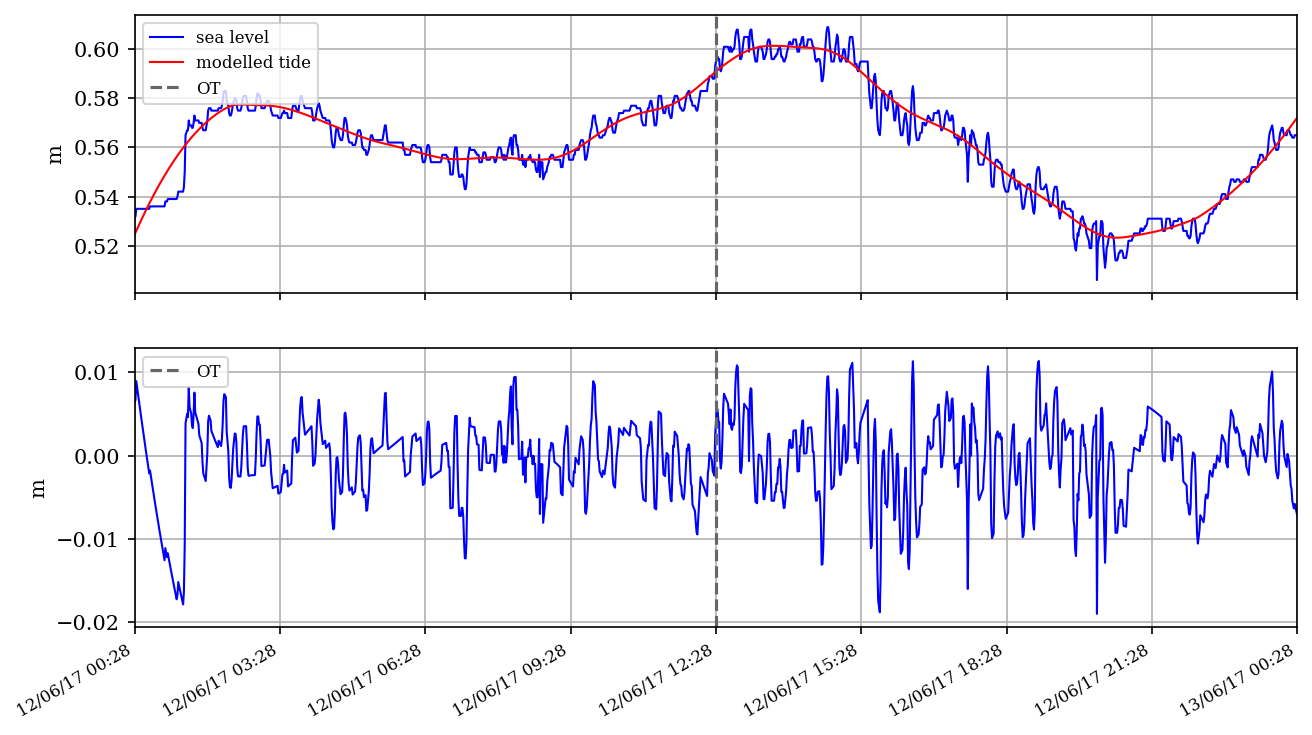

Supplement: Supplementary file 6 — Supplementary Dataset 3. Sea-level records. [file 41467_2021_25815_MOESM6_ESM.zip › sea_level_records/2017-06-12_Lesvos/syro.pr1.rmn.png]

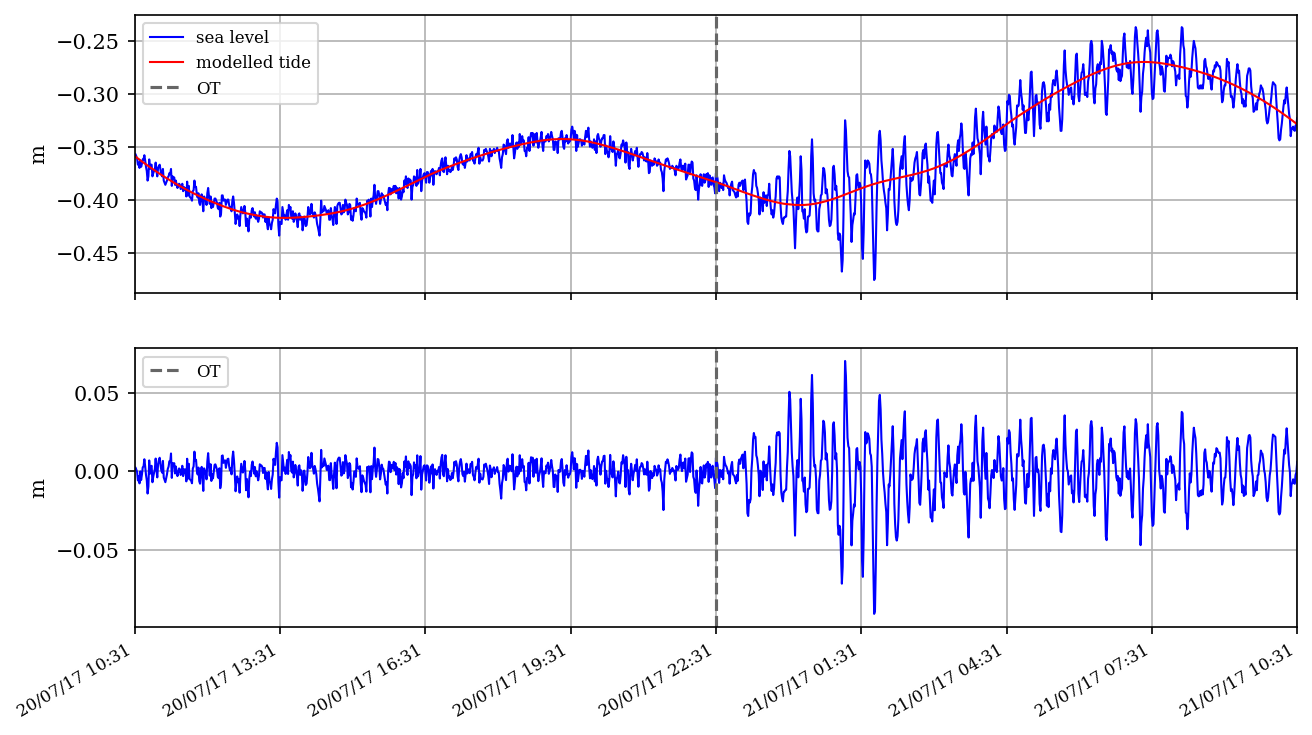

Supplement: Supplementary file 6 — Supplementary Dataset 3. Sea-level records. [file 41467_2021_25815_MOESM6_ESM.zip › sea_level_records/2017-07-20_Kos-Bodrum/NOA03.rad.rmn.png]

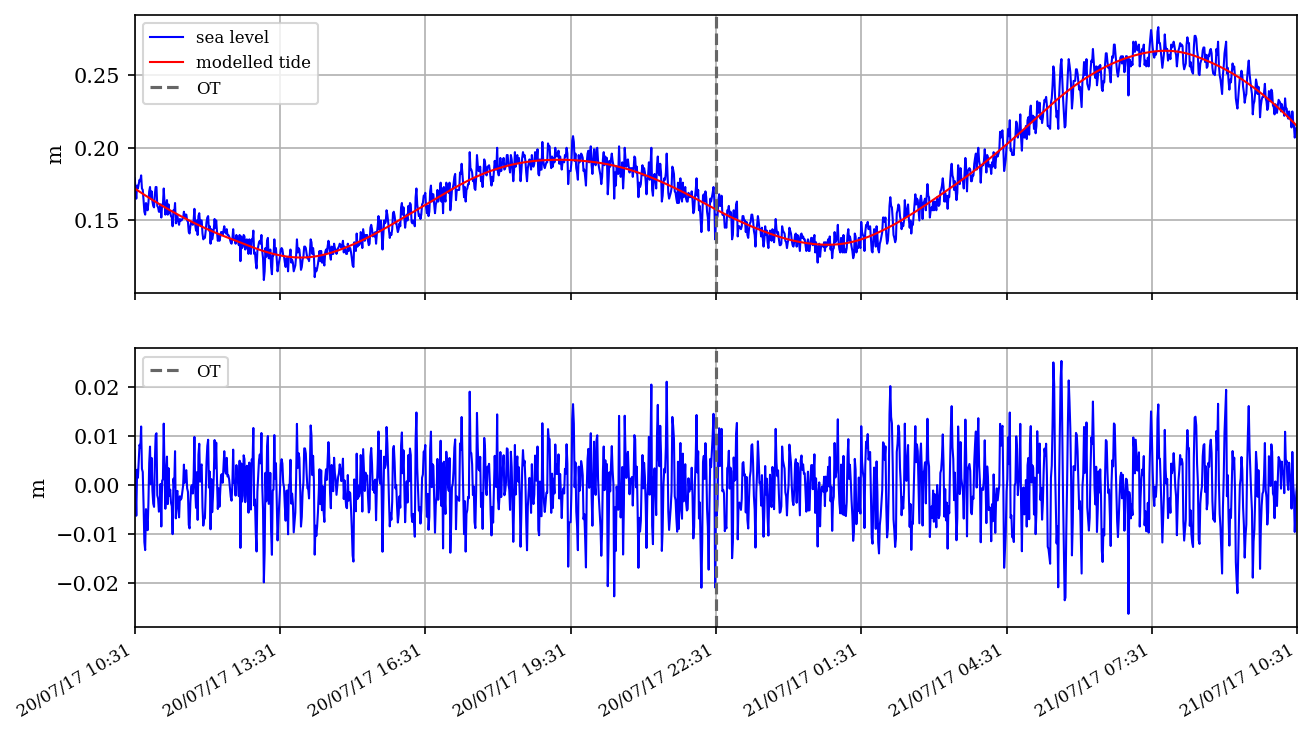

Supplement: Supplementary file 6 — Supplementary Dataset 3. Sea-level records. [file 41467_2021_25815_MOESM6_ESM.zip › sea_level_records/2017-07-20_Kos-Bodrum/NOA04.rad.rmn.png]

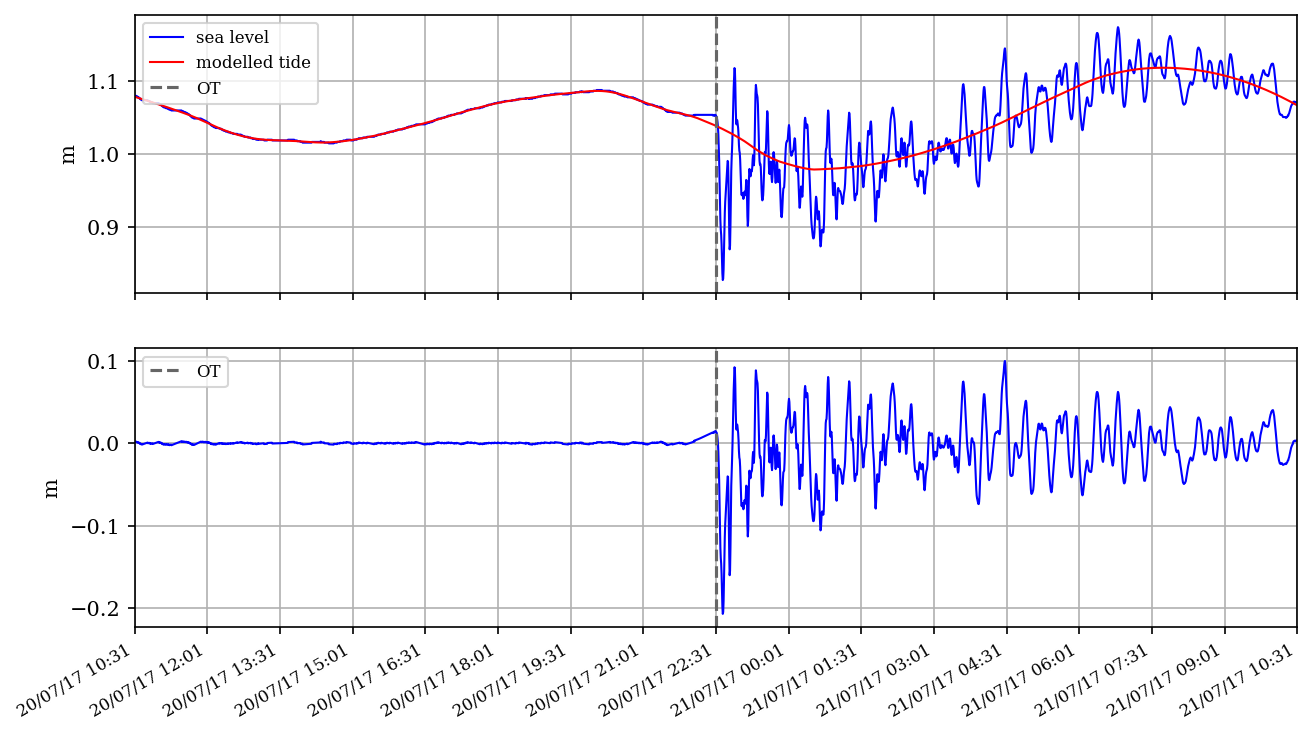

Supplement: Supplementary file 6 — Supplementary Dataset 3. Sea-level records. [file 41467_2021_25815_MOESM6_ESM.zip › sea_level_records/2017-07-20_Kos-Bodrum/bodru.rad.rmn.png]

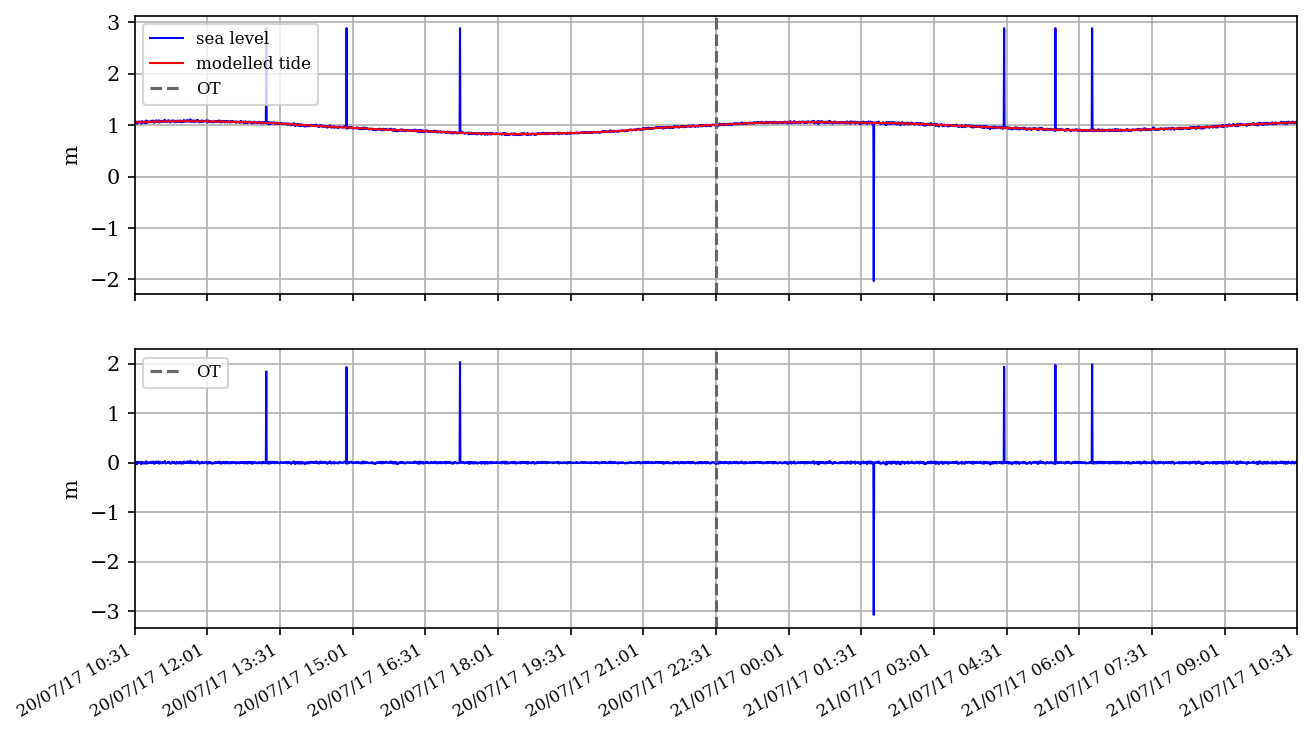

Supplement: Supplementary file 6 — Supplementary Dataset 3. Sea-level records. [file 41467_2021_25815_MOESM6_ESM.zip › sea_level_records/2017-07-20_Kos-Bodrum/gokce.rad.rmn.png]

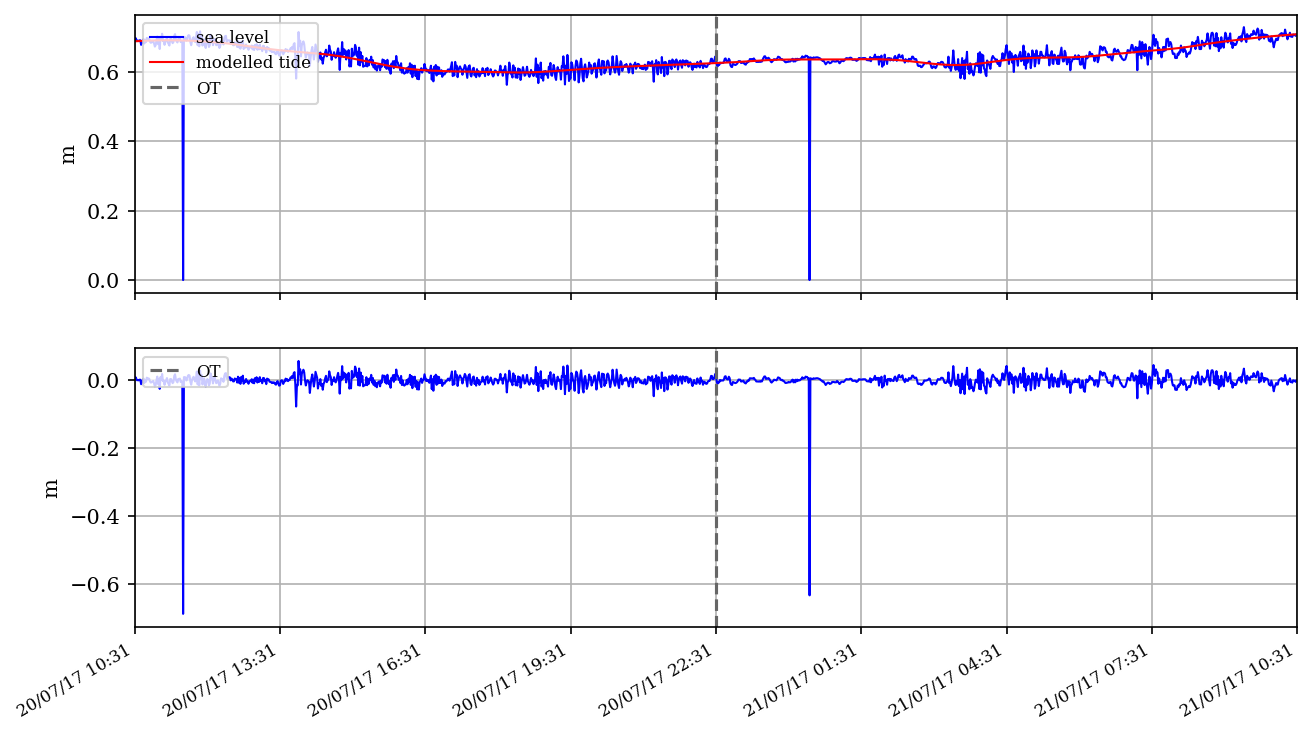

Supplement: Supplementary file 6 — Supplementary Dataset 3. Sea-level records. [file 41467_2021_25815_MOESM6_ESM.zip › sea_level_records/2017-07-20_Kos-Bodrum/peir.pr1.rmn.png]

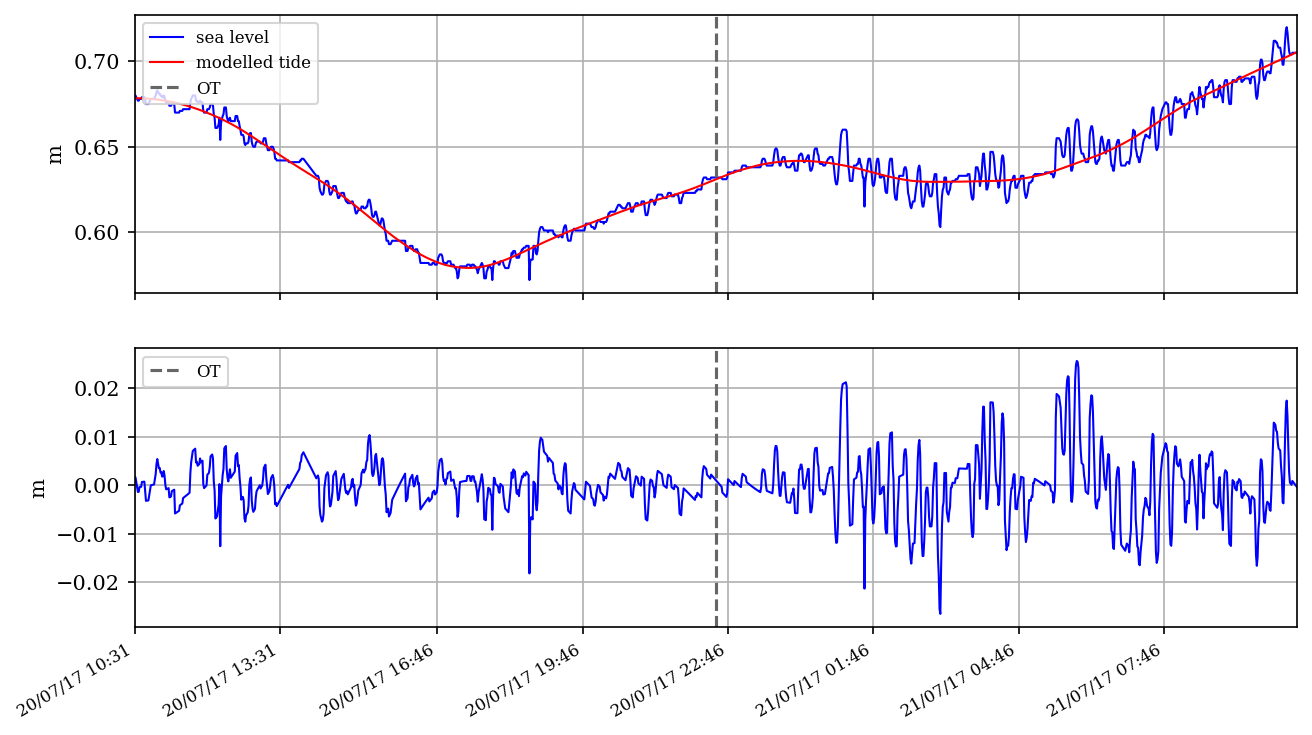

Supplement: Supplementary file 6 — Supplementary Dataset 3. Sea-level records. [file 41467_2021_25815_MOESM6_ESM.zip › sea_level_records/2017-07-20_Kos-Bodrum/syro.pr1.rmn.png]

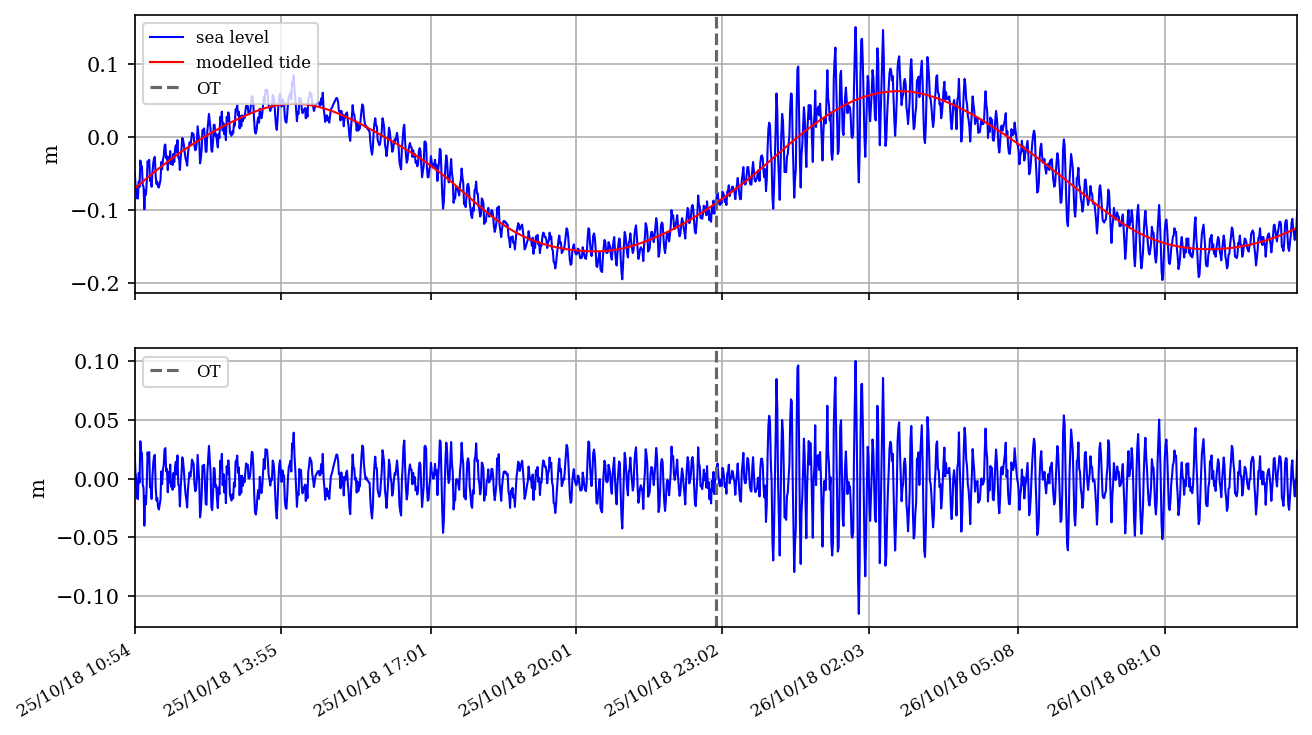

Supplement: Supplementary file 6 — Supplementary Dataset 3. Sea-level records. [file 41467_2021_25815_MOESM6_ESM.zip › sea_level_records/2018-10-25_Zakynthos/CR08.rad.rmn.png]

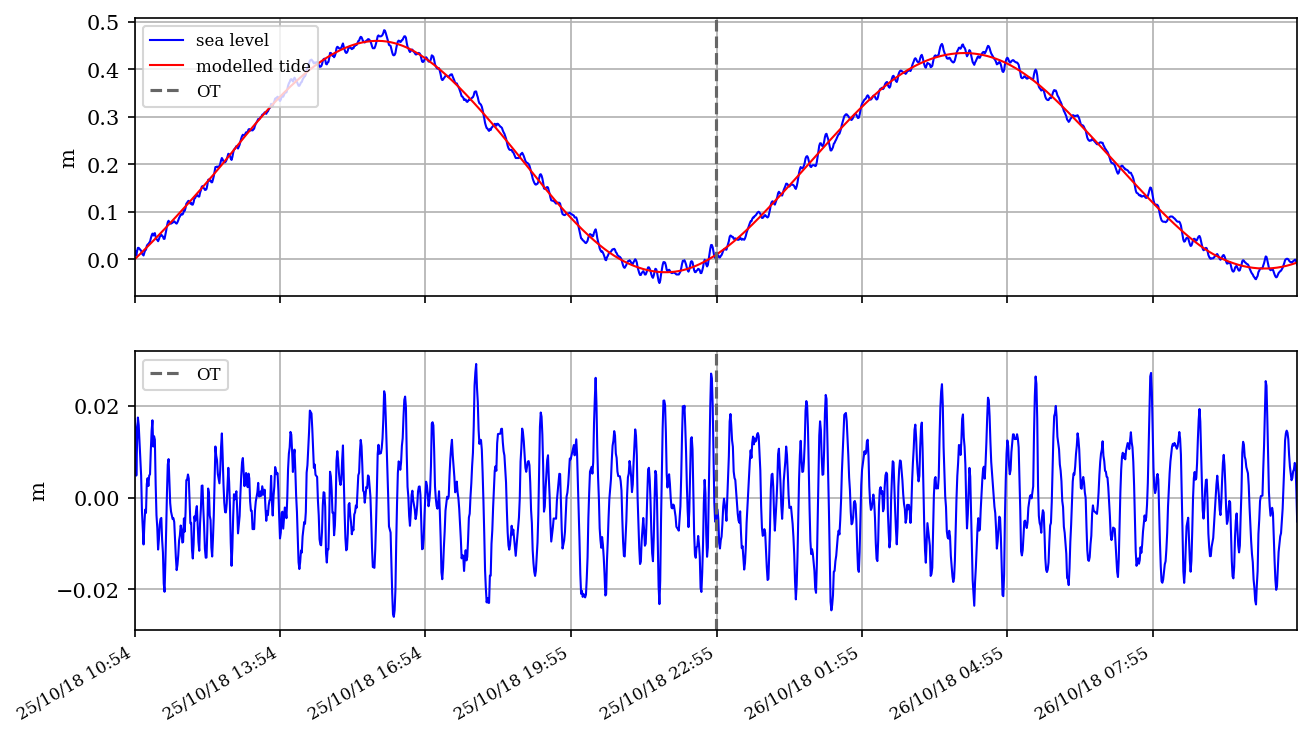

Supplement: Supplementary file 6 — Supplementary Dataset 3. Sea-level records. [file 41467_2021_25815_MOESM6_ESM.zip › sea_level_records/2018-10-25_Zakynthos/NOA05.rad.rmn.png]

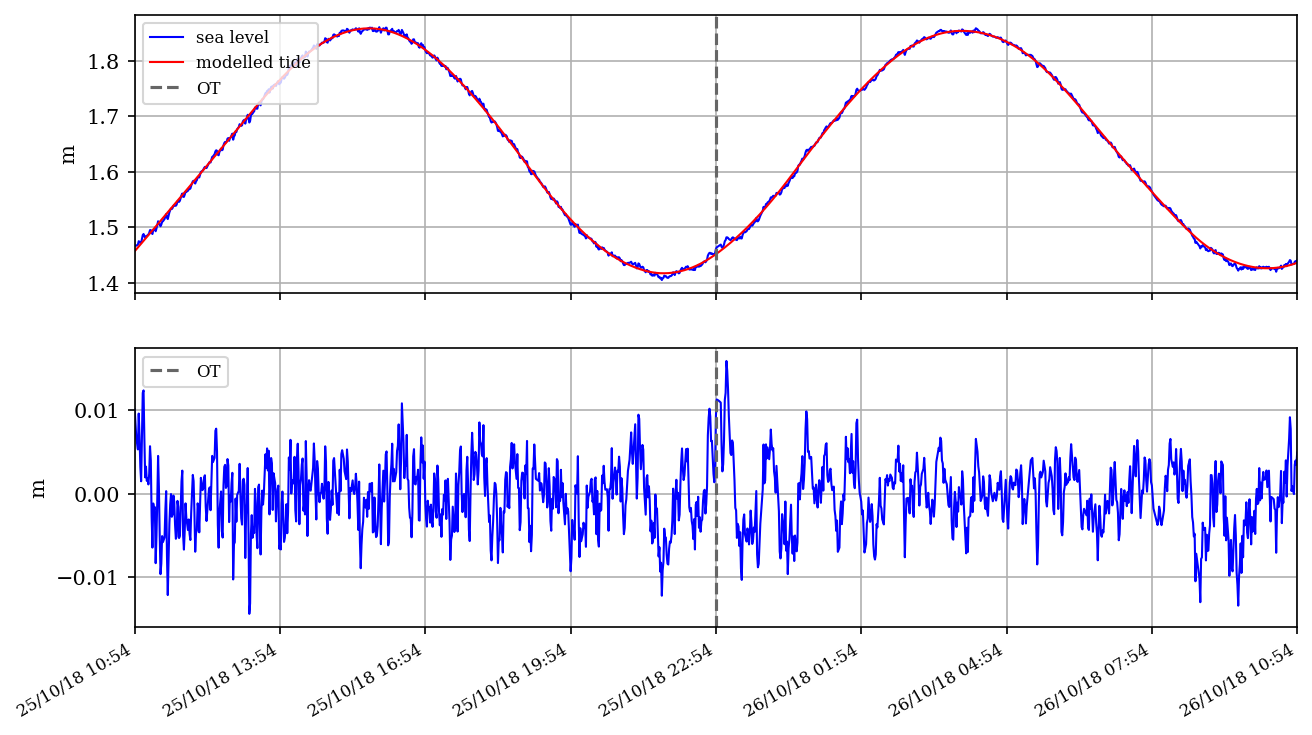

Supplement: Supplementary file 6 — Supplementary Dataset 3. Sea-level records. [file 41467_2021_25815_MOESM6_ESM.zip › sea_level_records/2018-10-25_Zakynthos/NOA06.rad.rmn.png]

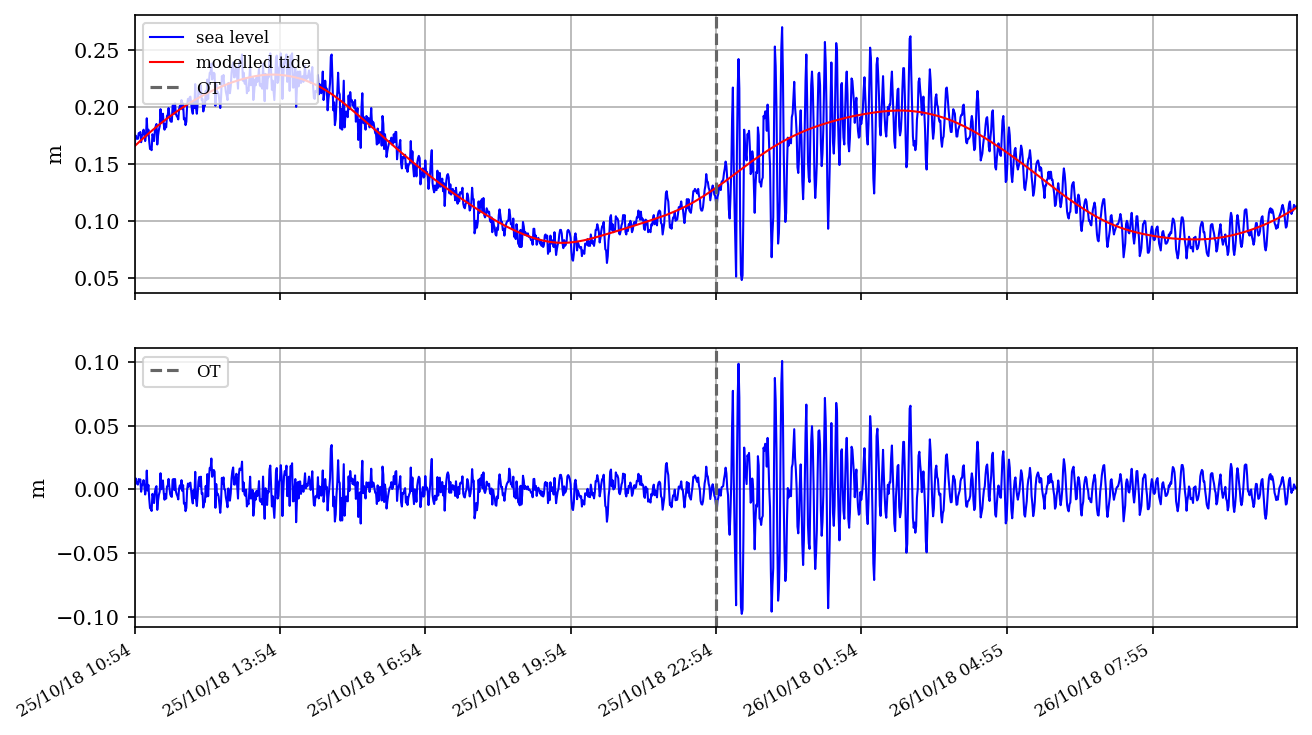

Supplement: Supplementary file 6 — Supplementary Dataset 3. Sea-level records. [file 41467_2021_25815_MOESM6_ESM.zip › sea_level_records/2018-10-25_Zakynthos/NOA08.rad.rmn.png]

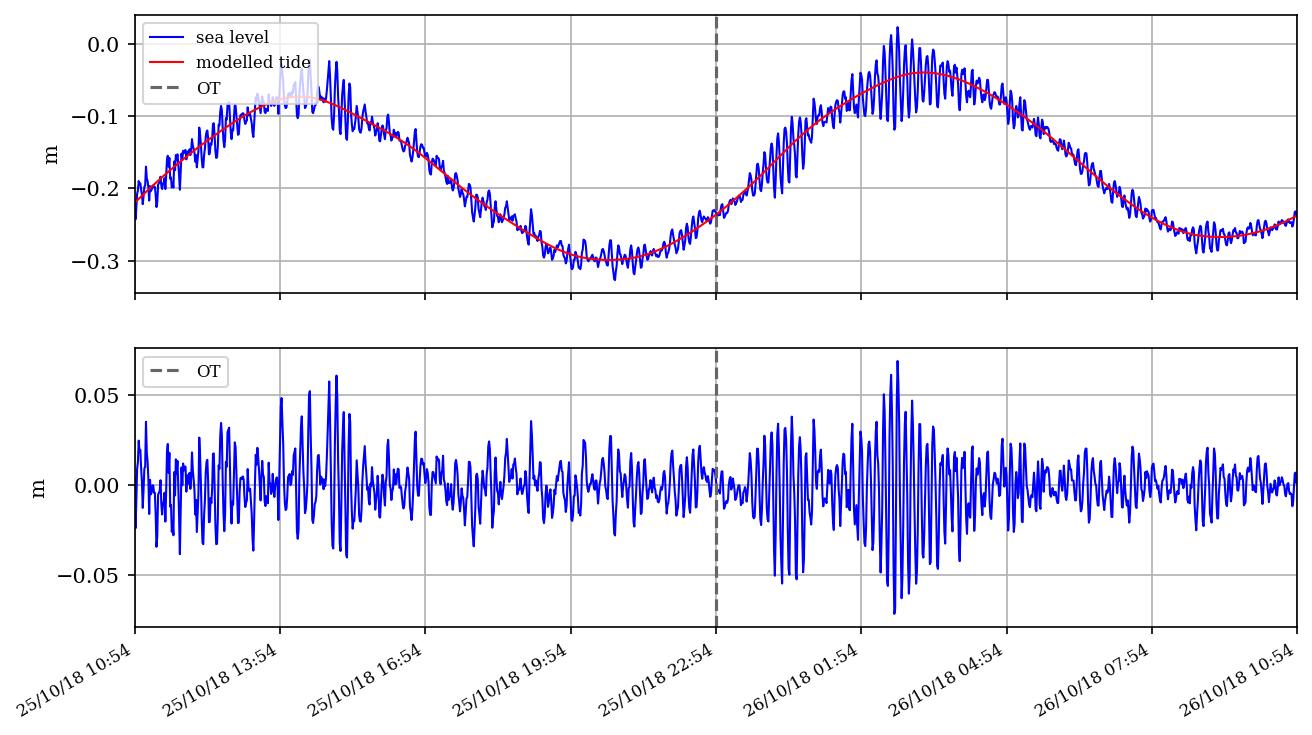

Supplement: Supplementary file 6 — Supplementary Dataset 3. Sea-level records. [file 41467_2021_25815_MOESM6_ESM.zip › sea_level_records/2018-10-25_Zakynthos/OT15.rad.rmn.png]

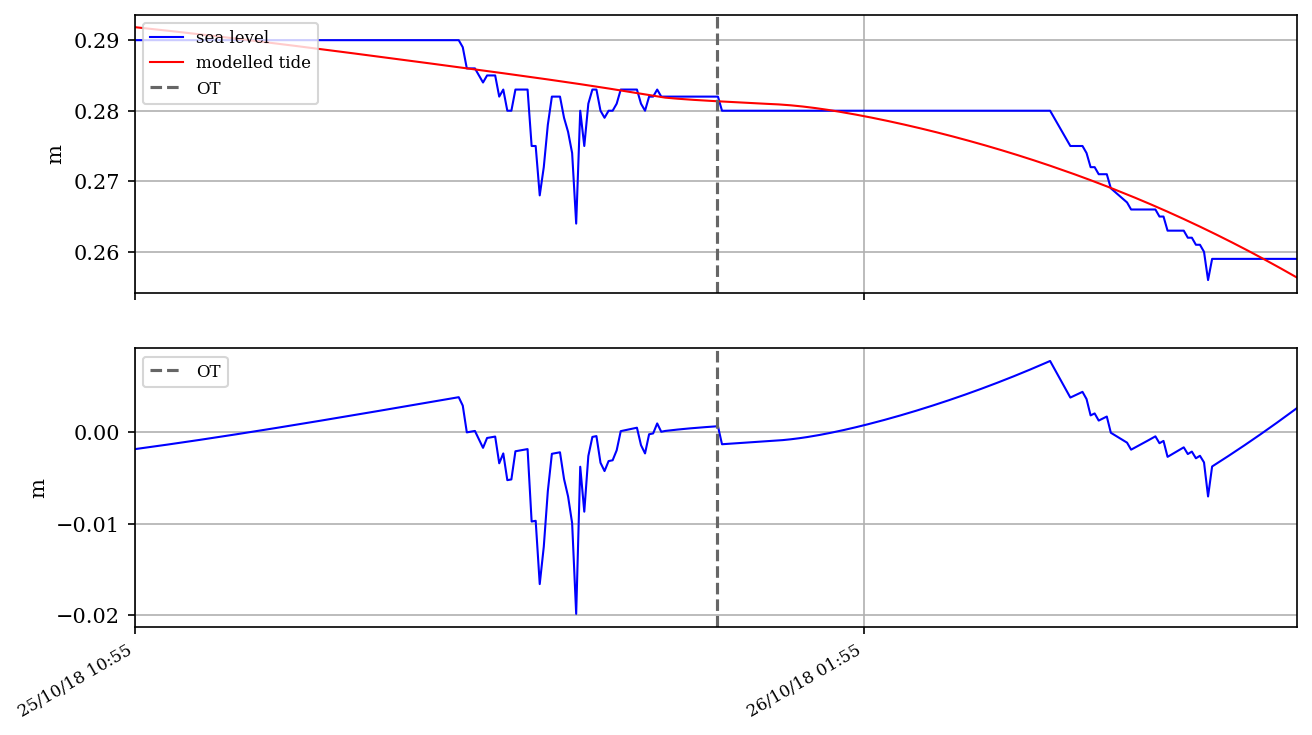

Supplement: Supplementary file 6 — Supplementary Dataset 3. Sea-level records. [file 41467_2021_25815_MOESM6_ESM.zip › sea_level_records/2018-10-25_Zakynthos/kala.pr1.rmn.png]

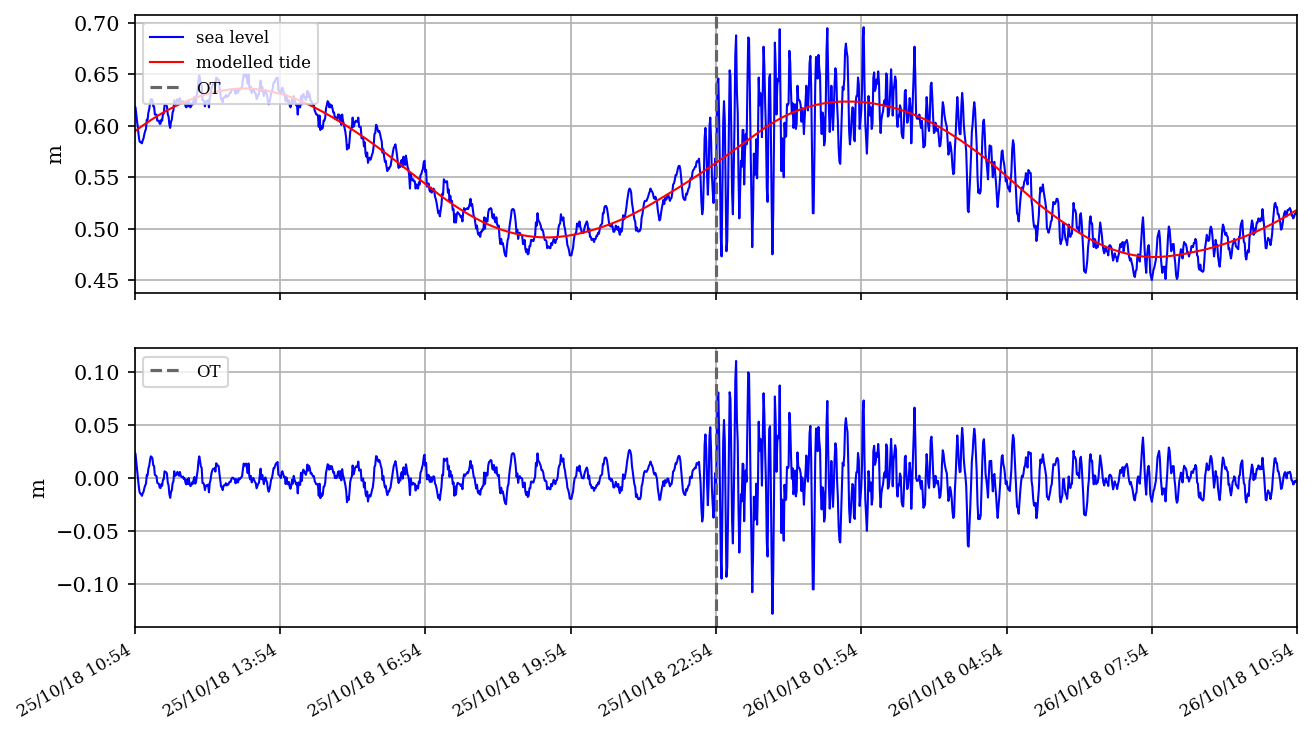

Supplement: Supplementary file 6 — Supplementary Dataset 3. Sea-level records. [file 41467_2021_25815_MOESM6_ESM.zip › sea_level_records/2018-10-25_Zakynthos/kata.pr1.rmn.png]

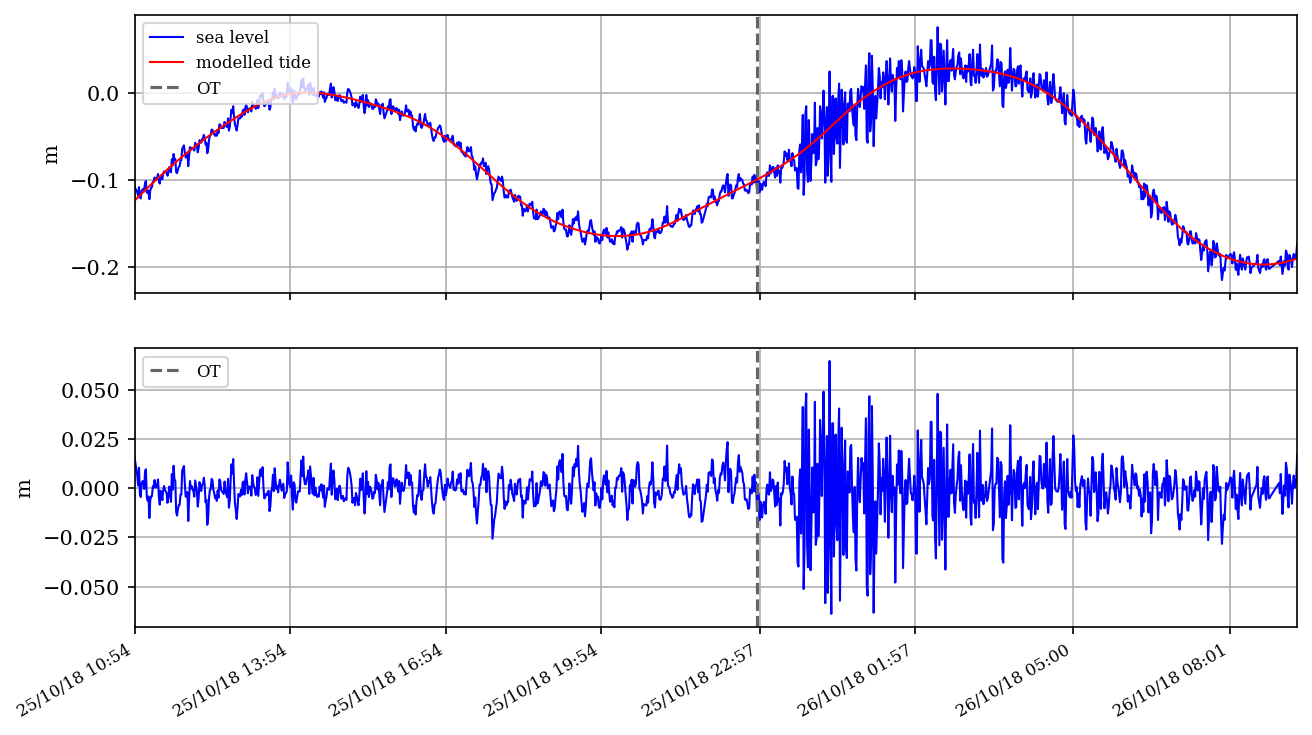

Supplement: Supplementary file 6 — Supplementary Dataset 3. Sea-level records. [file 41467_2021_25815_MOESM6_ESM.zip › sea_level_records/2018-10-25_Zakynthos/lcst.rad.rmn.png]

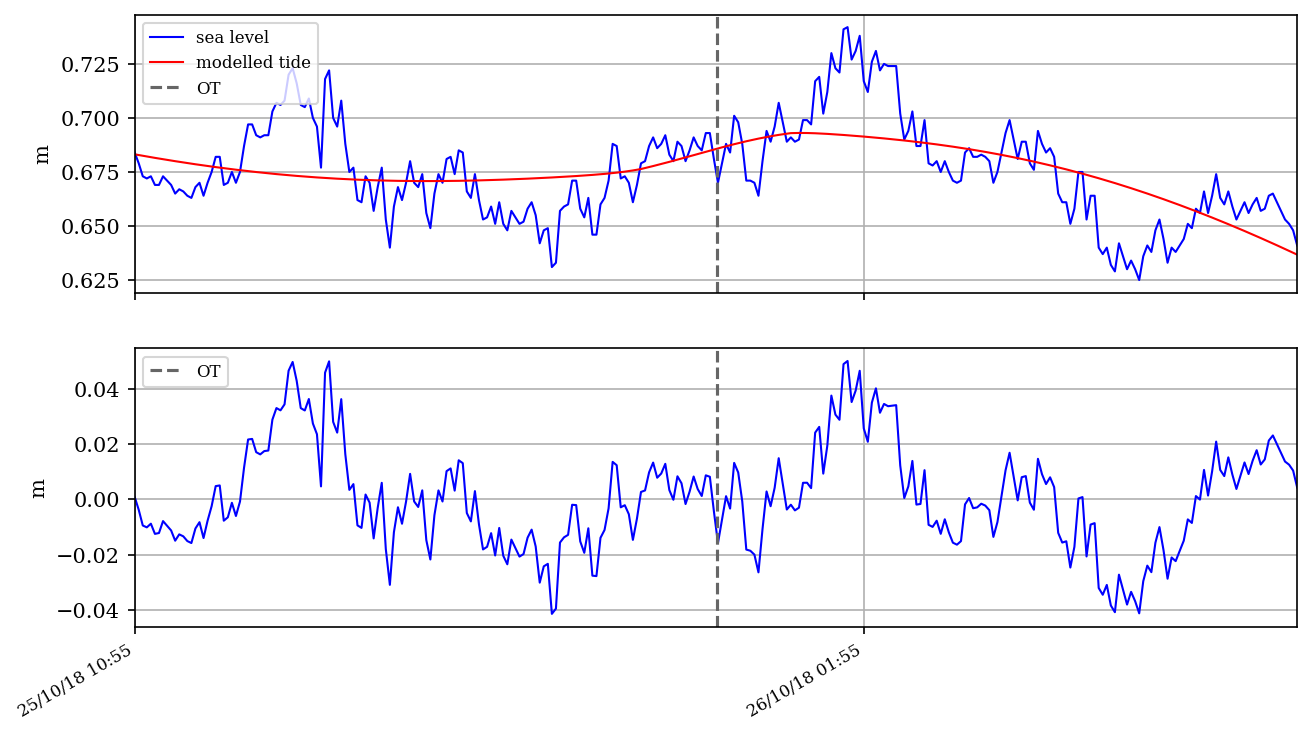

Supplement: Supplementary file 6 — Supplementary Dataset 3. Sea-level records. [file 41467_2021_25815_MOESM6_ESM.zip › sea_level_records/2018-10-25_Zakynthos/peir.pr1.rmn.png]

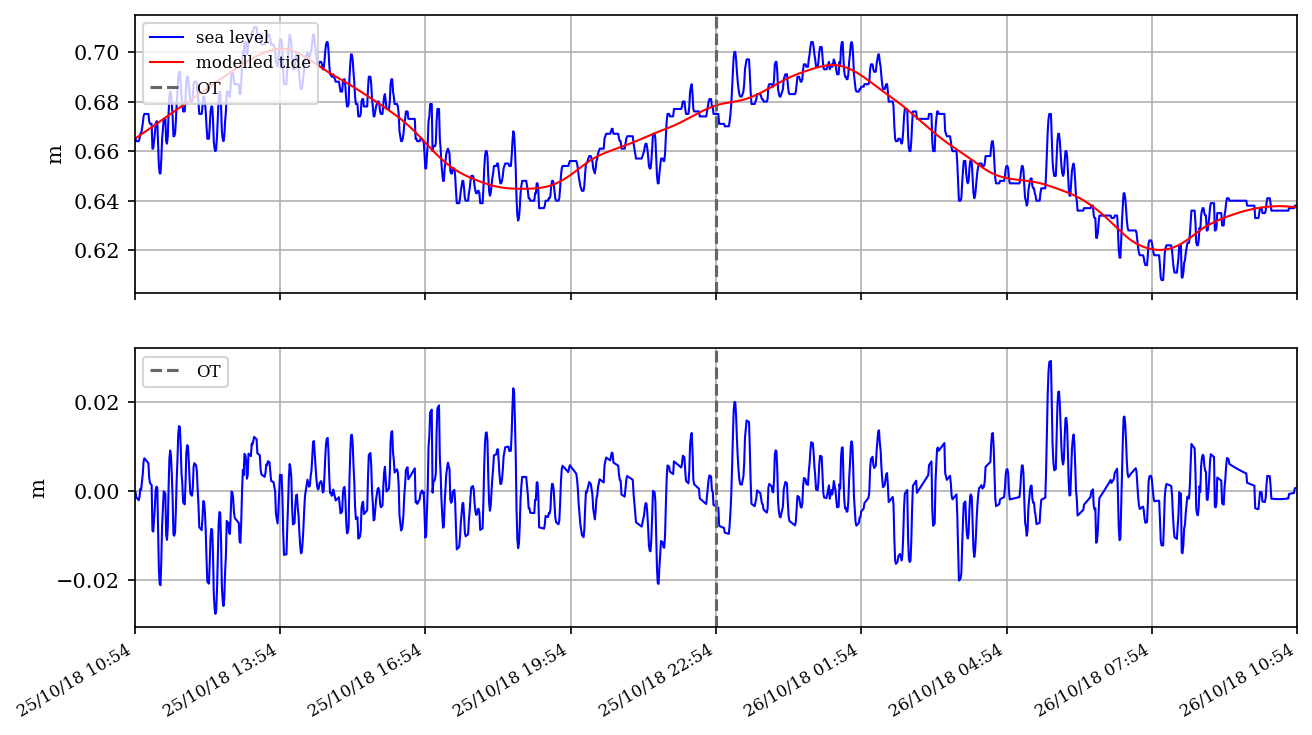

Supplement: Supplementary file 6 — Supplementary Dataset 3. Sea-level records. [file 41467_2021_25815_MOESM6_ESM.zip › sea_level_records/2018-10-25_Zakynthos/syro.pr1.rmn.png]

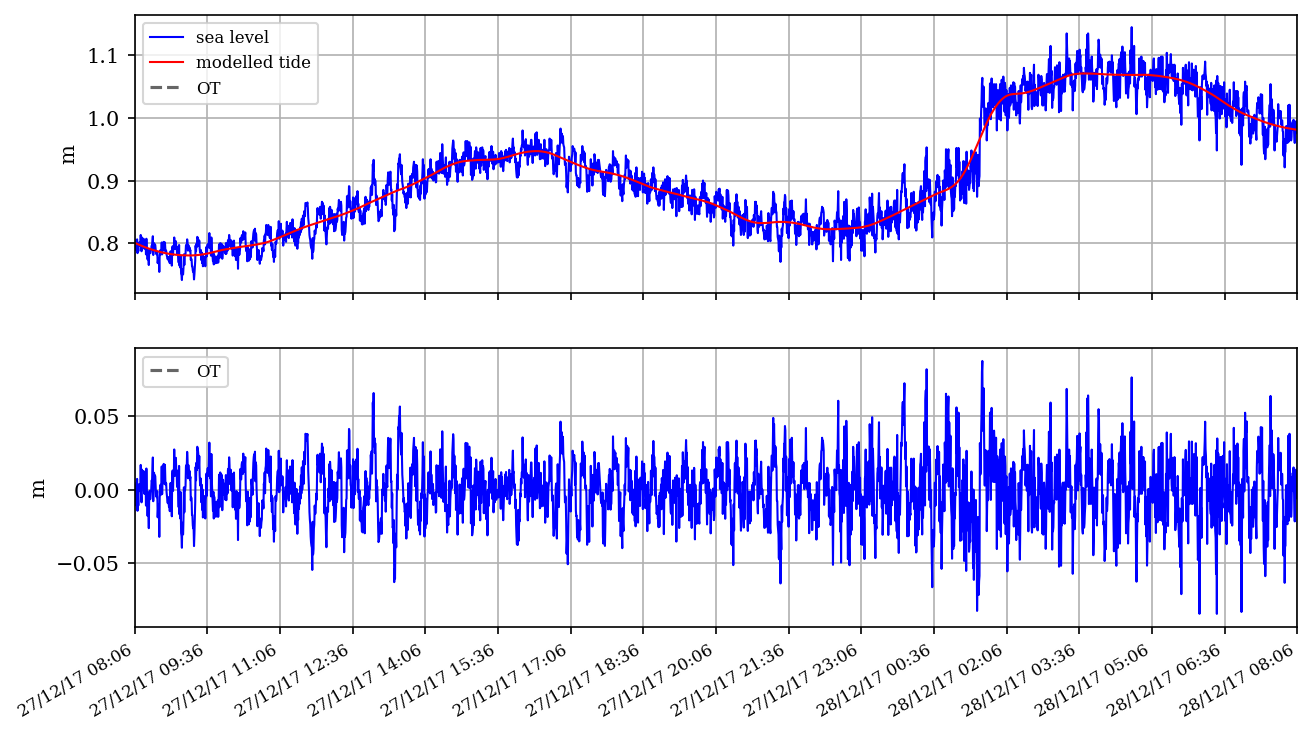

Supplement: Supplementary file 6 — Supplementary Dataset 3. Sea-level records. [file 41467_2021_25815_MOESM6_ESM.zip › sea_level_records/2019-03-20_Turkey/bozya.rad.rmn.png]

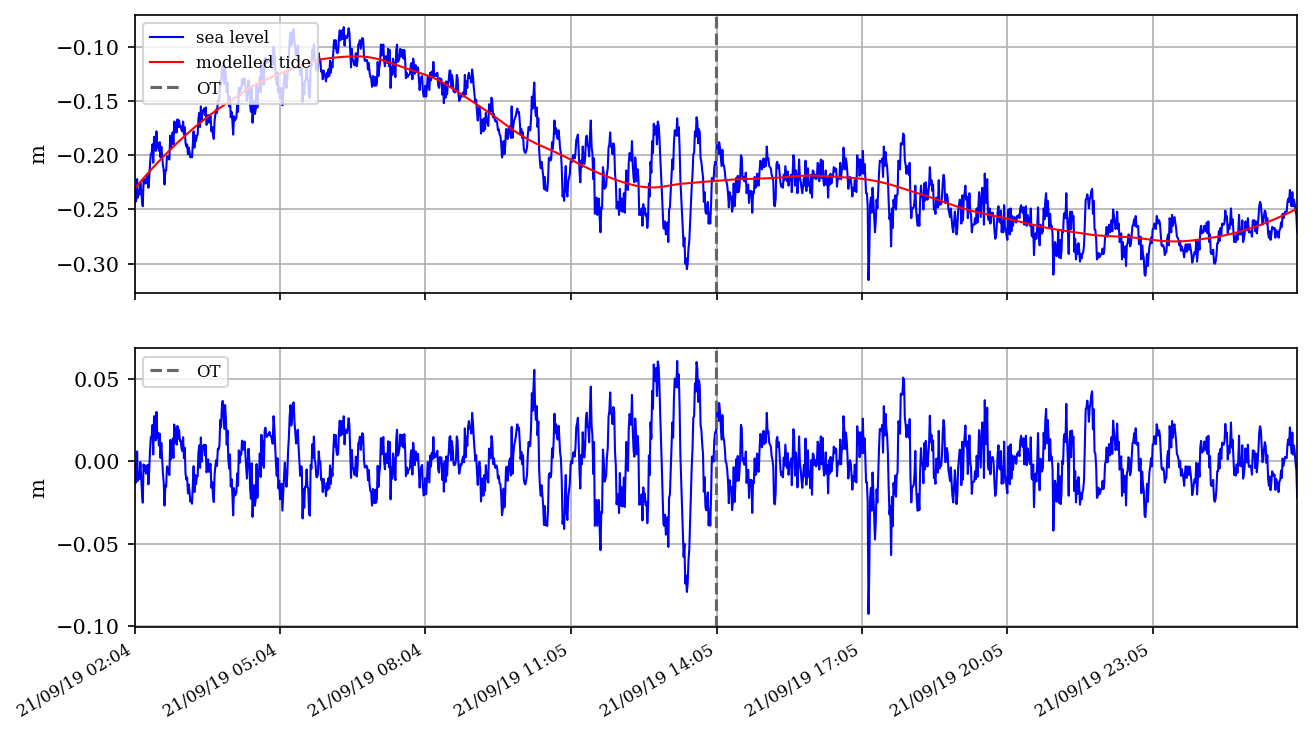

Supplement: Supplementary file 6 — Supplementary Dataset 3. Sea-level records. [file 41467_2021_25815_MOESM6_ESM.zip › sea_level_records/2019-09-21_Albania/BA05.rad.rmn.png]

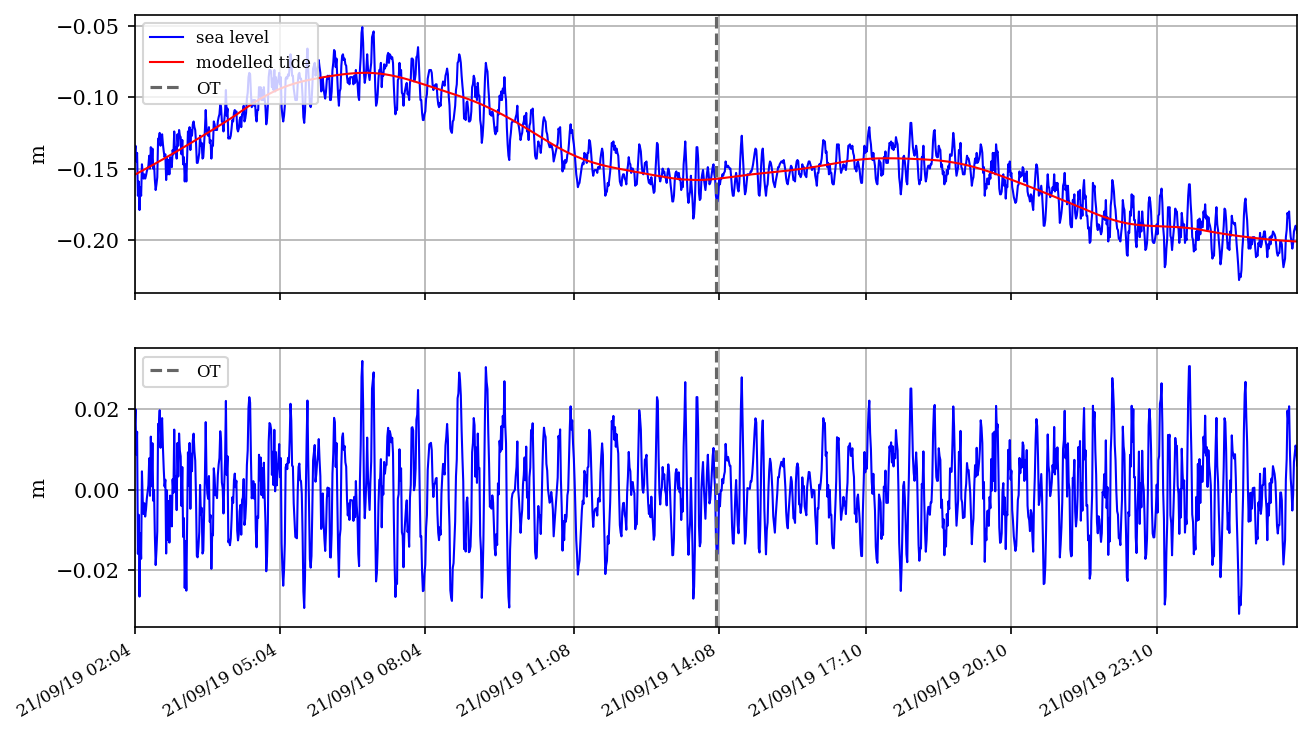

Supplement: Supplementary file 6 — Supplementary Dataset 3. Sea-level records. [file 41467_2021_25815_MOESM6_ESM.zip › sea_level_records/2019-09-21_Albania/CR08.rad.rmn.png]

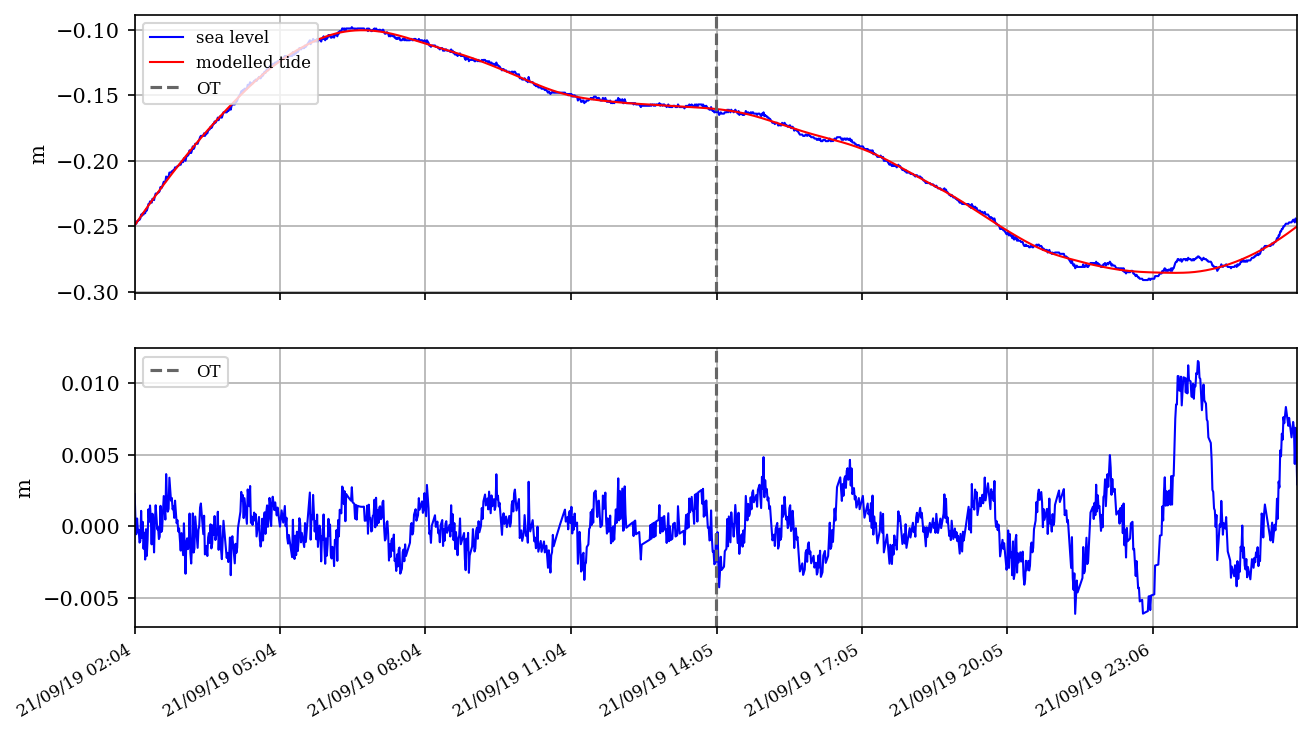

Supplement: Supplementary file 6 — Supplementary Dataset 3. Sea-level records. [file 41467_2021_25815_MOESM6_ESM.zip › sea_level_records/2019-09-21_Albania/IT45.rad.rmn.png]

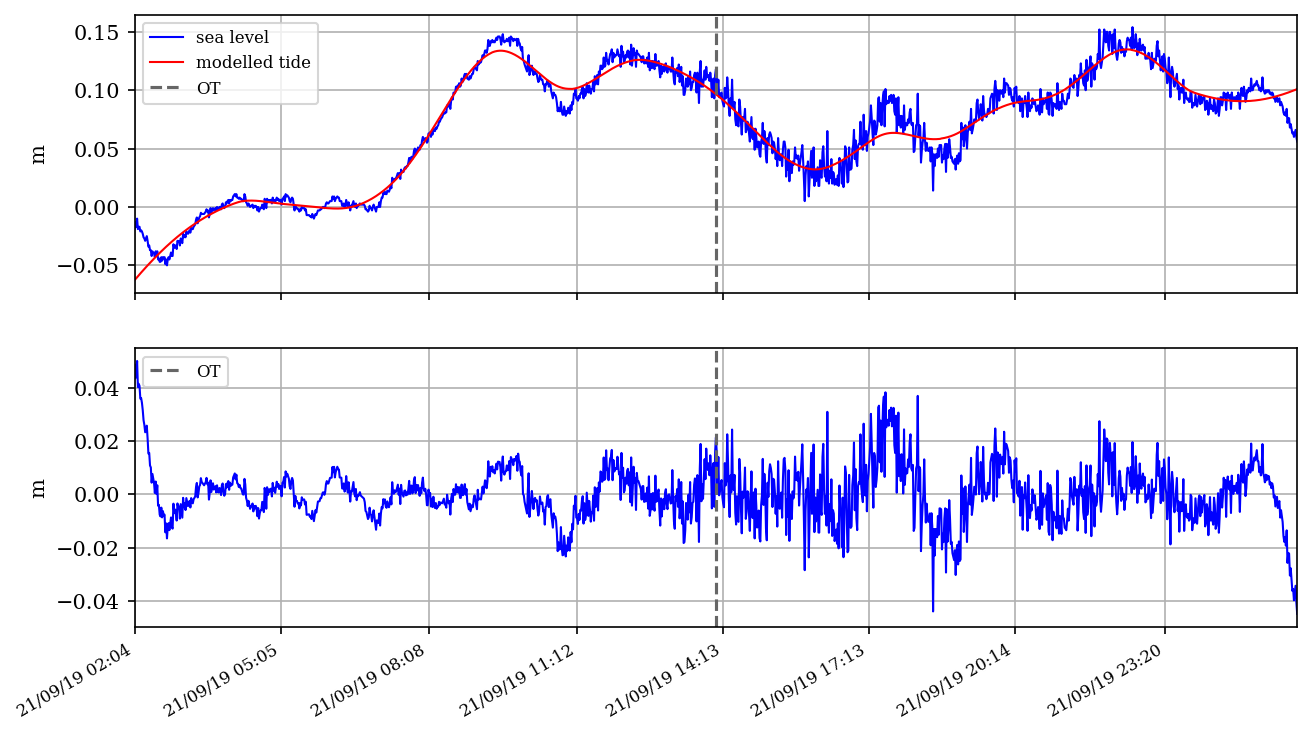

Supplement: Supplementary file 6 — Supplementary Dataset 3. Sea-level records. [file 41467_2021_25815_MOESM6_ESM.zip › sea_level_records/2019-09-21_Albania/NOA12.rad.rmn.png]

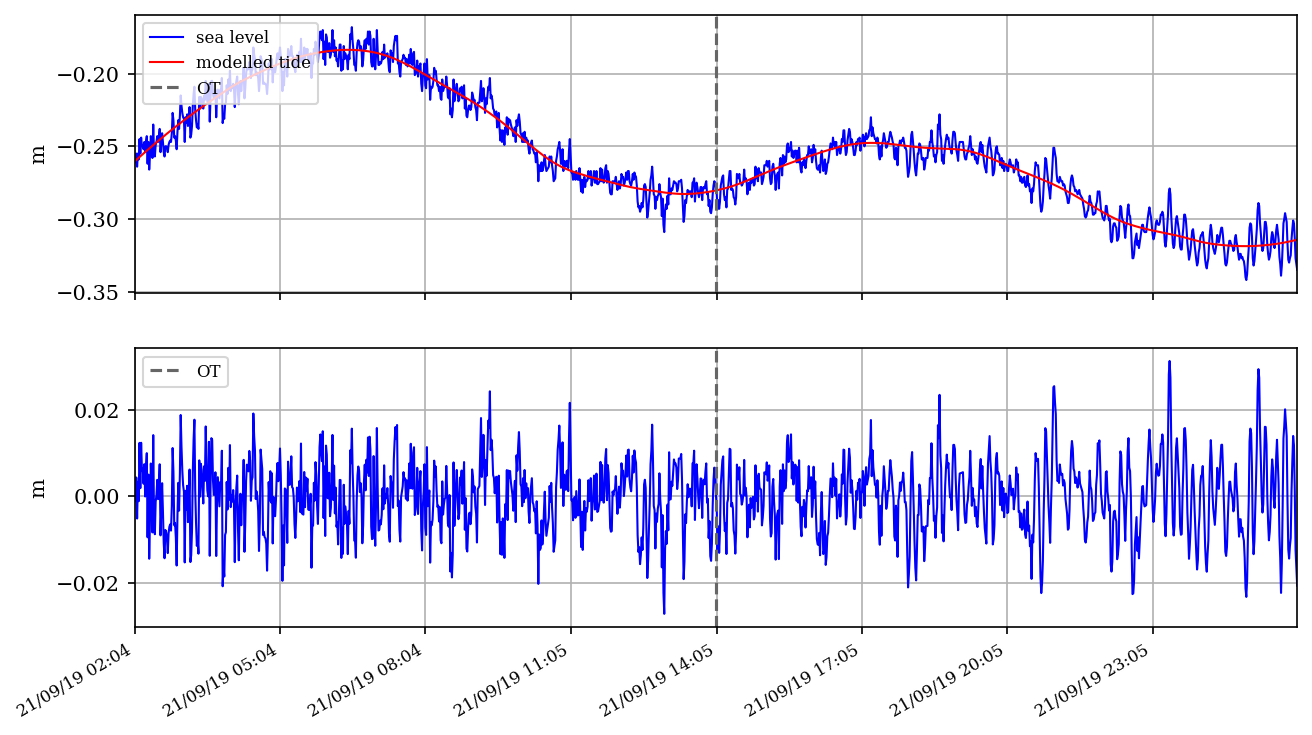

Supplement: Supplementary file 6 — Supplementary Dataset 3. Sea-level records. [file 41467_2021_25815_MOESM6_ESM.zip › sea_level_records/2019-09-21_Albania/OT15.rad.rmn.png]

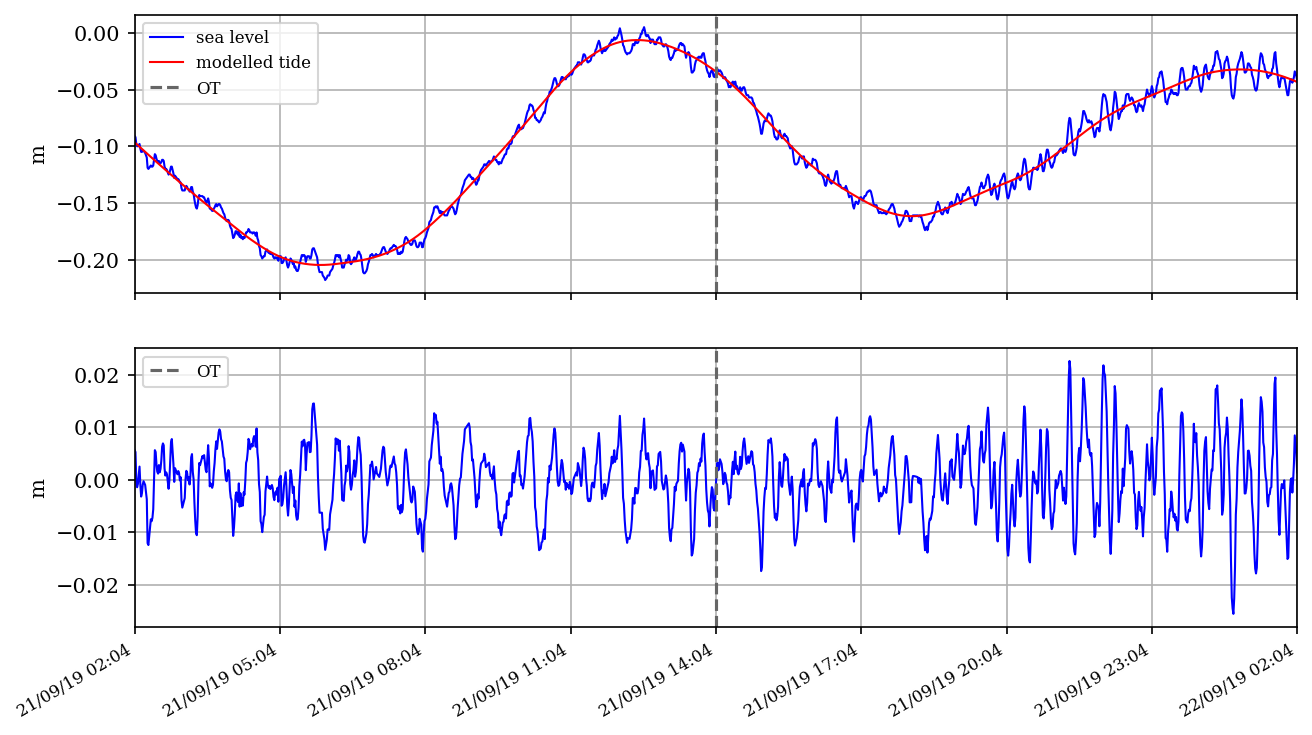

Supplement: Supplementary file 6 — Supplementary Dataset 3. Sea-level records. [file 41467_2021_25815_MOESM6_ESM.zip › sea_level_records/2019-09-21_Albania/PL14.rad.rmn.png]

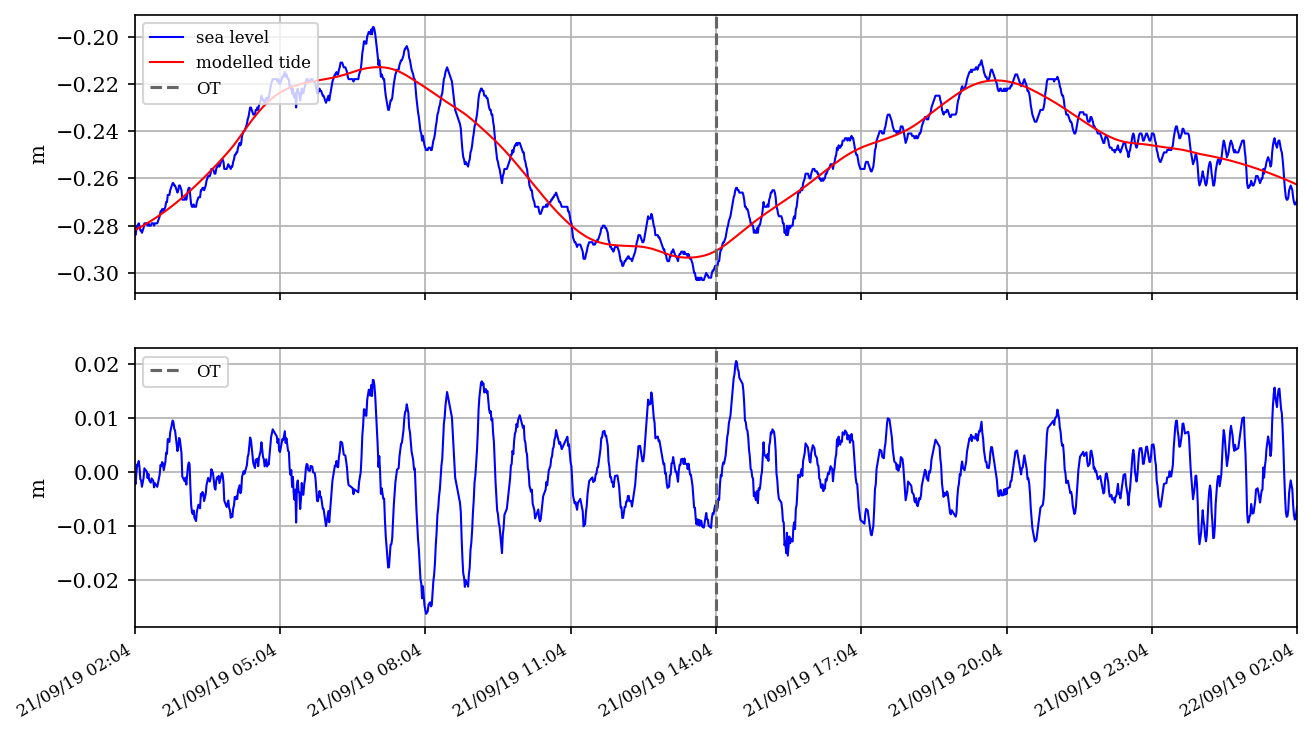

Supplement: Supplementary file 6 — Supplementary Dataset 3. Sea-level records. [file 41467_2021_25815_MOESM6_ESM.zip › sea_level_records/2019-09-21_Albania/TA18.rad.rmn.png]

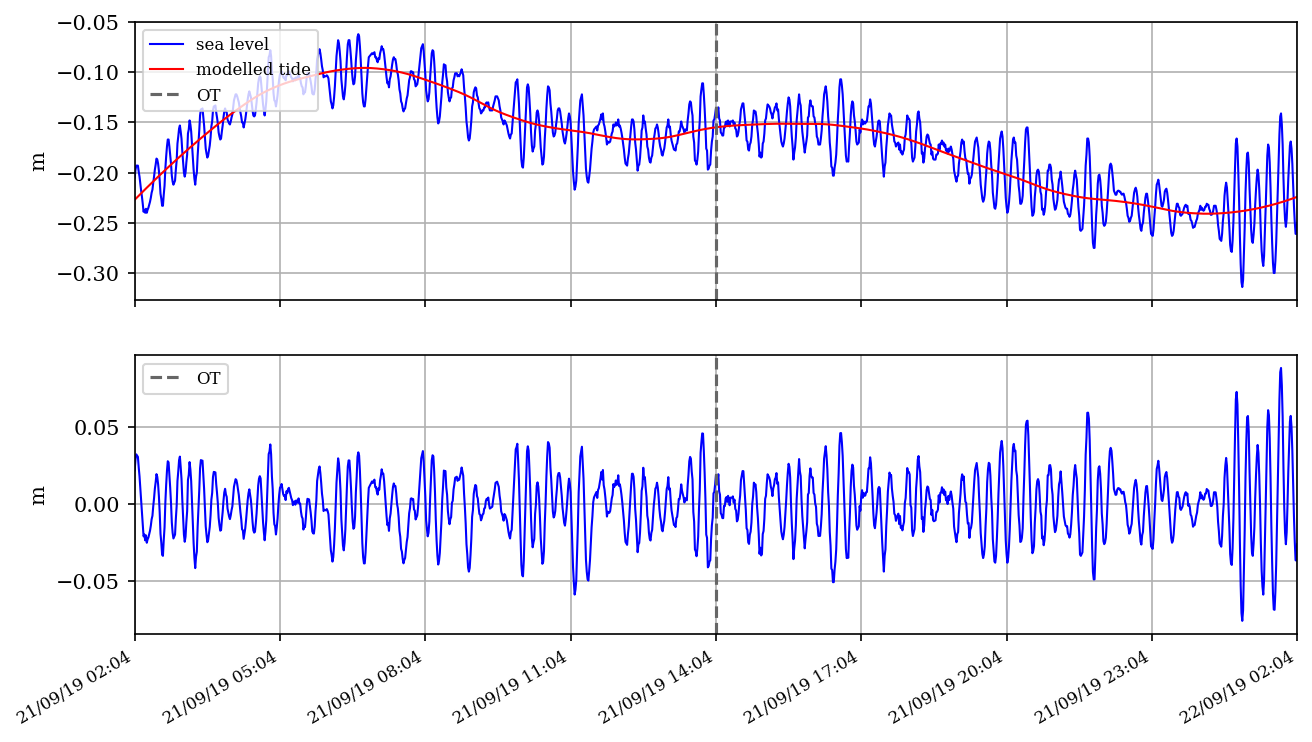

Supplement: Supplementary file 6 — Supplementary Dataset 3. Sea-level records. [file 41467_2021_25815_MOESM6_ESM.zip › sea_level_records/2019-09-21_Albania/VI12.rad.rmn.png]

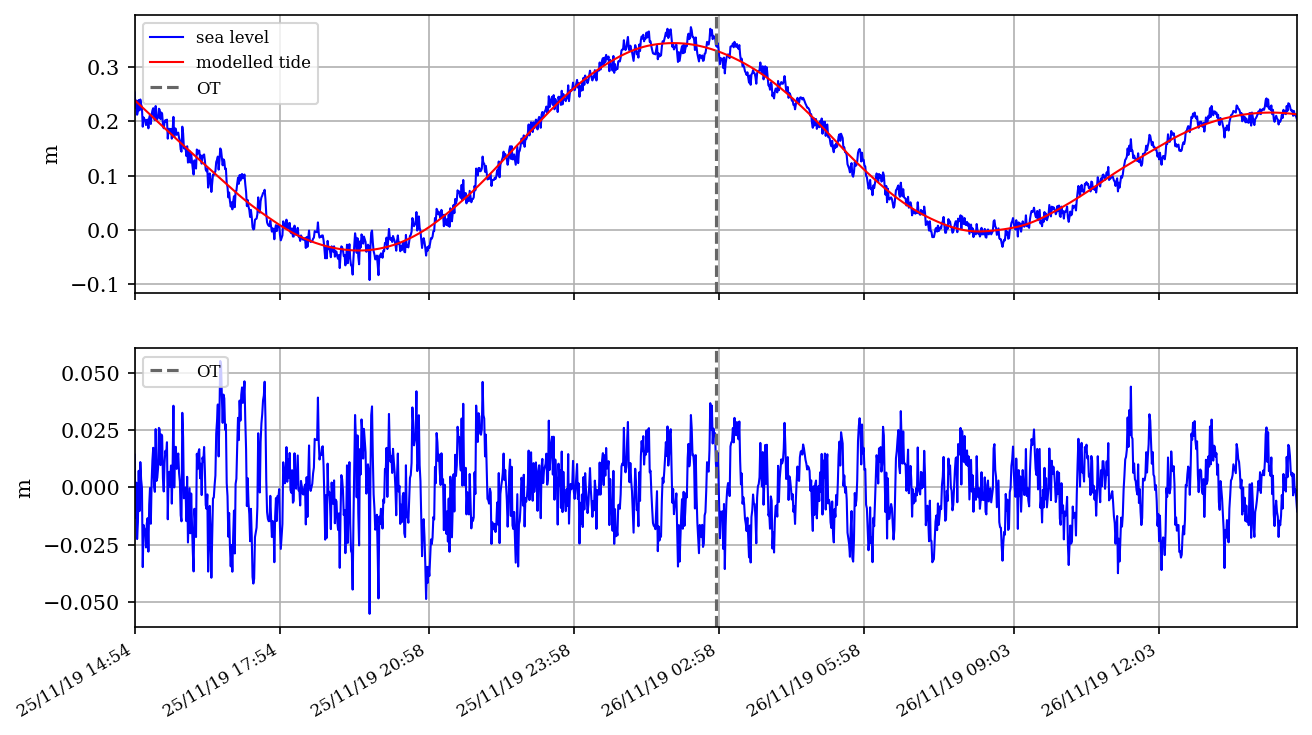

Supplement: Supplementary file 6 — Supplementary Dataset 3. Sea-level records. [file 41467_2021_25815_MOESM6_ESM.zip › sea_level_records/2019-11-26_Albania/BA05.rad.rmn.png]

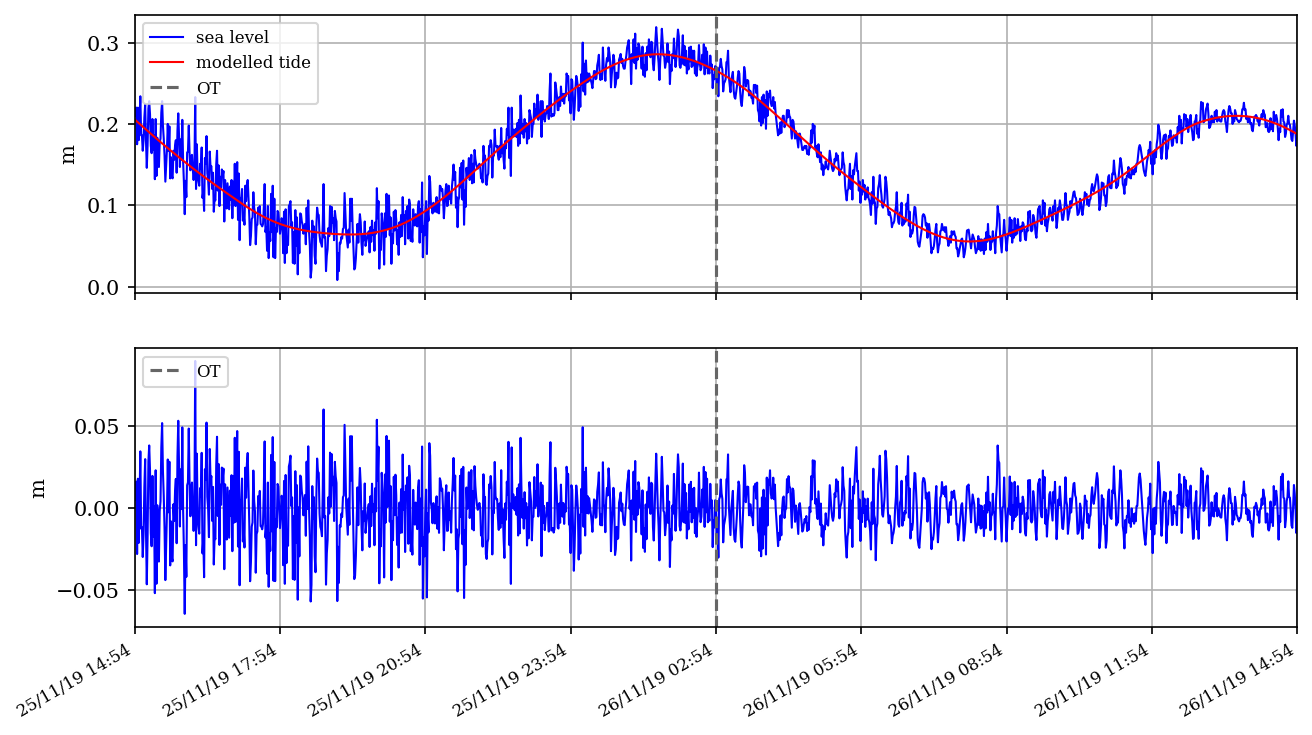

Supplement: Supplementary file 6 — Supplementary Dataset 3. Sea-level records. [file 41467_2021_25815_MOESM6_ESM.zip › sea_level_records/2019-11-26_Albania/CR08.rad.rmn.png]

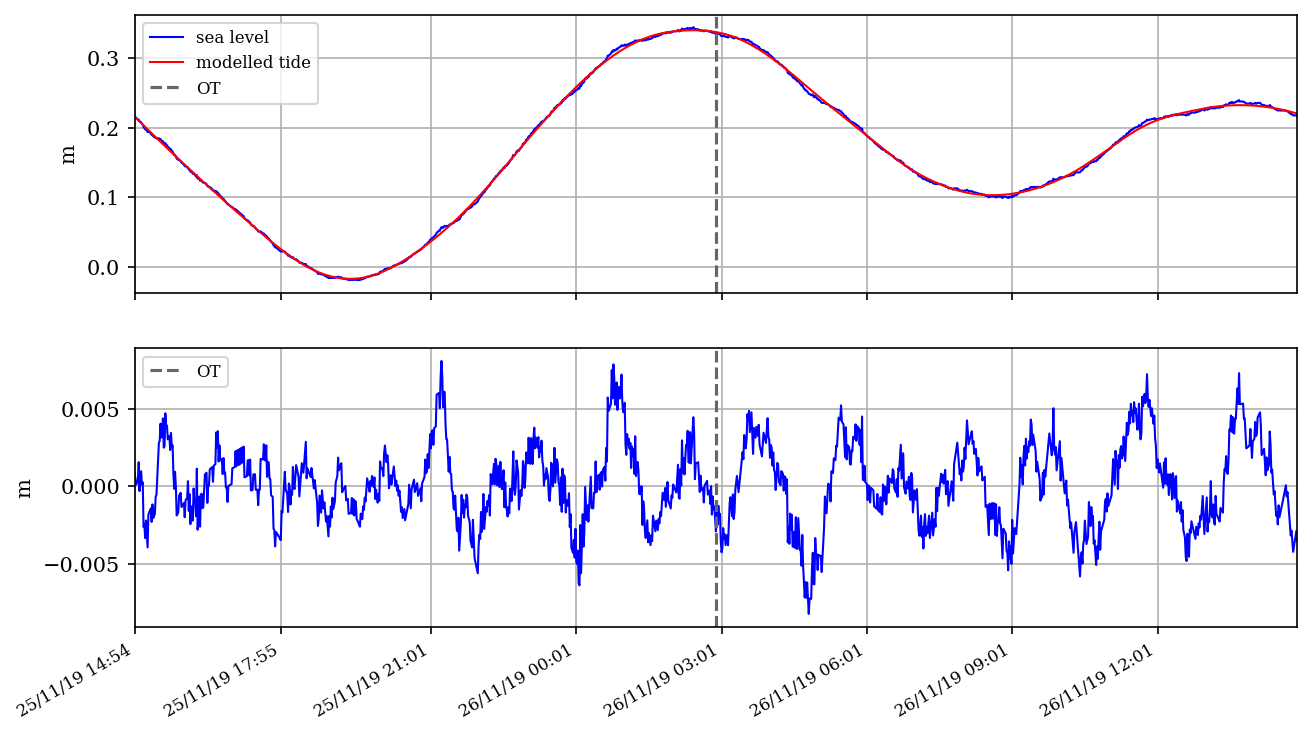

Supplement: Supplementary file 6 — Supplementary Dataset 3. Sea-level records. [file 41467_2021_25815_MOESM6_ESM.zip › sea_level_records/2019-11-26_Albania/IT45.rad.rmn.png]

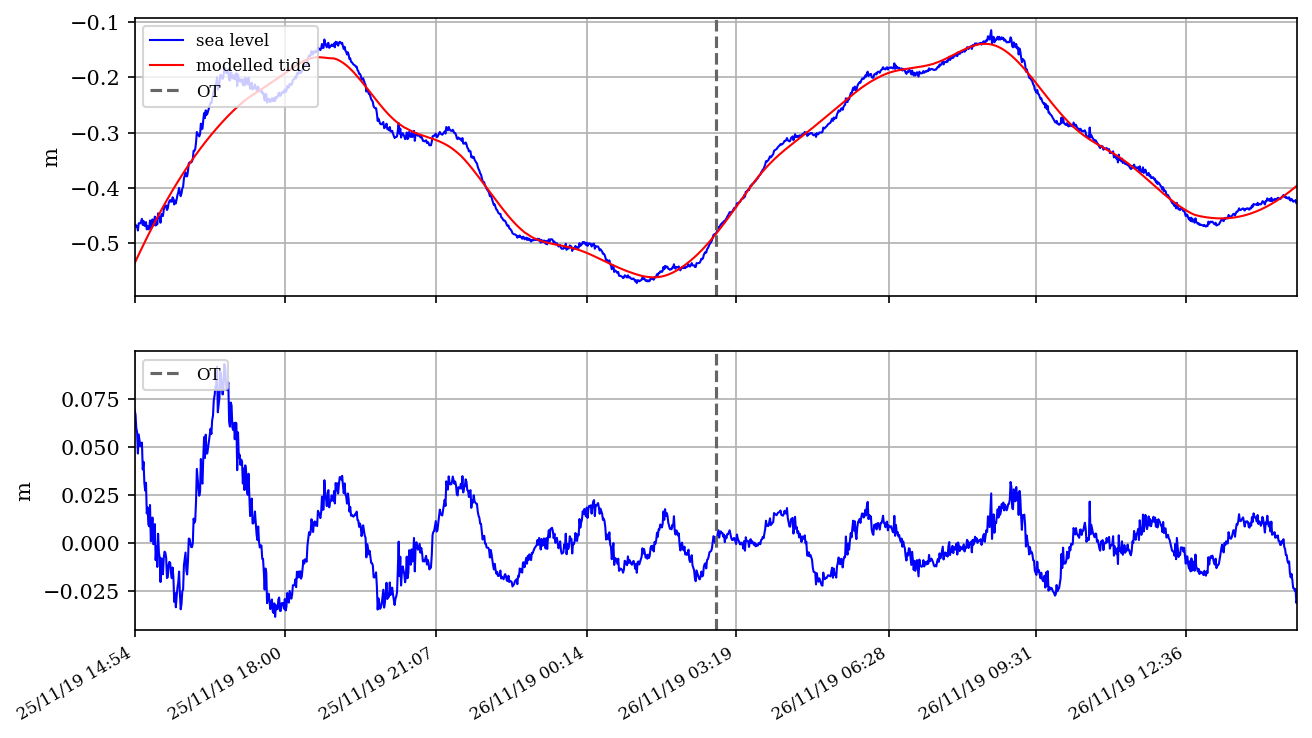

Supplement: Supplementary file 6 — Supplementary Dataset 3. Sea-level records. [file 41467_2021_25815_MOESM6_ESM.zip › sea_level_records/2019-11-26_Albania/NOA12.rad.rmn.png]

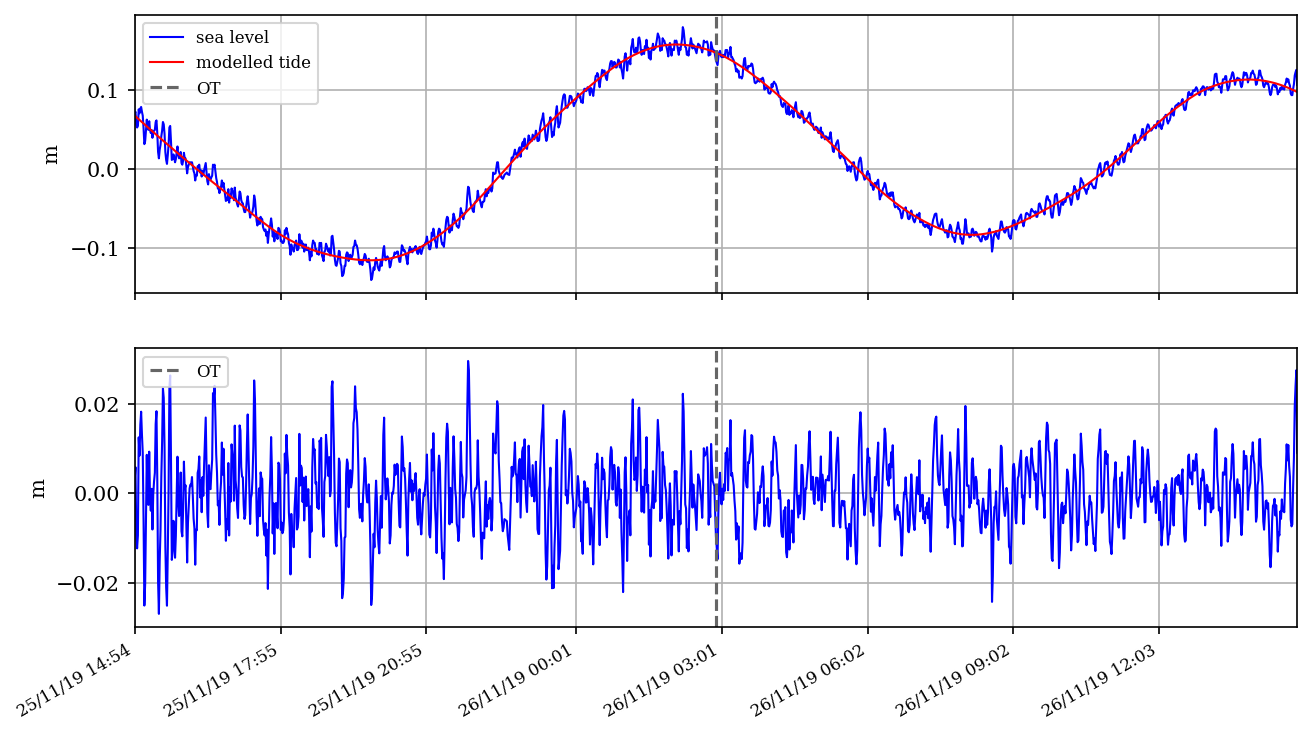

Supplement: Supplementary file 6 — Supplementary Dataset 3. Sea-level records. [file 41467_2021_25815_MOESM6_ESM.zip › sea_level_records/2019-11-26_Albania/OT15.rad.rmn.png]

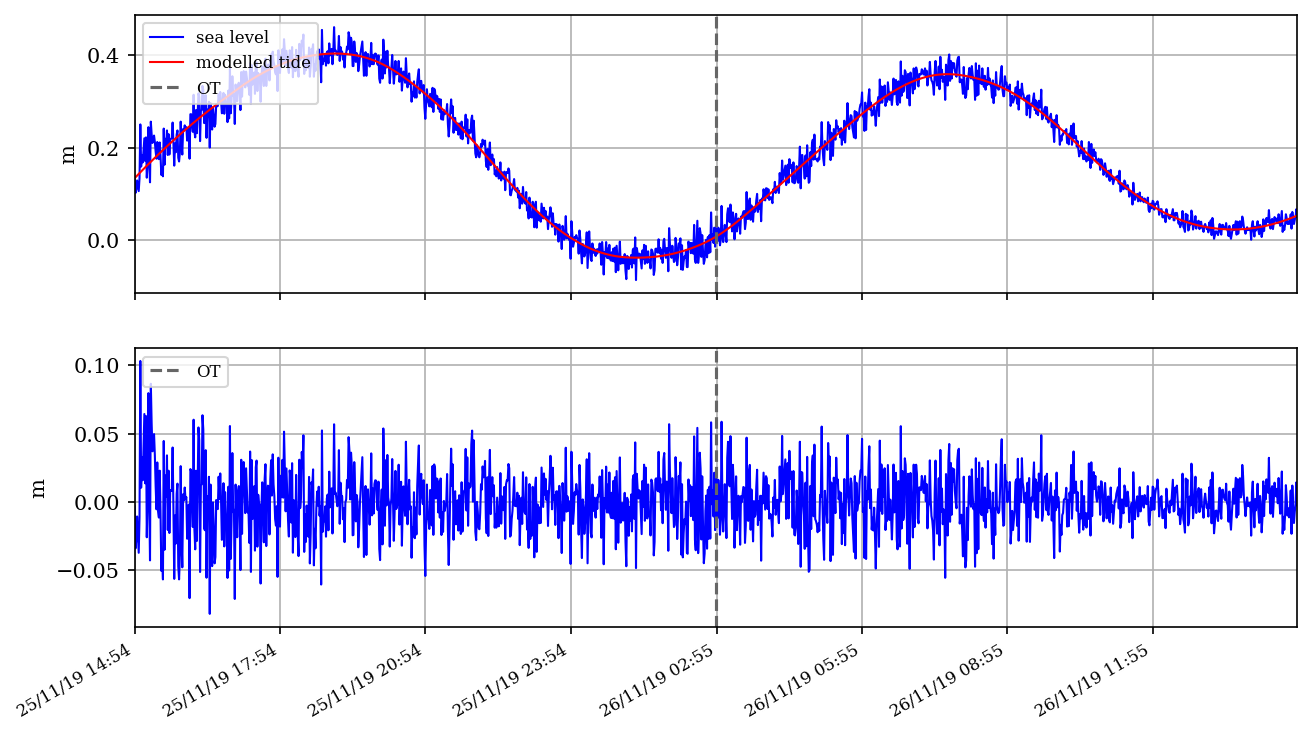

Supplement: Supplementary file 6 — Supplementary Dataset 3. Sea-level records. [file 41467_2021_25815_MOESM6_ESM.zip › sea_level_records/2019-11-26_Albania/PL14.rad.rmn.png]

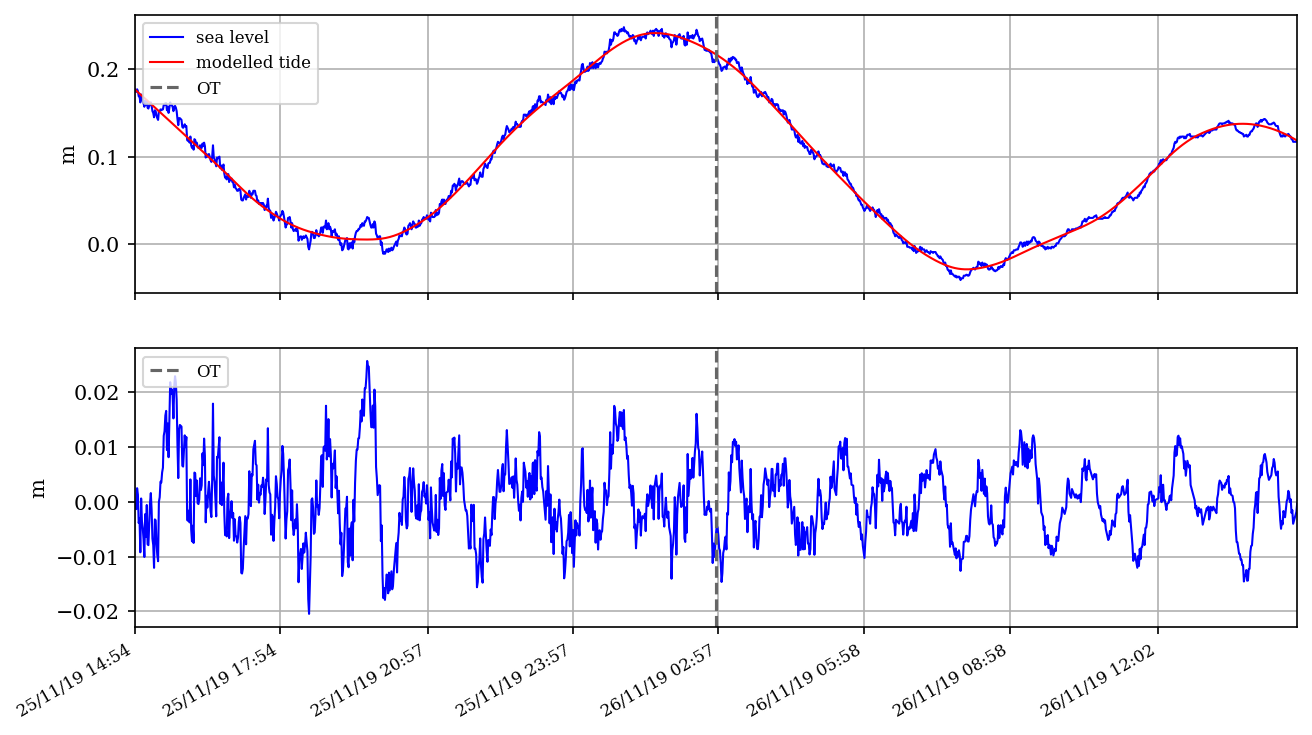

Supplement: Supplementary file 6 — Supplementary Dataset 3. Sea-level records. [file 41467_2021_25815_MOESM6_ESM.zip › sea_level_records/2019-11-26_Albania/TA18.rad.rmn.png]

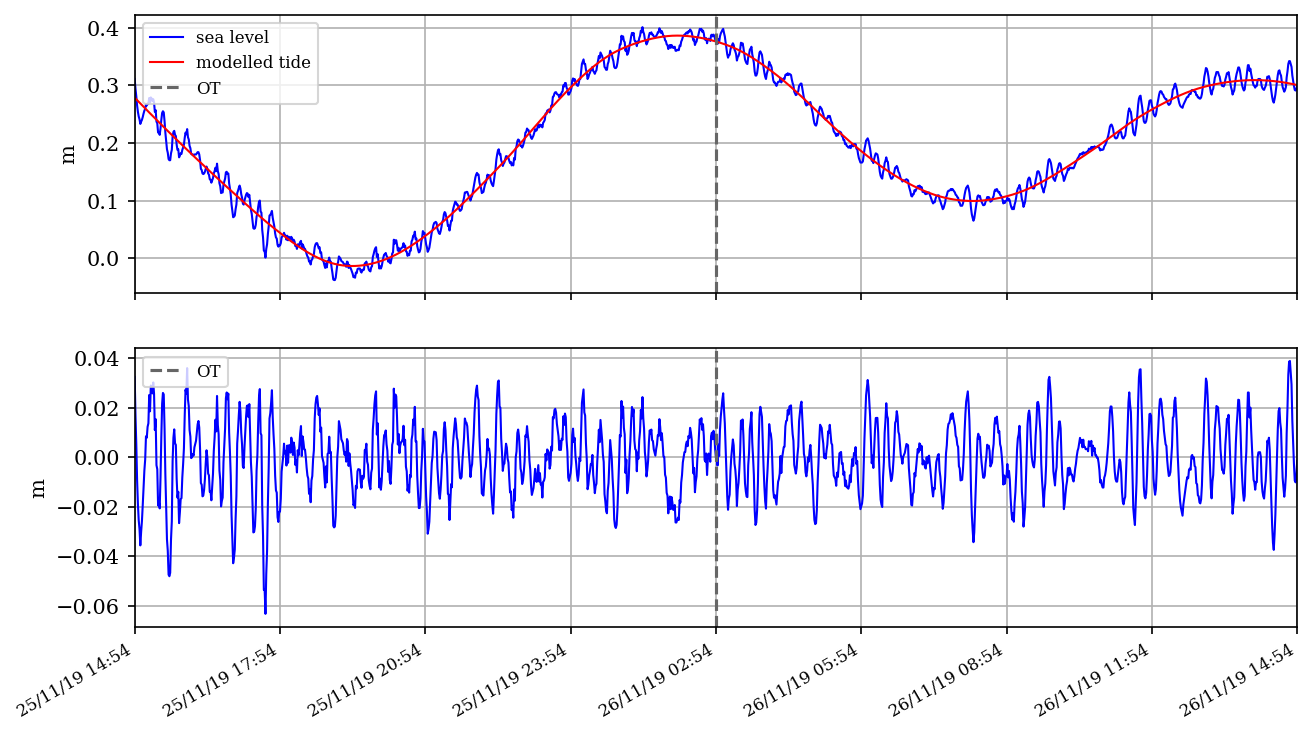

Supplement: Supplementary file 6 — Supplementary Dataset 3. Sea-level records. [file 41467_2021_25815_MOESM6_ESM.zip › sea_level_records/2019-11-26_Albania/VI12.rad.rmn.png]

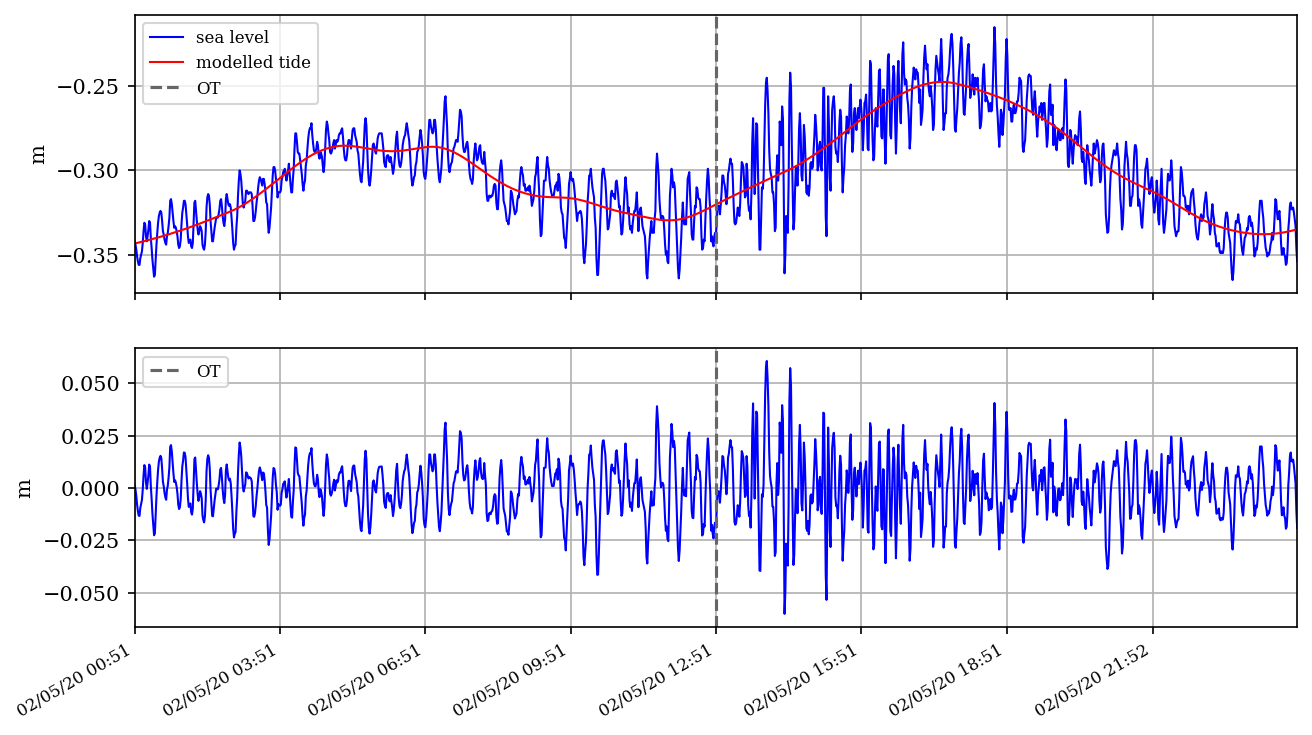

Supplement: Supplementary file 6 — Supplementary Dataset 3. Sea-level records. [file 41467_2021_25815_MOESM6_ESM.zip › sea_level_records/2020-05-02_Creta/NOA03.rad.rmn.png]

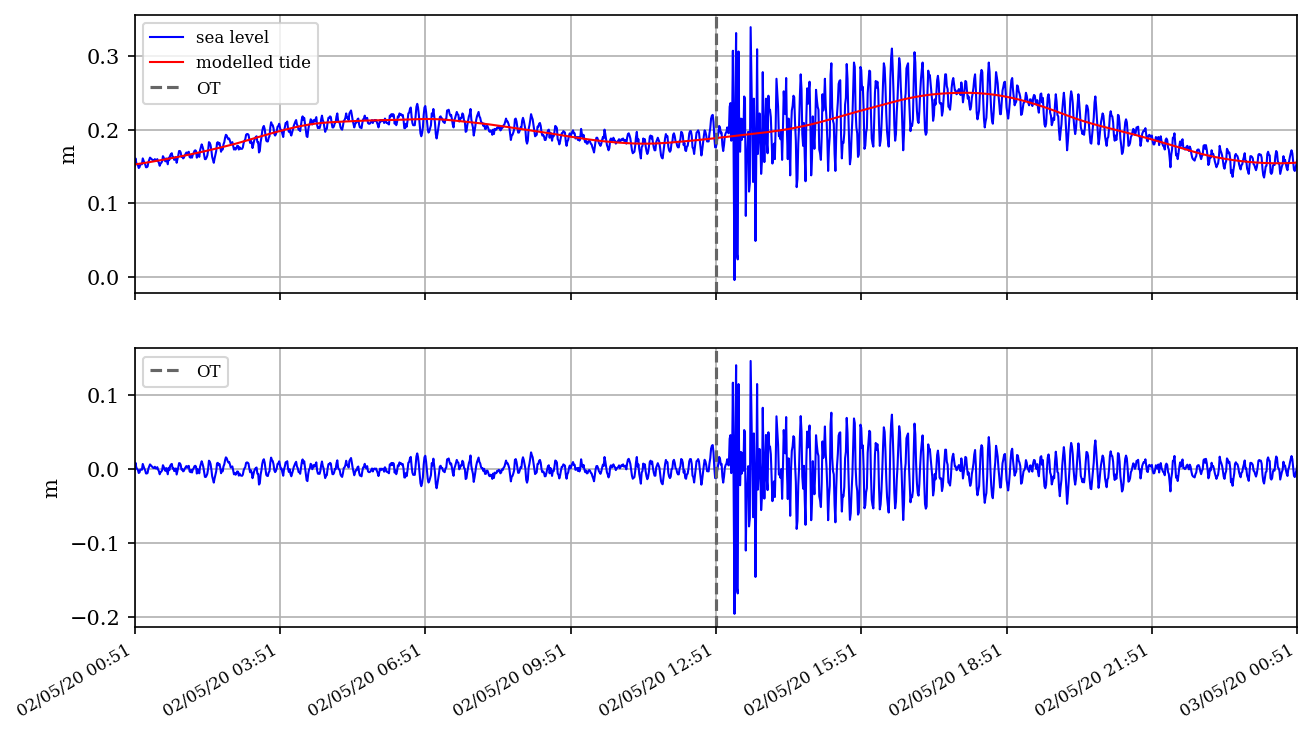

Supplement: Supplementary file 6 — Supplementary Dataset 3. Sea-level records. [file 41467_2021_25815_MOESM6_ESM.zip › sea_level_records/2020-05-02_Creta/NOA04.rad.rmn.png]

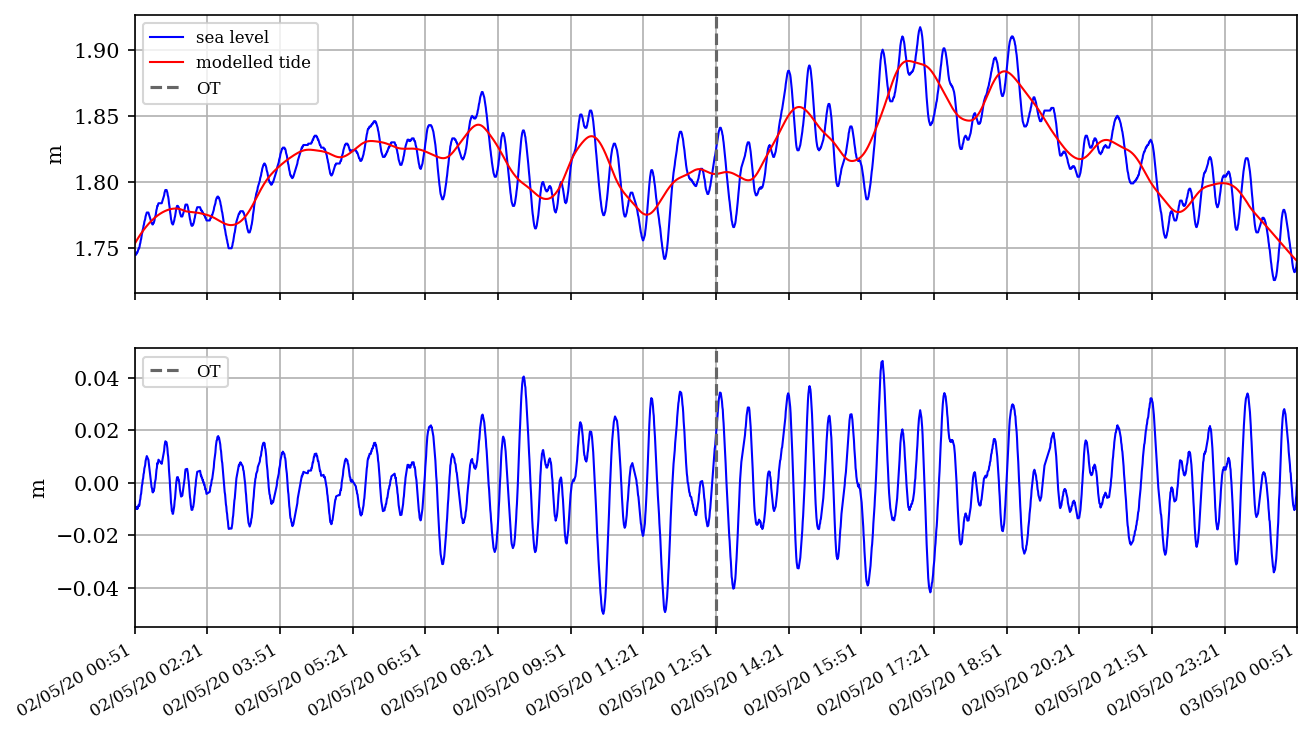

Supplement: Supplementary file 6 — Supplementary Dataset 3. Sea-level records. [file 41467_2021_25815_MOESM6_ESM.zip › sea_level_records/2020-05-02_Creta/bodru.rad.rmn.png]

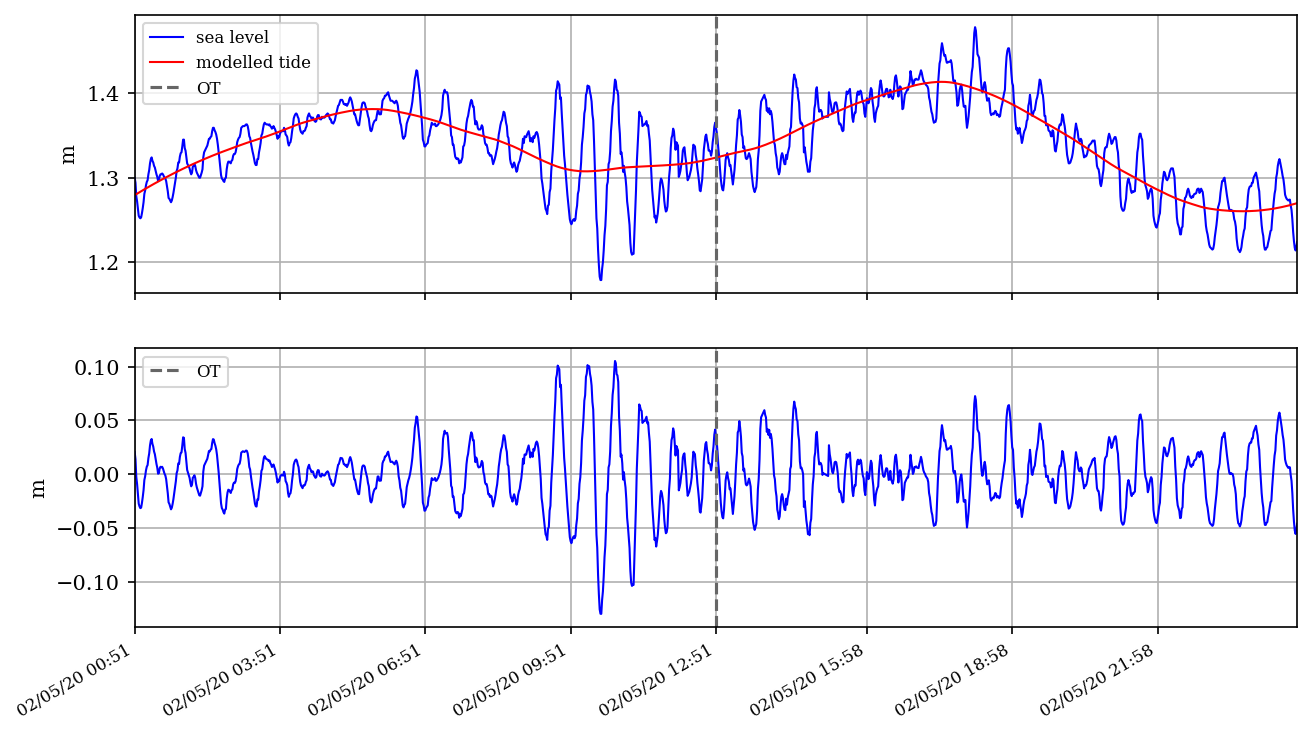

Supplement: Supplementary file 6 — Supplementary Dataset 3. Sea-level records. [file 41467_2021_25815_MOESM6_ESM.zip › sea_level_records/2020-05-02_Creta/feth.rad.rmn.png]

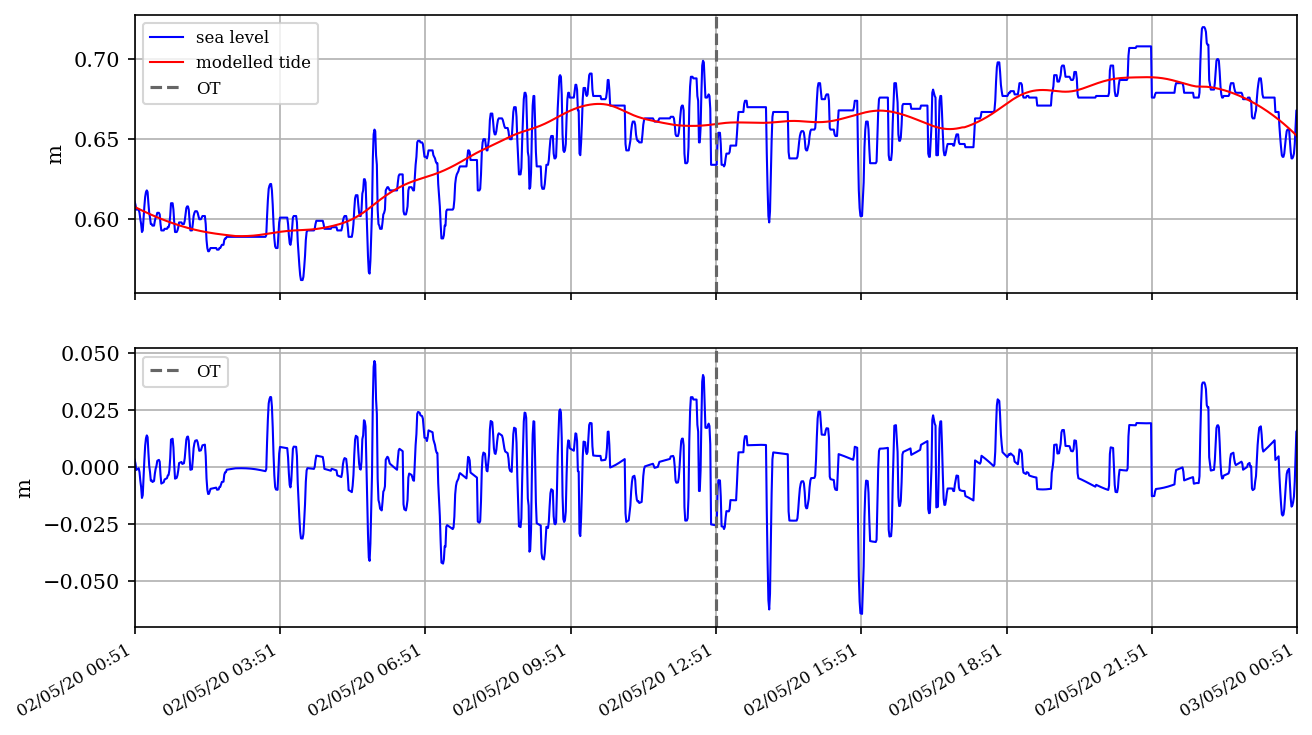

Supplement: Supplementary file 6 — Supplementary Dataset 3. Sea-level records. [file 41467_2021_25815_MOESM6_ESM.zip › sea_level_records/2020-05-02_Creta/syro.pr1.rmn.png]

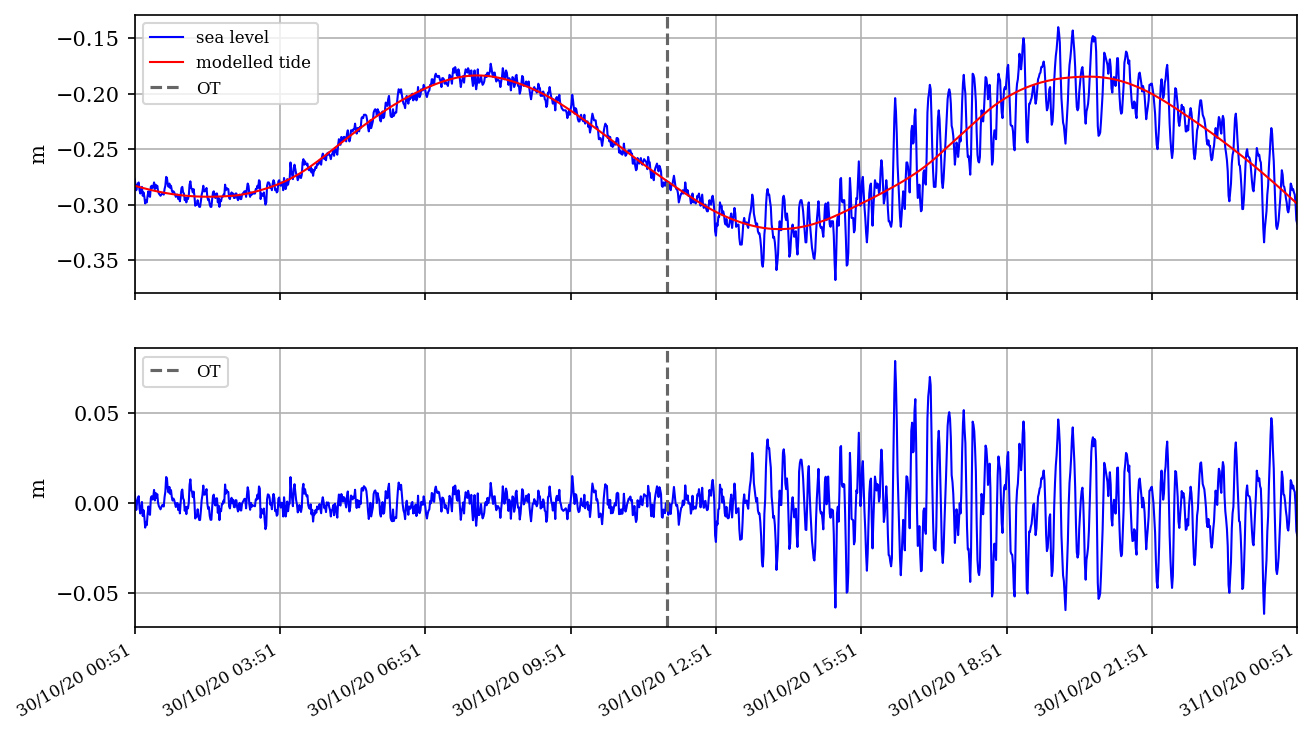

Supplement: Supplementary file 6 — Supplementary Dataset 3. Sea-level records. [file 41467_2021_25815_MOESM6_ESM.zip › sea_level_records/2020-10-30_Samos/NOA03.rad.rmn.png]

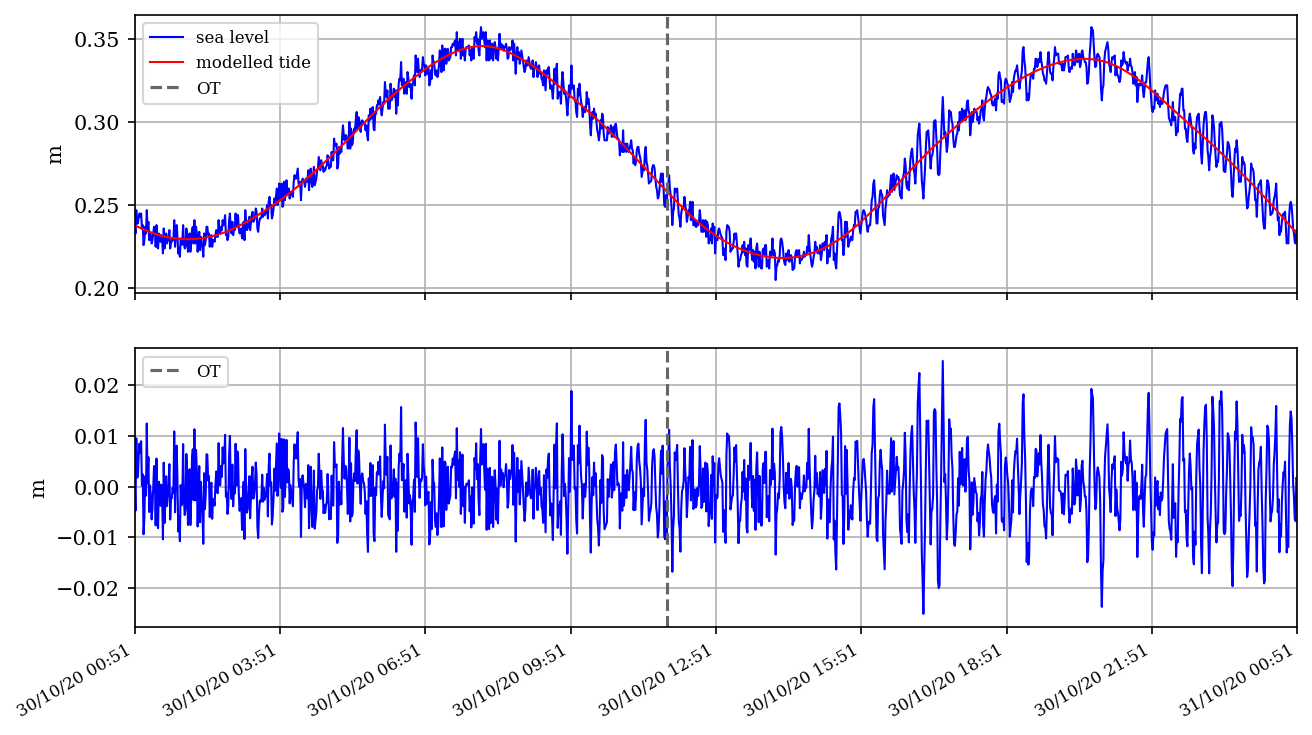

Supplement: Supplementary file 6 — Supplementary Dataset 3. Sea-level records. [file 41467_2021_25815_MOESM6_ESM.zip › sea_level_records/2020-10-30_Samos/NOA04.rad.rmn.png]

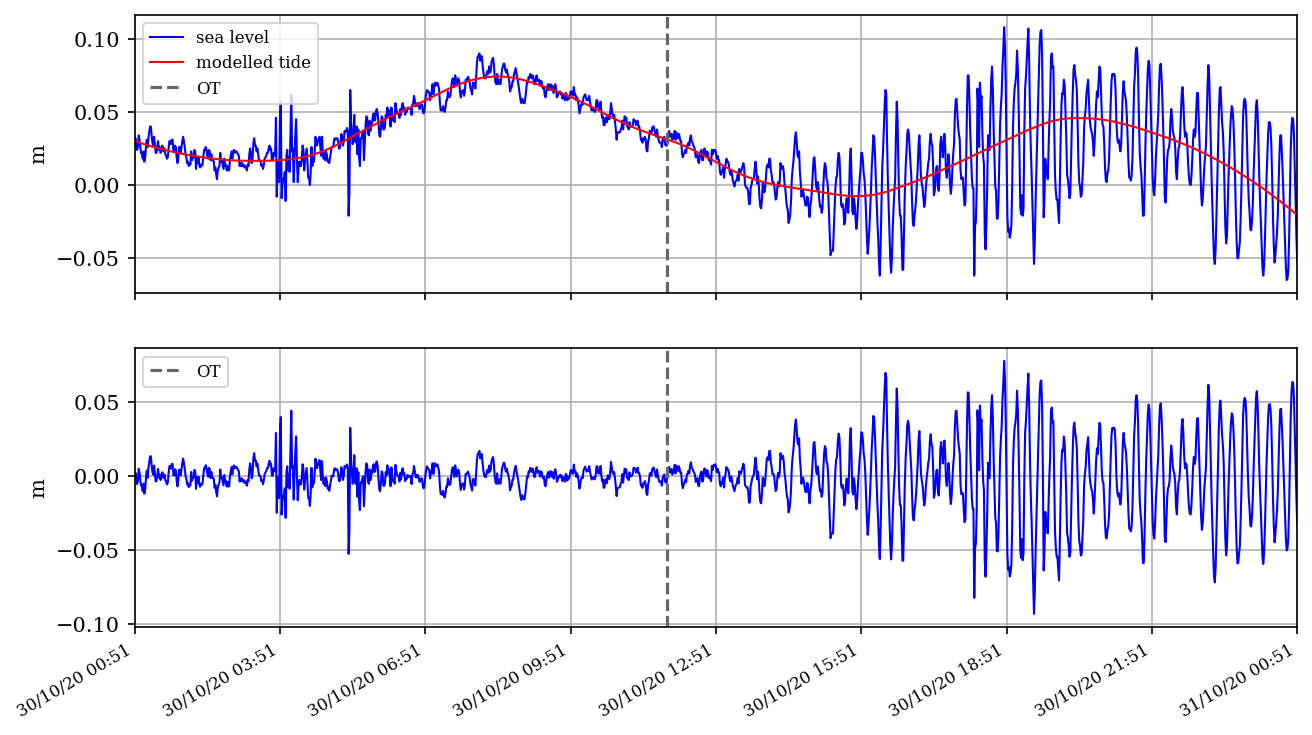

Supplement: Supplementary file 6 — Supplementary Dataset 3. Sea-level records. [file 41467_2021_25815_MOESM6_ESM.zip › sea_level_records/2020-10-30_Samos/NOA10.rad.rmn.png]

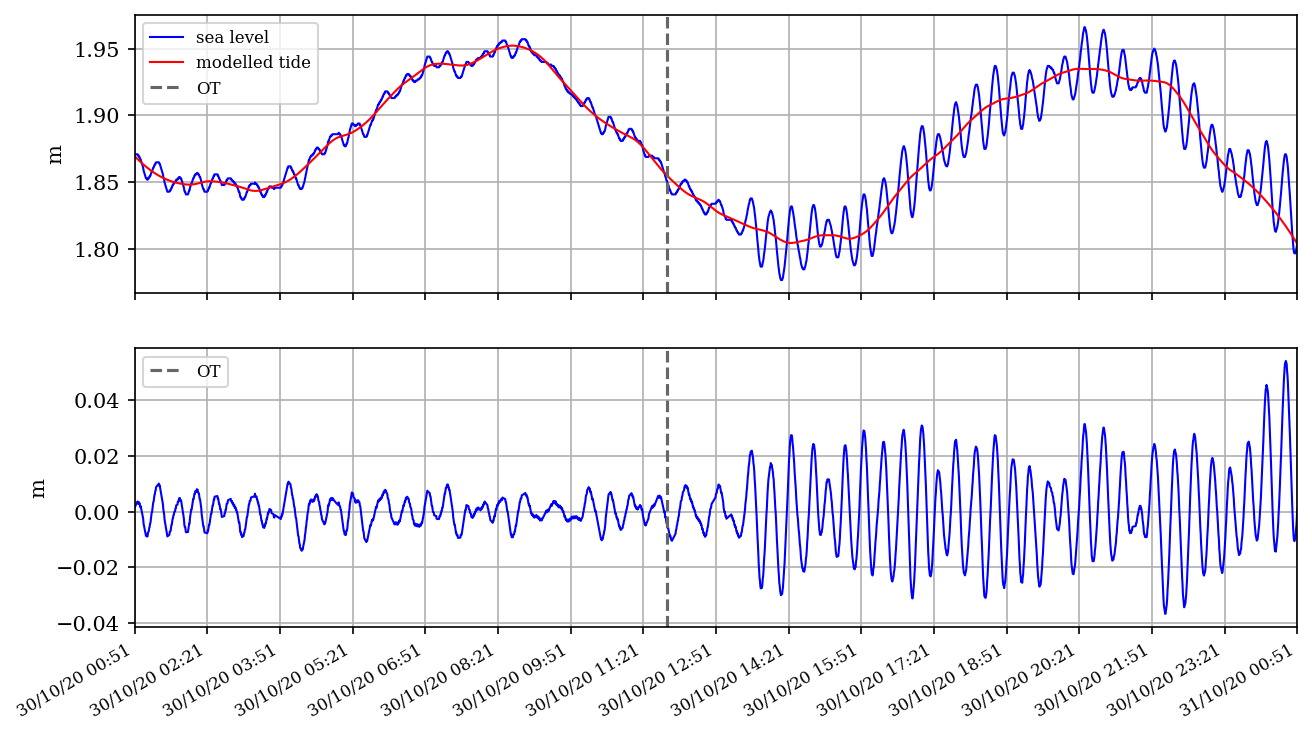

Supplement: Supplementary file 6 — Supplementary Dataset 3. Sea-level records. [file 41467_2021_25815_MOESM6_ESM.zip › sea_level_records/2020-10-30_Samos/bodru.rad.rmn.png]

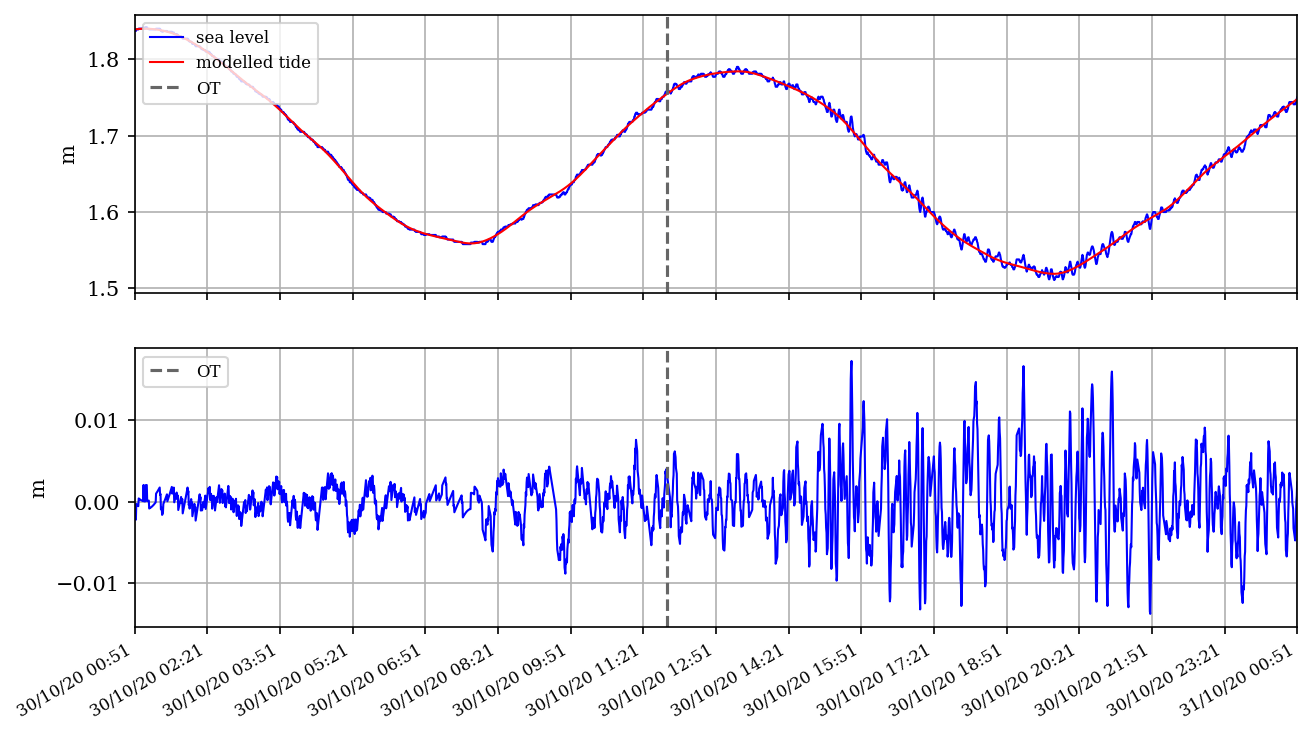

Supplement: Supplementary file 6 — Supplementary Dataset 3. Sea-level records. [file 41467_2021_25815_MOESM6_ESM.zip › sea_level_records/2020-10-30_Samos/gokce.rad.rmn.png]

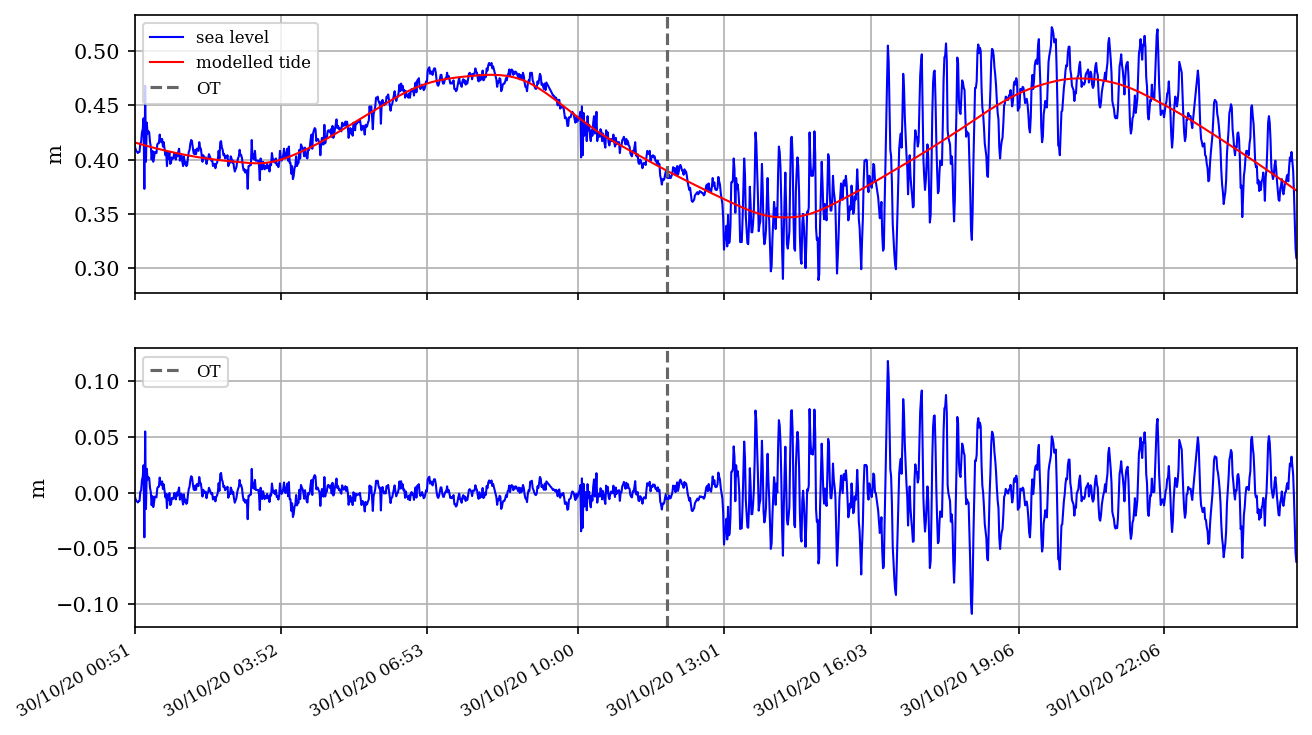

Supplement: Supplementary file 6 — Supplementary Dataset 3. Sea-level records. [file 41467_2021_25815_MOESM6_ESM.zip › sea_level_records/2020-10-30_Samos/kos1.rad.rmn.png]

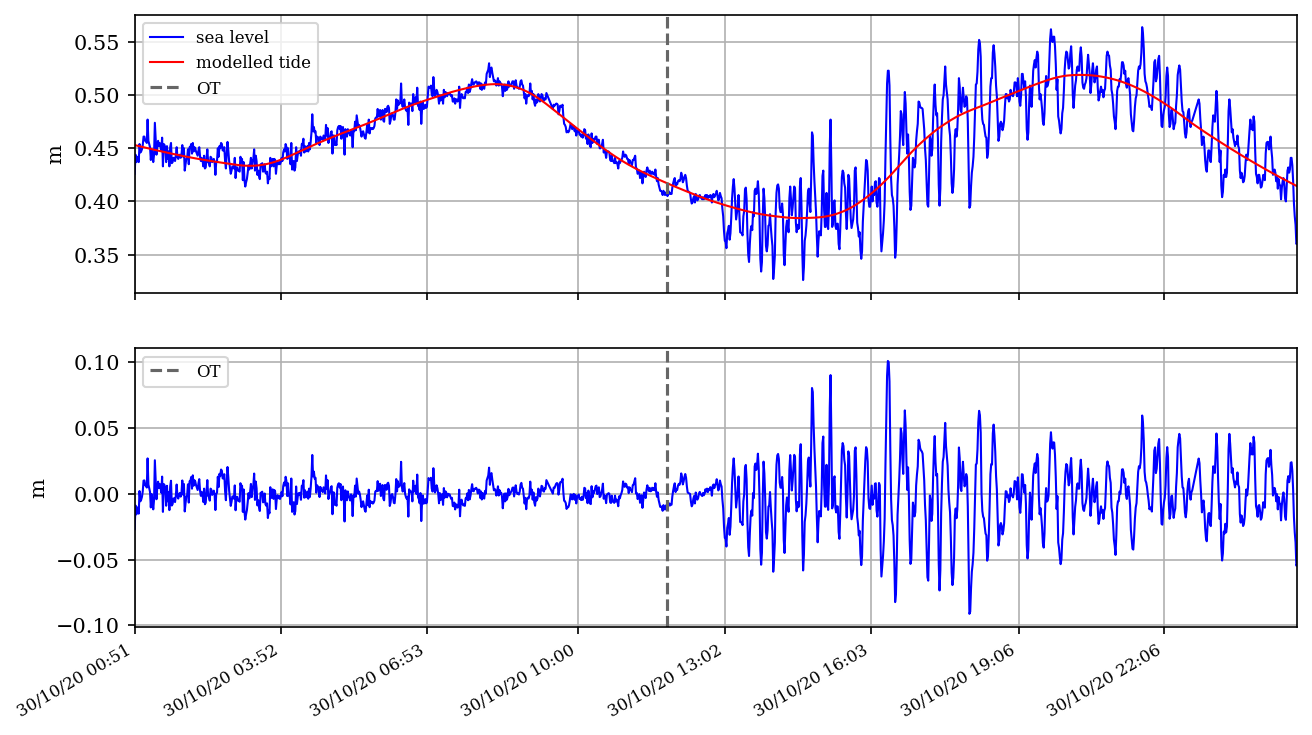

Supplement: Supplementary file 6 — Supplementary Dataset 3. Sea-level records. [file 41467_2021_25815_MOESM6_ESM.zip › sea_level_records/2020-10-30_Samos/kos2.rad.rmn.png]

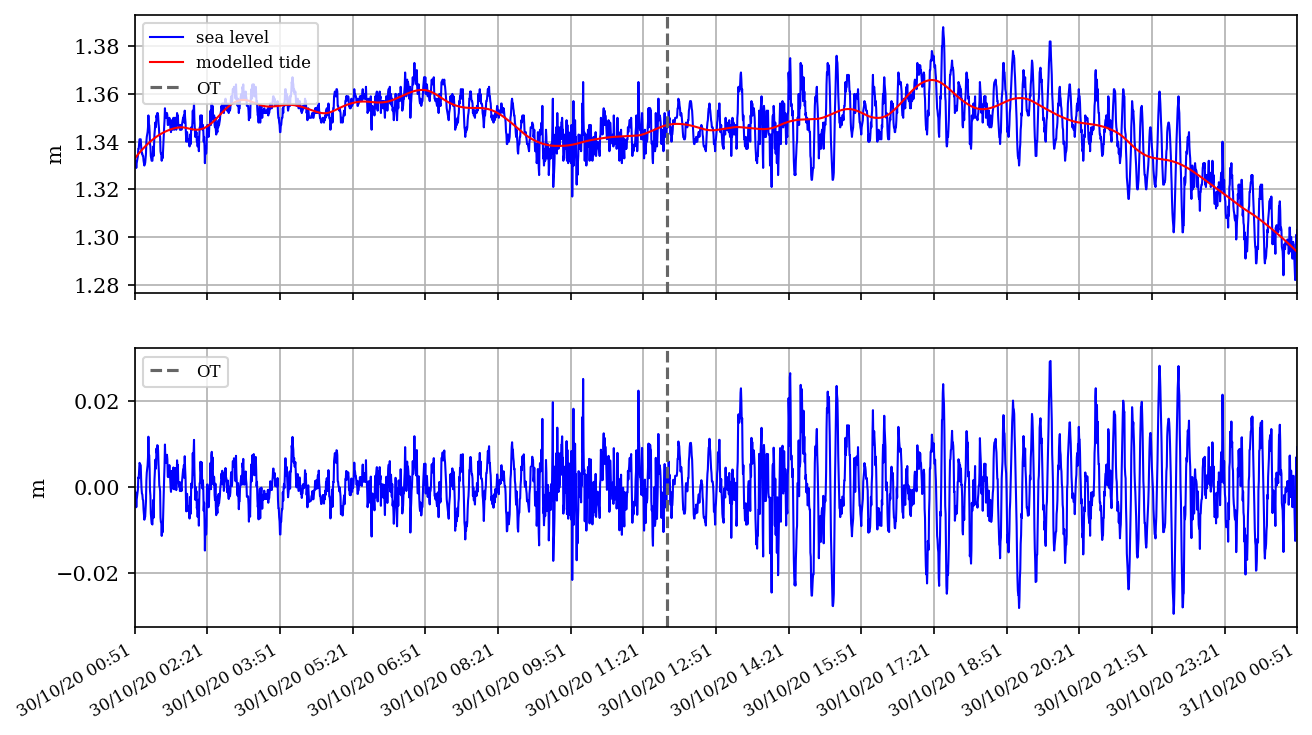

Supplement: Supplementary file 6 — Supplementary Dataset 3. Sea-level records. [file 41467_2021_25815_MOESM6_ESM.zip › sea_level_records/2020-10-30_Samos/marma.rad.rmn.png]

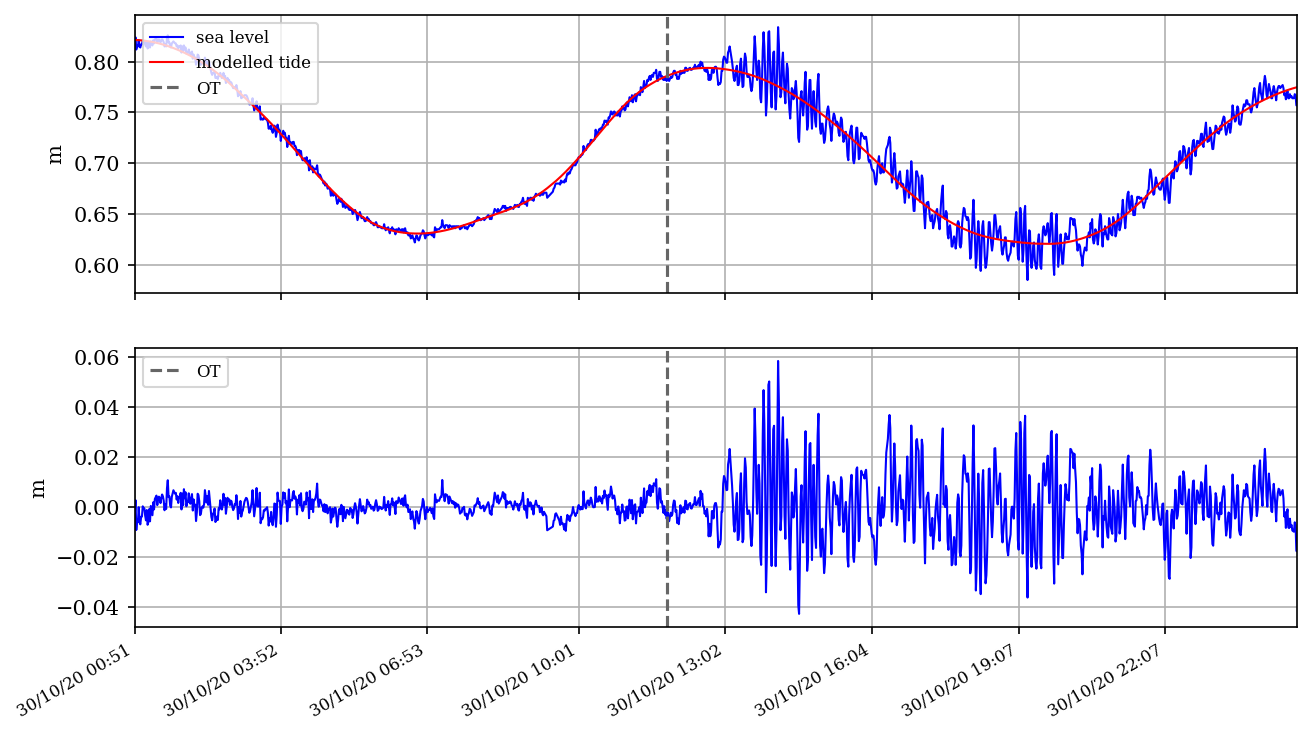

Supplement: Supplementary file 6 — Supplementary Dataset 3. Sea-level records. [file 41467_2021_25815_MOESM6_ESM.zip › sea_level_records/2020-10-30_Samos/plom.rad.rmn.png]

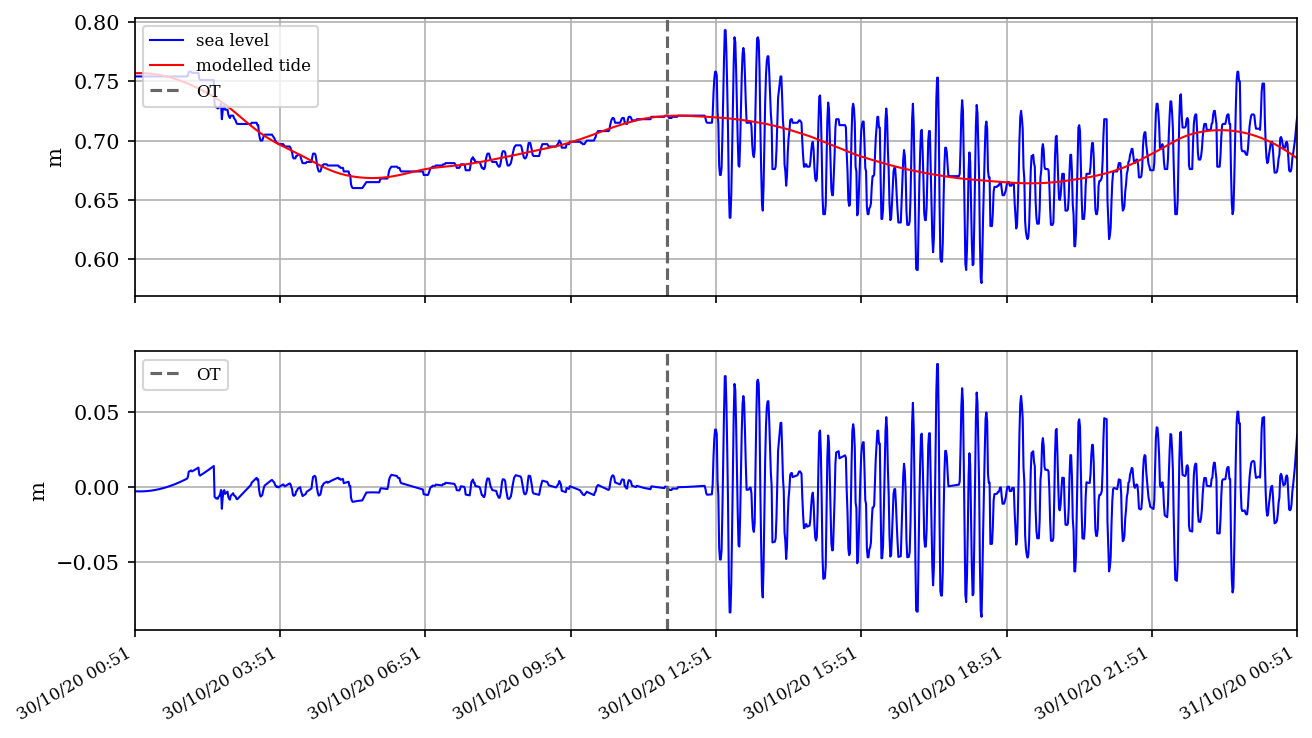

Supplement: Supplementary file 6 — Supplementary Dataset 3. Sea-level records. [file 41467_2021_25815_MOESM6_ESM.zip › sea_level_records/2020-10-30_Samos/syro.pr1.rmn.png]

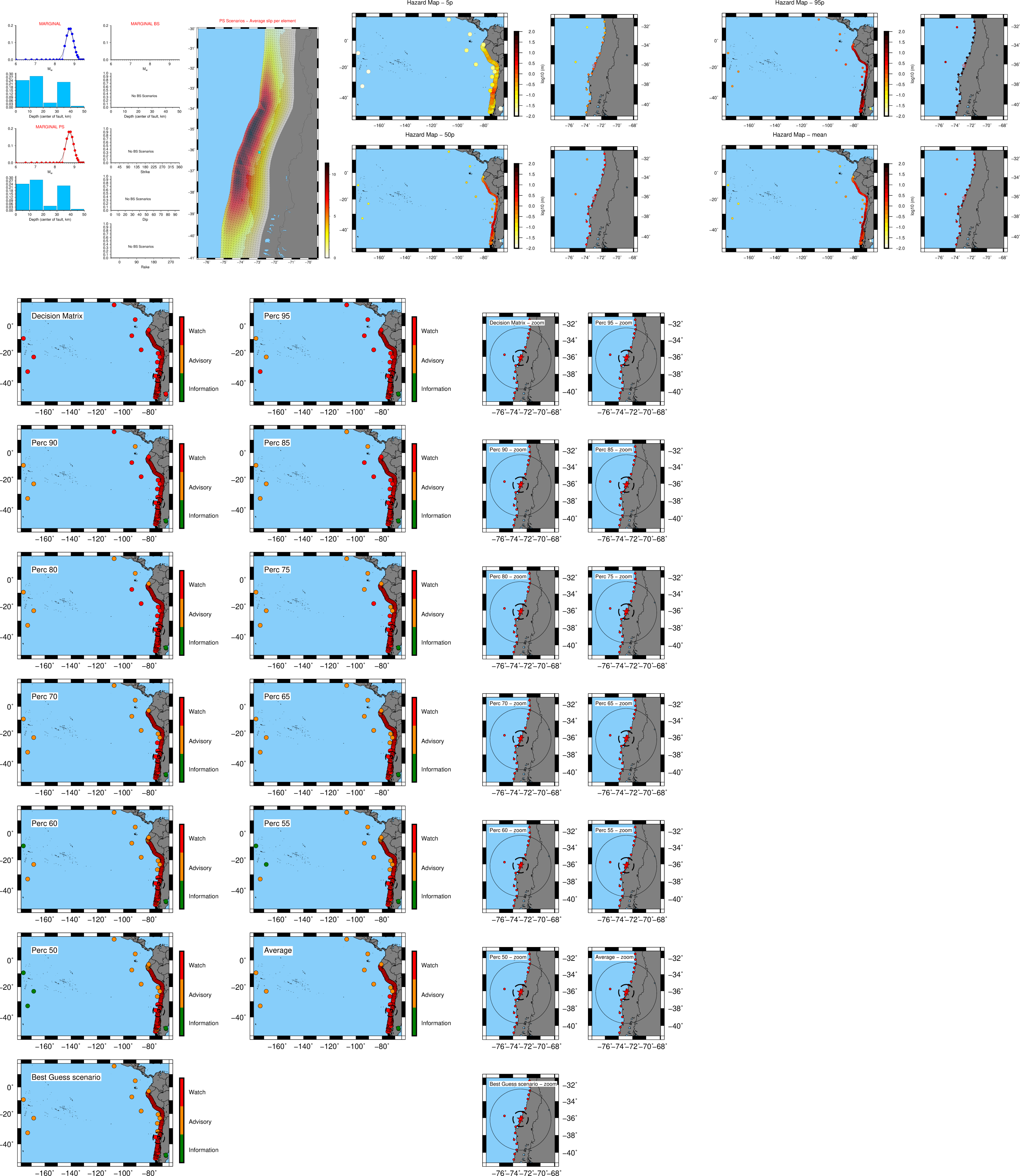

Supplement: Supplementary file 7 — Supplementary Dataset 4. Automatic figures produced by PTF. [file 41467_2021_25815_MOESM7_ESM.zip › ALL_fig_2010_0227_maule.png]

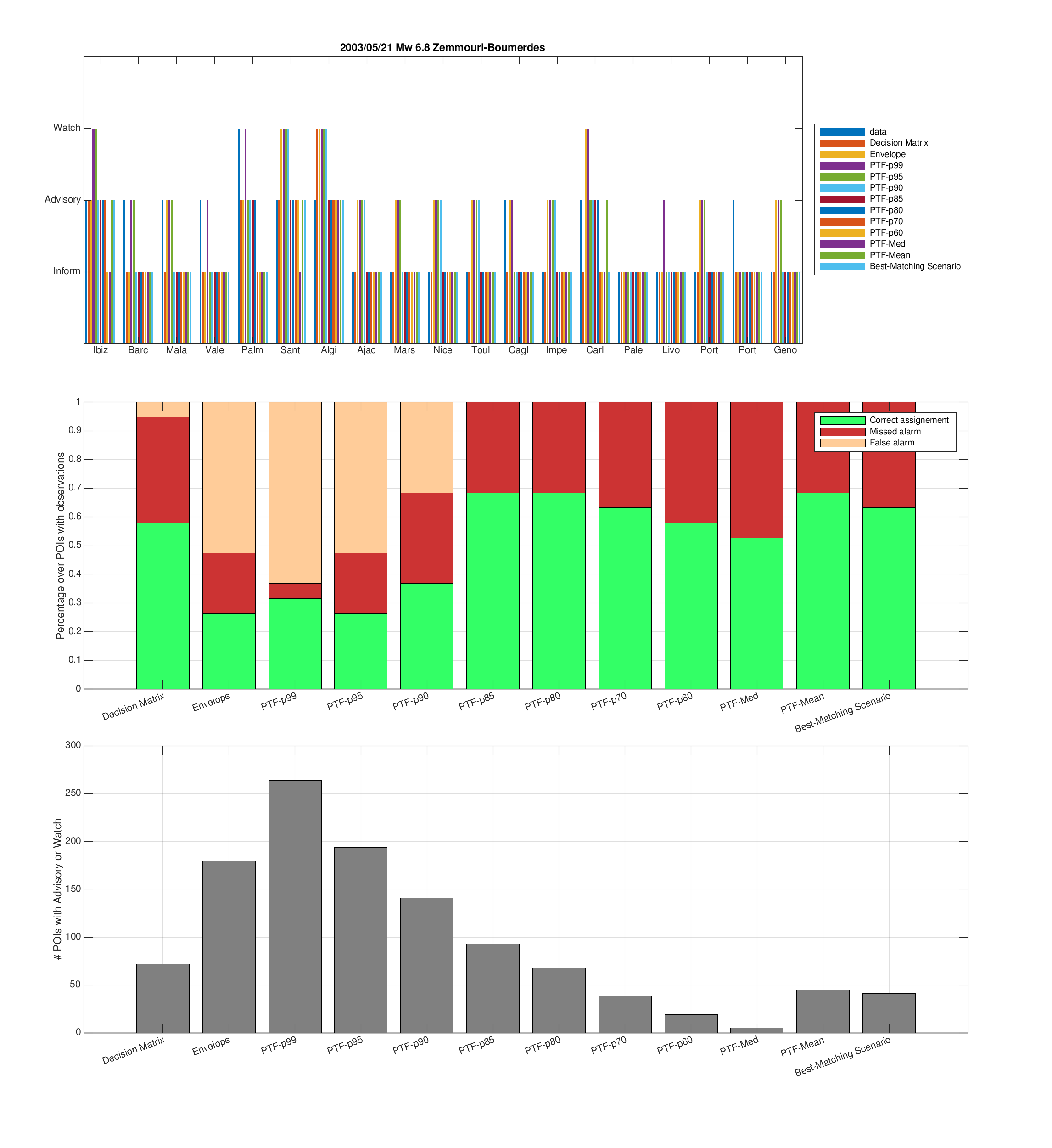

Supplement: Supplementary file 8 — Supplementary Dataset 5. Alert levels comparison figures. [file 41467_2021_25815_MOESM8_ESM.zip › Test4_2003_0521_boumardes_sig20_ALs.png]

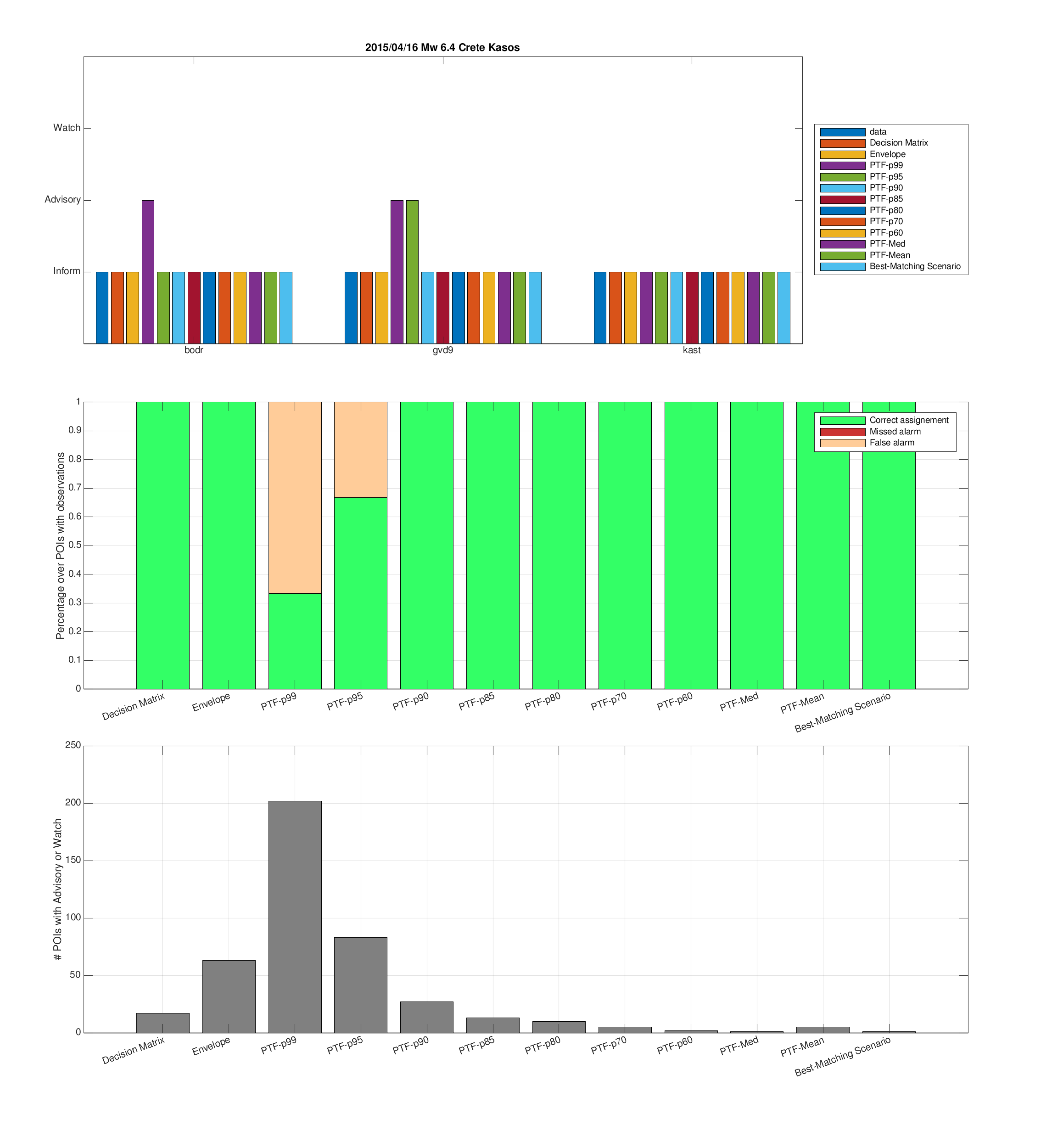

Supplement: Supplementary file 8 — Supplementary Dataset 5. Alert levels comparison figures. [file 41467_2021_25815_MOESM8_ESM.zip › Test4_2015_0416_crete_sig20_ALs.png]

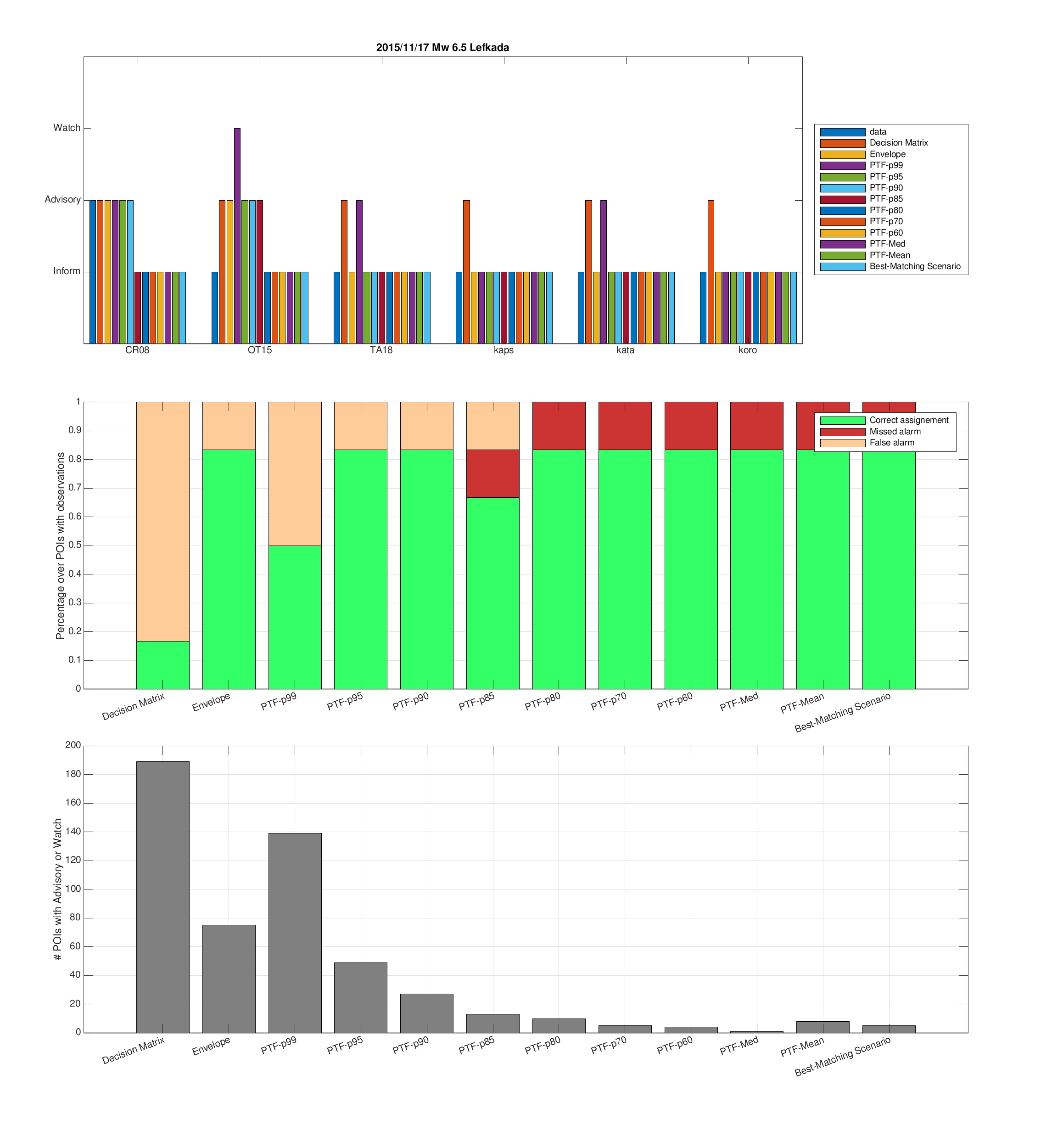

Supplement: Supplementary file 8 — Supplementary Dataset 5. Alert levels comparison figures. [file 41467_2021_25815_MOESM8_ESM.zip › Test4_2015_1117_lefkada_sig20_ALs.png]

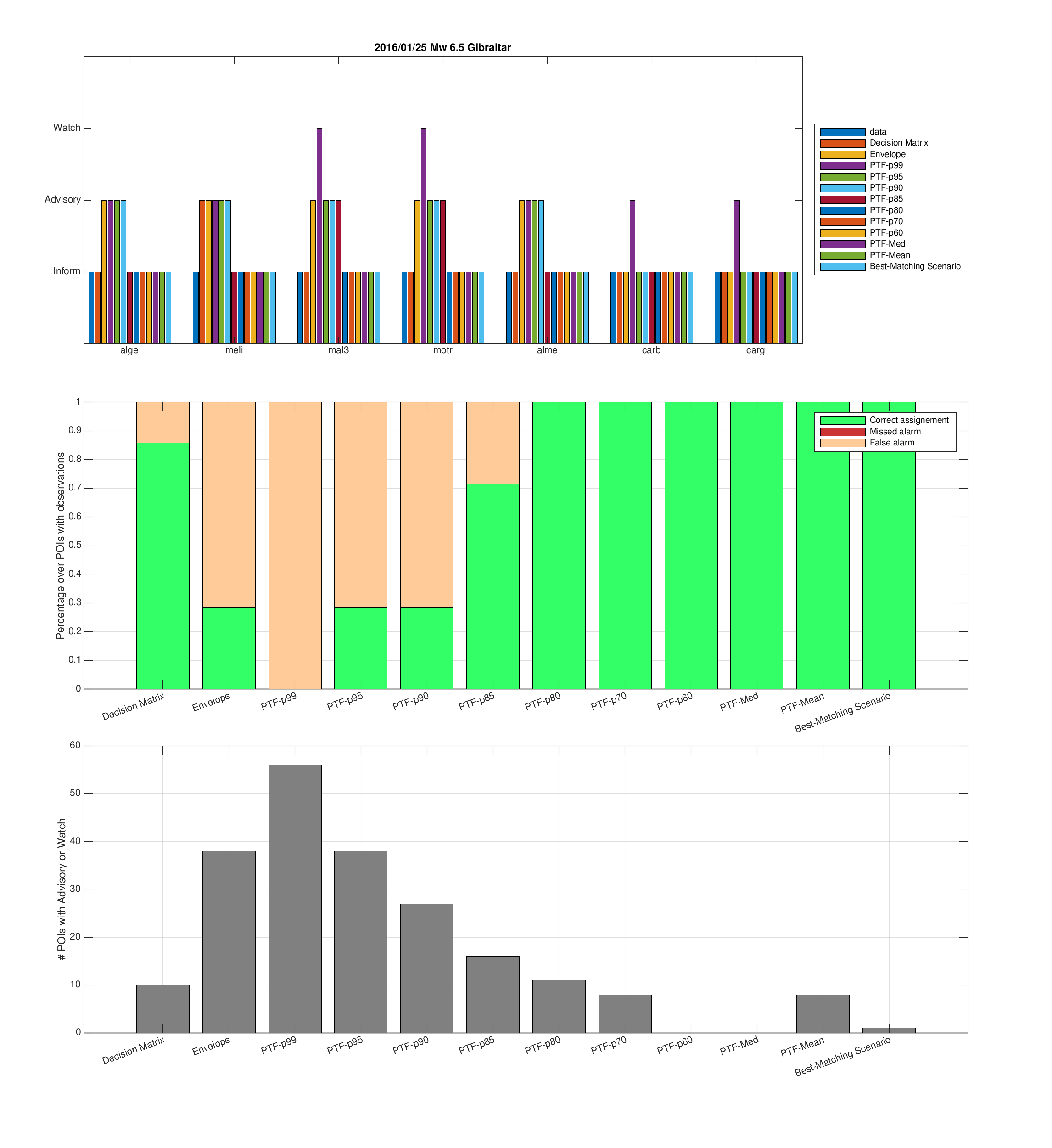

Supplement: Supplementary file 8 — Supplementary Dataset 5. Alert levels comparison figures. [file 41467_2021_25815_MOESM8_ESM.zip › Test4_2016_0125_gibraltar_sig20_ALs.png]

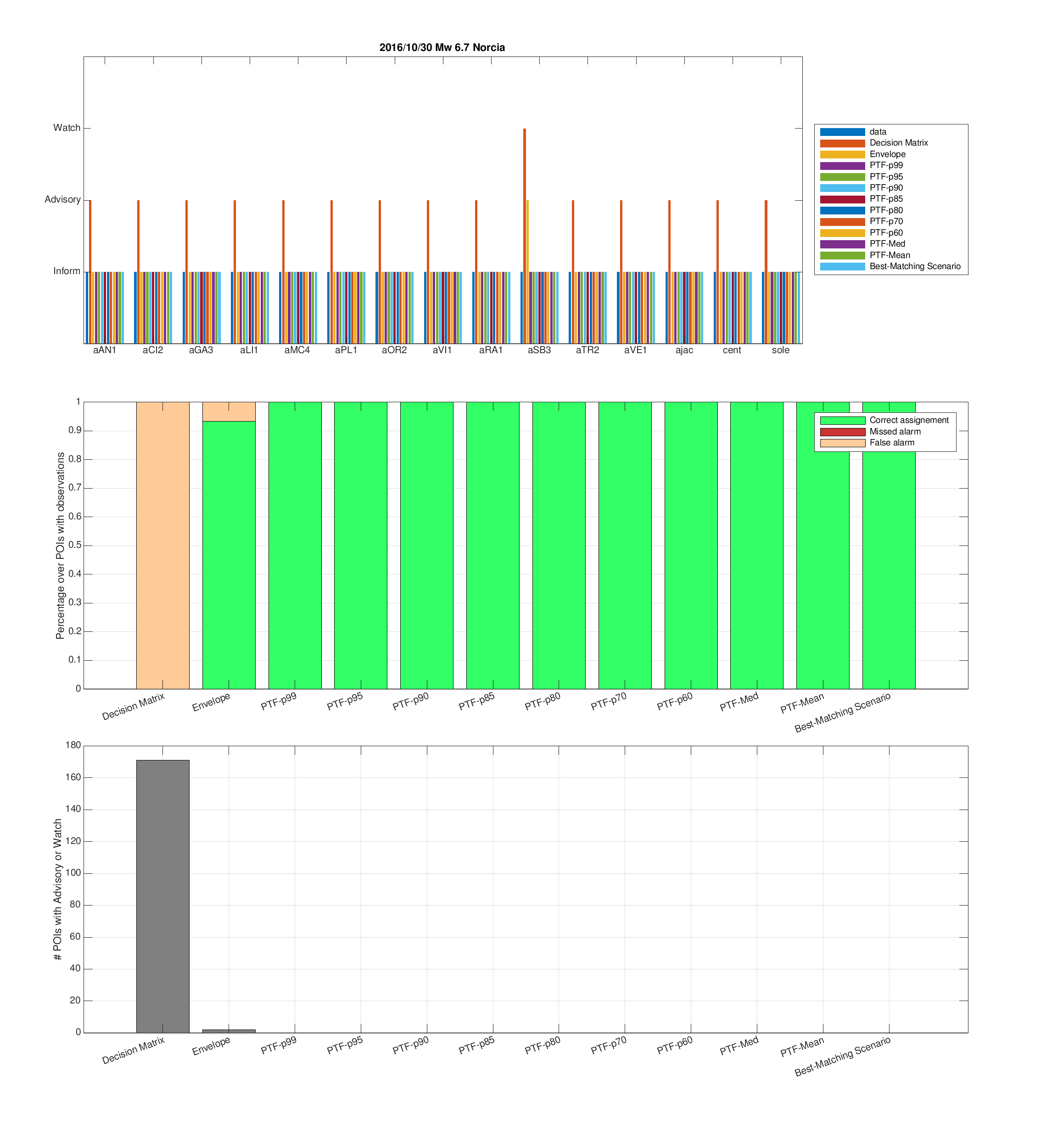

Supplement: Supplementary file 8 — Supplementary Dataset 5. Alert levels comparison figures. [file 41467_2021_25815_MOESM8_ESM.zip › Test4_2016_1030_norcia_sig20_ALs.png]

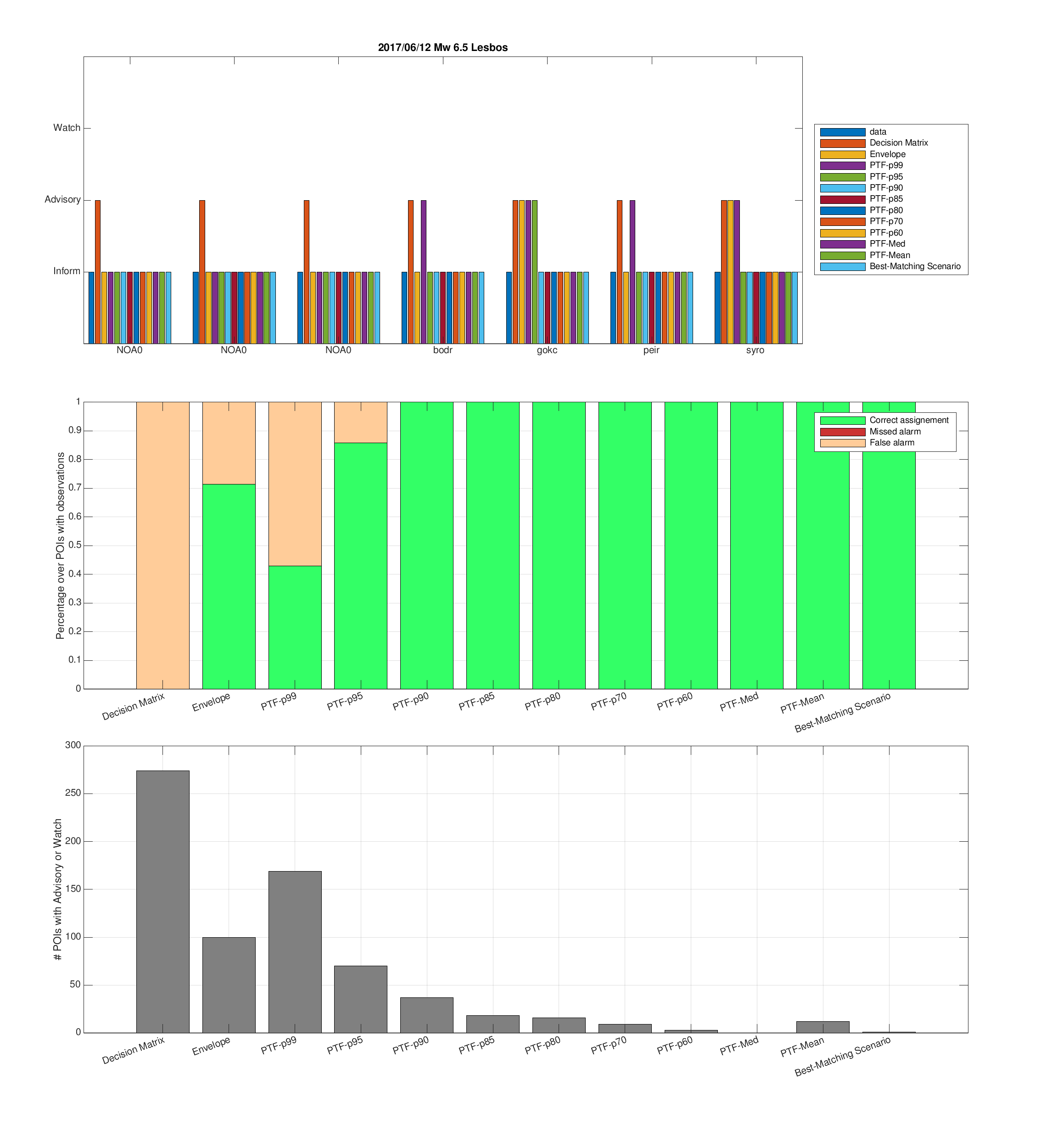

Supplement: Supplementary file 8 — Supplementary Dataset 5. Alert levels comparison figures. [file 41467_2021_25815_MOESM8_ESM.zip › Test4_2017_0612_lesbo_sig20_ALs.png]

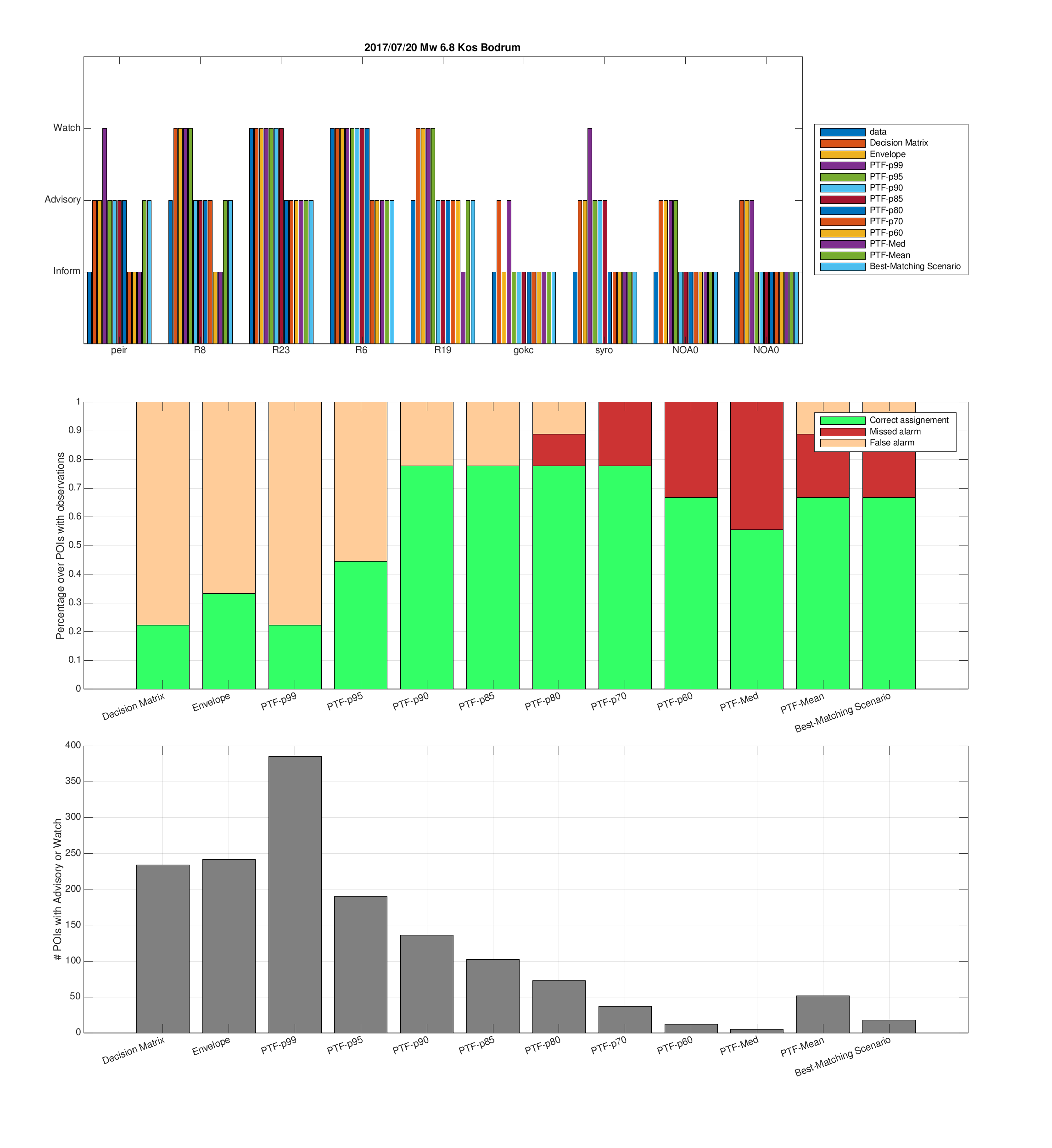

Supplement: Supplementary file 8 — Supplementary Dataset 5. Alert levels comparison figures. [file 41467_2021_25815_MOESM8_ESM.zip › Test4_2017_0720_kos-bodrum_sig20_ALs.png]

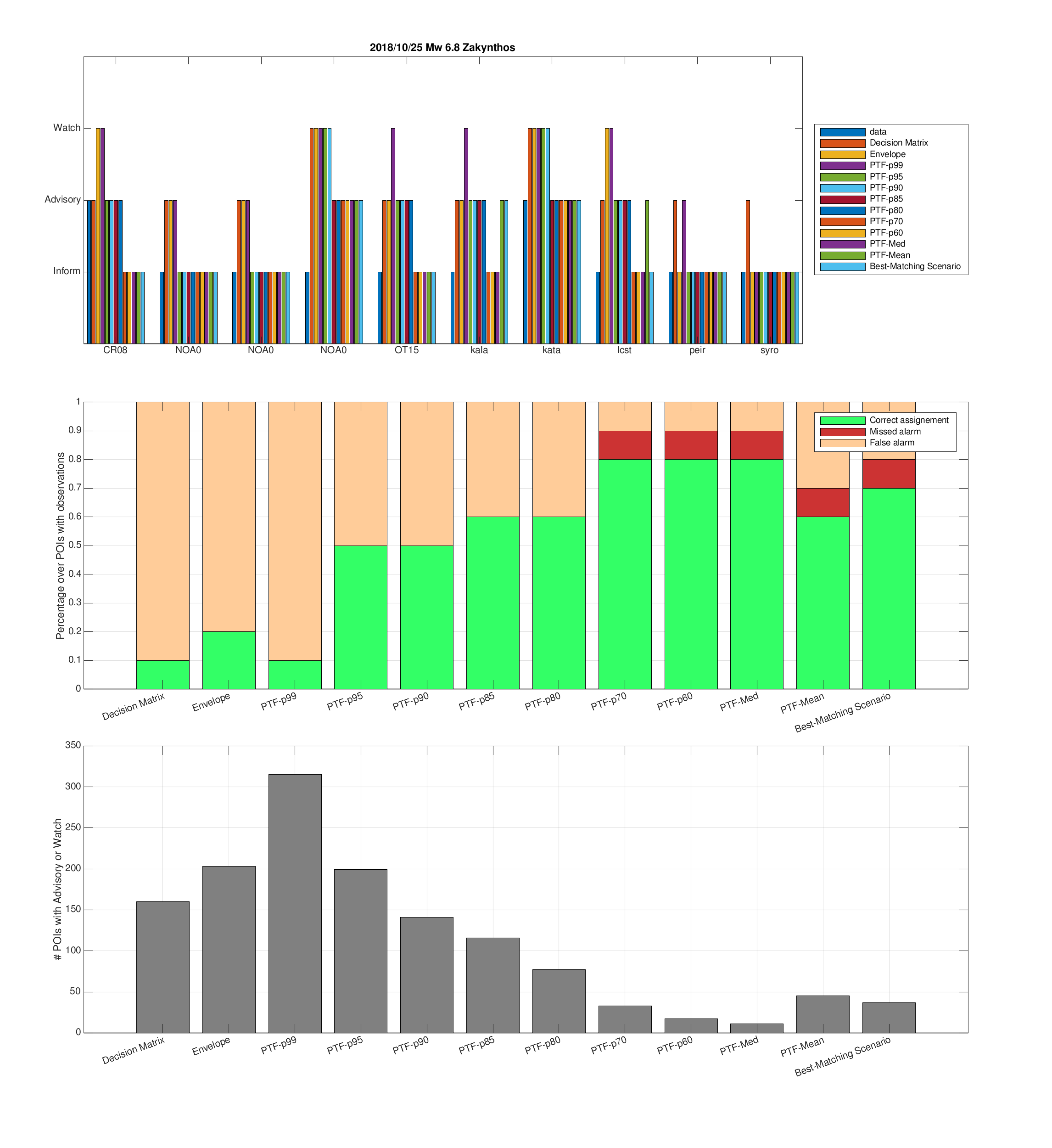

Supplement: Supplementary file 8 — Supplementary Dataset 5. Alert levels comparison figures. [file 41467_2021_25815_MOESM8_ESM.zip › Test4_2018_1025_zante_sig20_ALs.png]

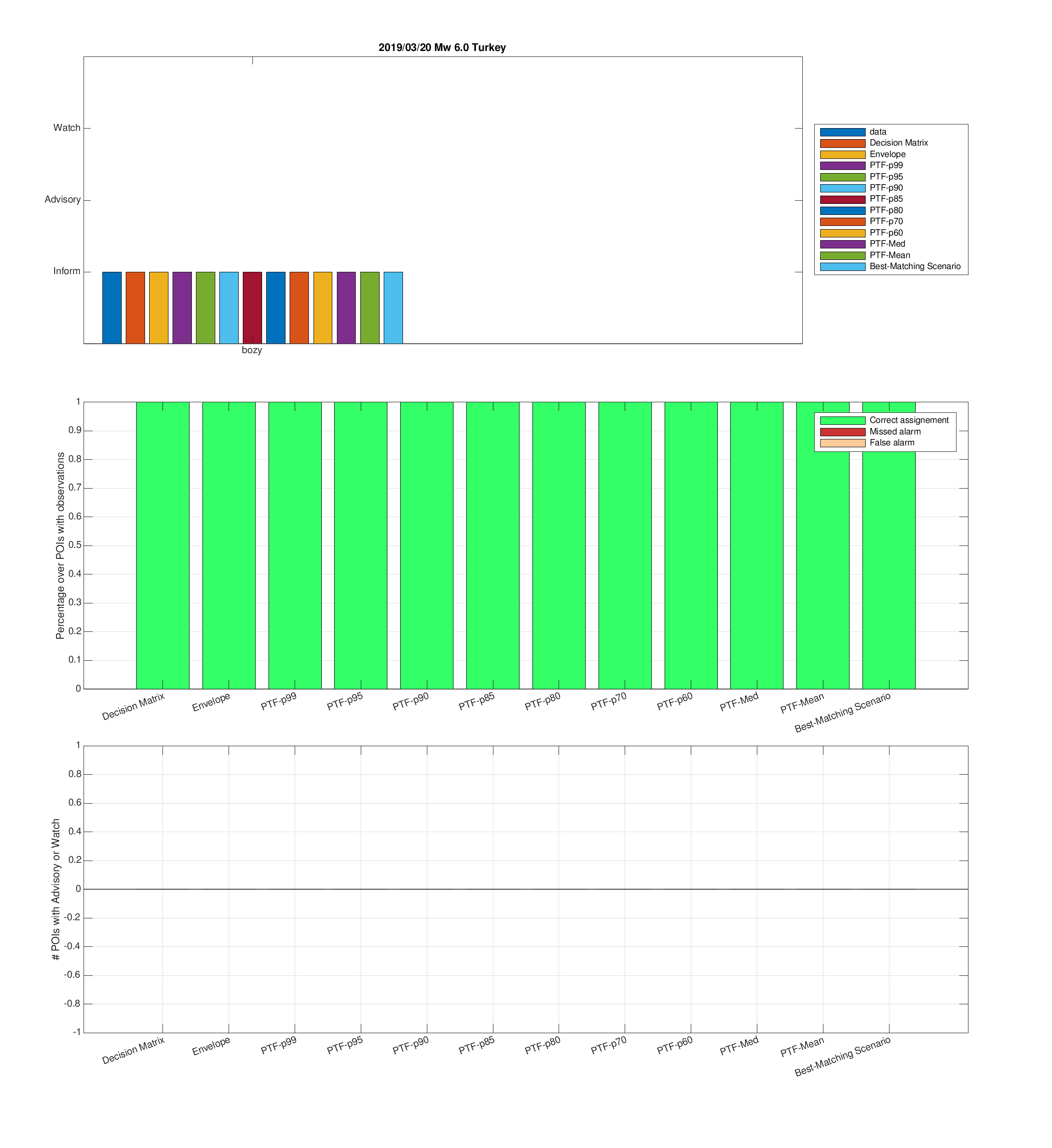

Supplement: Supplementary file 8 — Supplementary Dataset 5. Alert levels comparison figures. [file 41467_2021_25815_MOESM8_ESM.zip › Test4_2019_0320_turkey_sig20_ALs.png]

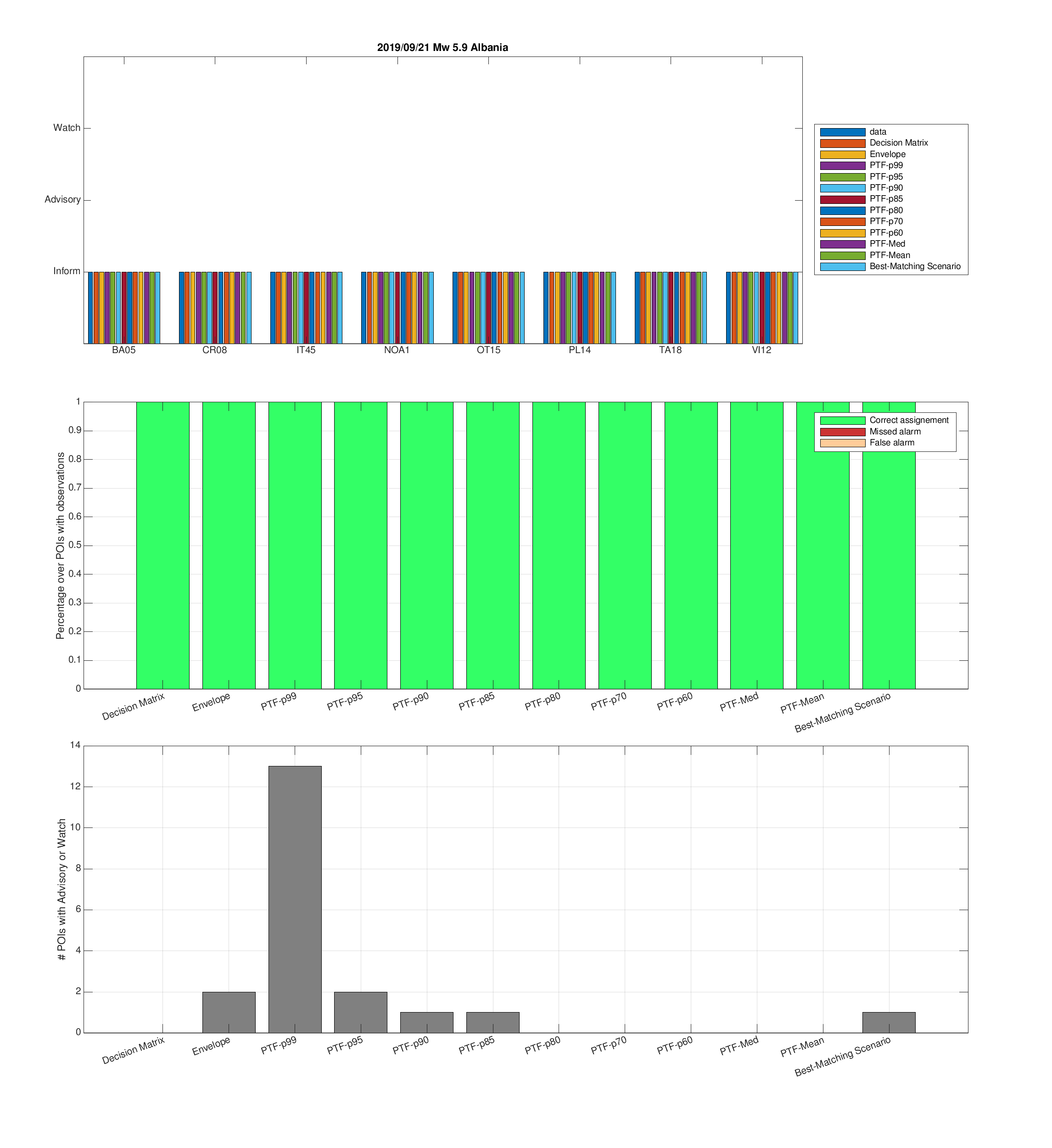

Supplement: Supplementary file 8 — Supplementary Dataset 5. Alert levels comparison figures. [file 41467_2021_25815_MOESM8_ESM.zip › Test4_2019_0921_albania_sig20_ALs.png]

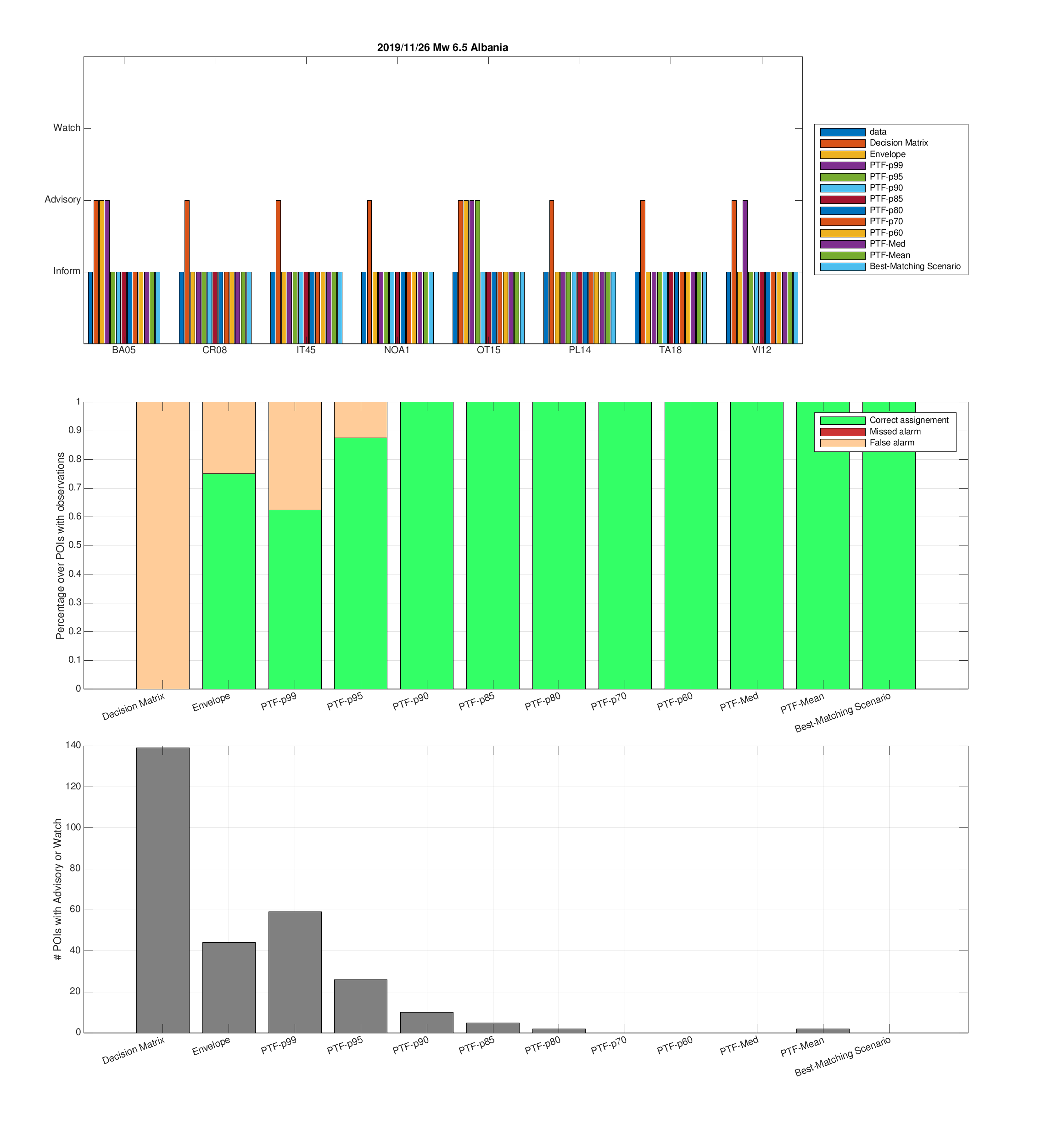

Supplement: Supplementary file 8 — Supplementary Dataset 5. Alert levels comparison figures. [file 41467_2021_25815_MOESM8_ESM.zip › Test4_2019_1126_albania_sig20_ALs.png]

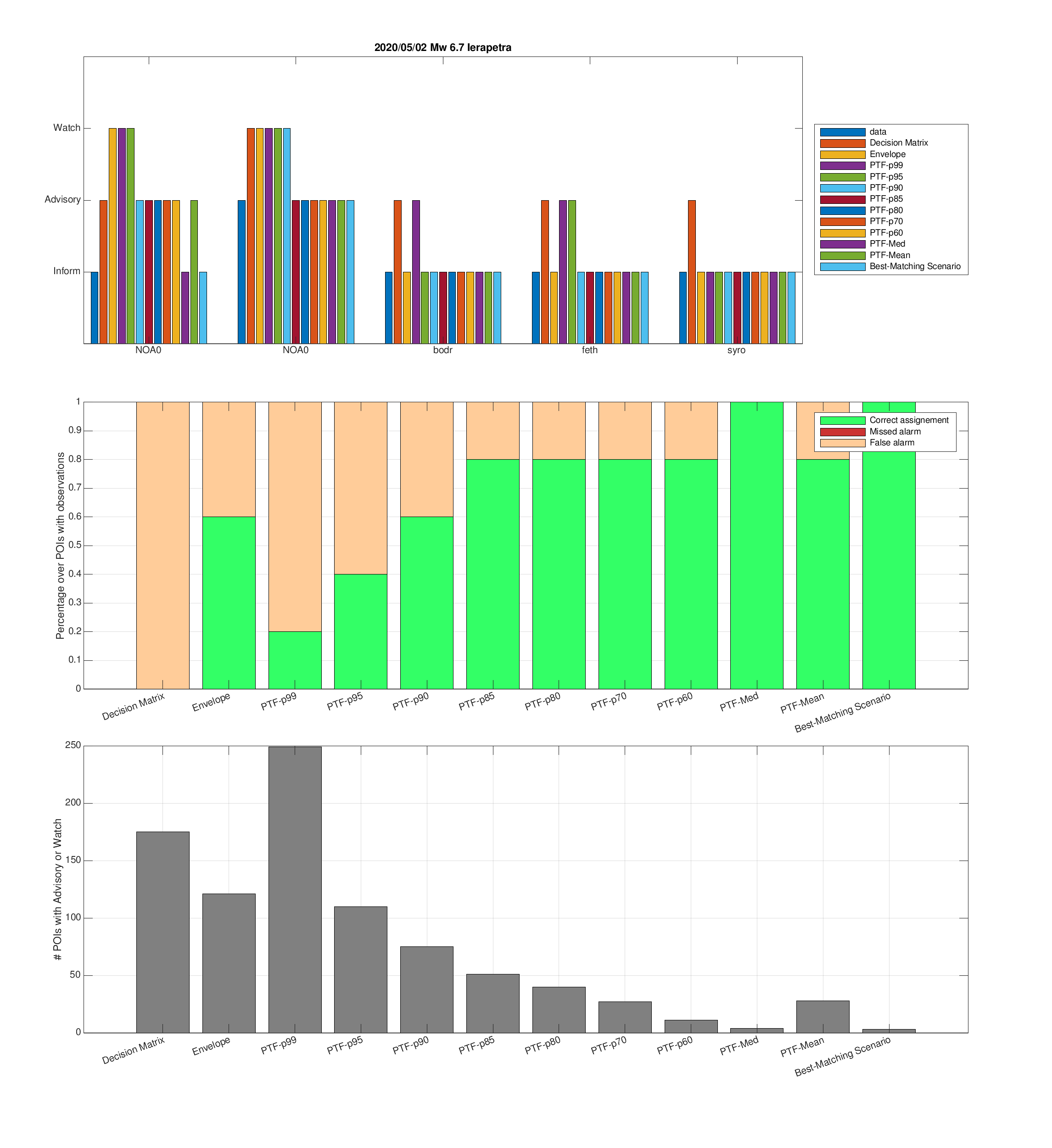

Supplement: Supplementary file 8 — Supplementary Dataset 5. Alert levels comparison figures. [file 41467_2021_25815_MOESM8_ESM.zip › Test4_2020_0502_crete_sig20_ALs.png]
