# Supplementary figures and images for: Probabilistic tsunami forecasting for early warning (part 2 of 2)
Source: Nat Commun. 2021 Sep 28;12:5677. doi: 10.1038/s41467-021-25815-w (PMC8479076; doi:10.1038/s41467-021-25815-w)

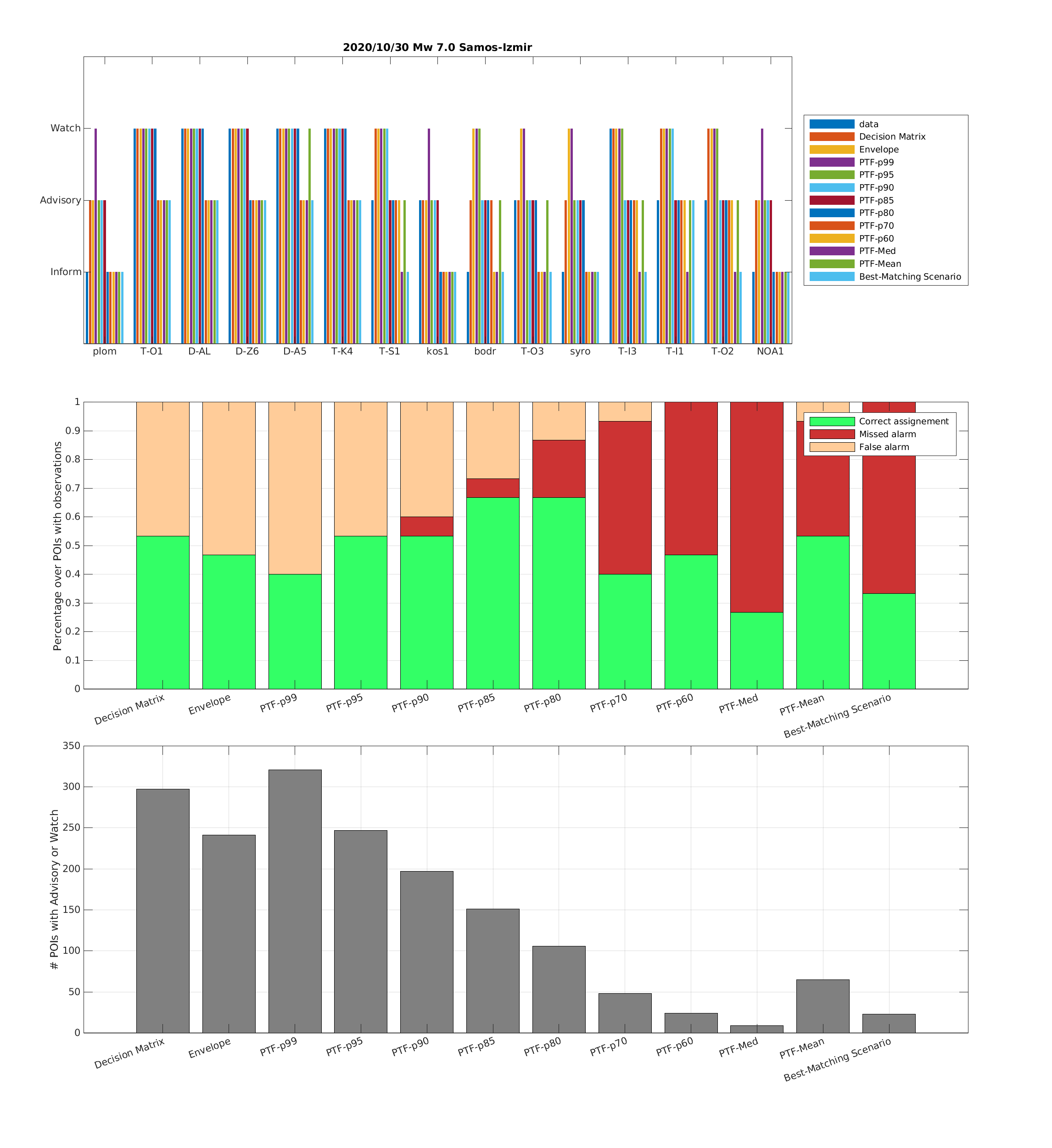

Supplement: Supplementary file 8 — Supplementary Dataset 5. Alert levels comparison figures. [file 41467_2021_25815_MOESM8_ESM.zip › Test4_2020_1030_samos_sig20_ALs.png]
